# Cascade aza-Wittig/6π-Electrocyclization in the Synthesis of 1,6-Dihydropyridines

**DOI:** 10.1021/acs.orglett.1c02099

**Published:** 2021-07-22

**Authors:** Vasiliki Polychronidou, Anna Krupp, Carsten Strohmann, Andrey P. Antonchick

**Affiliations:** † Max-Planck-Institut für Molekulare Physiologie, Abteilung Chemische Biologie, Otto-Hahn-Straße 11, 44227 Dortmund, Germany; ‡ Technische Universität Dortmund, Fakultät für Chemie und Chemische Biologie, Otto-Hahn- Straße 6, 44227 Dortmund, Germany; § Nottingham Trent University, Department of Chemistry and Forensics, Clifton Lane, NG11 8NS Nottingham, United Kingdom

## Abstract

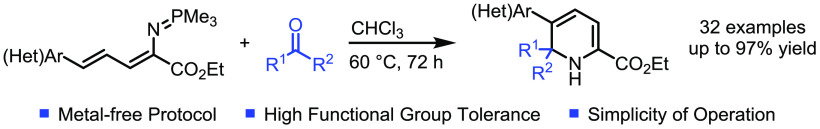

A metal-free protocol for the synthesis of substituted 1,6-dihydropyridines with quaternary stereogenic centers via a cascade aza-Wittig/6π-electrocyclization process has been developed. The high functional group compatibility and broad scope of this method were demonstrated by using a wide range of easily available vinyliminophosphoranes and ketones, with yields up to 97%. A modification of the obtained products allowed for an increase in complexity and chemical diversity. Finally, attempts for asymmetric synthesis of 1,6-dihydropyridines are demonstrated.

Dihydropyridines (DHPs) are a valuable chemical structure that can be found as the core scaffold in numerous compounds with varied biological and pharmacological activities.^1,2^ DHPs are also versatile synthetic intermediates due to their ability to undergo further chemical transformations, providing access to a variety of aza-heterocycles.^3^ Regarding the scaffold of dihydropyridines, 1,4- and 1,2- or 1,6-DHPs represent the most populated group whereas the latter has only recently gained significant attention.^4^ Several synthetic methods have been reported for the construction of 1,2-dihydropyridines by means of nucleophilic addition onto *N*-alkyl or *N*-acylpyridinium salts,^3a,5^ dearomatization of pyridines,^6^ transition-metal catalyzed reactions,^3c,7^ and the establishment of both Lewis acid^8^ and Brønsted acid catalyzed approaches^9^ (Scheme 1a). Furthermore, a pericyclic fashion was employed as an additional strategy to access these scaffolds. Palacios and co-workers have reported the synthesis of 1,2-dihydropyridines through a [4 + 2] cycloaddition reaction of 2-azadienes (readily prepared by aza-Wittig reactions) and enamines (Scheme 1b).^10^ Tejedor et al. has developed a convenient domino access to substituted alkyl 1,2-dihydropyridine-3-carboxylates from propargyl enol ethers and primary amines by means of a Claisen rearrangement/isomerization/amine condensation/6π-aza-electrocyclization process (Scheme 1c).^11^ Very recently, Yu, Zhou et al. reported the enantioselective synthesis of 1,2-dihydropyridines, using a chiral amine catalyst.^12^ Metal-free protocols that allow rapid access to substituted DHPs and their derivatives are in high demand.

**Scheme 1:**
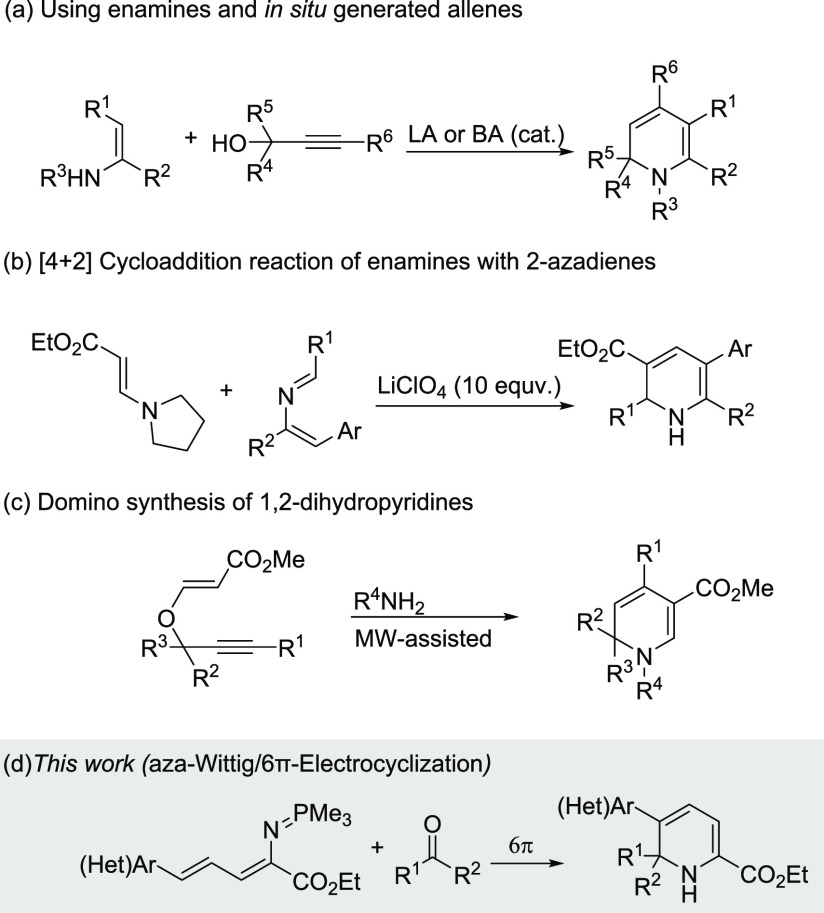
Synthesis of 1,2-Dihydropyridines

On the other hand, the incorporation of fluorine-containing derivatives into organic compounds is of high importance in pharmaceutical, agricultural, and material science. Introducing a C–F instead of a C–H bond in a molecule can modify its physical, chemical, and biological properties.^13^ Within these organofluorides, the trifluoromethyl group is considered one of the most important motifs. In particular, pyridinyl motifs with trifluoromethyl-substituents proved to have wide applications in different fields.^14^ To the best of our knowledge, there is no synthetic method that enables the construction of multisubstituted 1,2-dihydropyridines bearing mainly fluorinated all-carbon quaternary centers. Having interest in developing new methods for the synthesis and functionalization of heterocycles,^15^ herein we report a metal-free 6π-electrocyclic transformation of *in situ* generated aza-hexatrienes (Scheme 1d). The aza-hexatrienes are derived from an aza-Wittig reaction of phosphazenes with the corresponding carbonyl compounds.

To establish the reaction method, initial screening studies were conducted with different easily available *N*-vinylic-λ^5^-phosphazenes **1a** and 2,2,2-trifluoroacetophenone **2a**. As shown in Table 1, when the *N*-vinylic- λ^5^-phosphazene bearing a triphenylphosphine substituent was reacted with the trifluoromethyl ketone **2a**, in dichloromethane at ambient temperature, only the formation of the acyclic imine **3a** was observed in low yield (entry 1). Varying the substituent on the phosphorus atom of the *N*-vinylic- λ^5^-phosphazene, thus inducing changes of the electronic properties of the phosphorus, led to an increase in reactivity that resulted in improved yields of acyclic imine **3a**. However, the desired cyclized product **4a** was not observed at ambient temperature regardless of the reaction time (entries 2–4). Remarkably, changing the solvent to chloroform and heating the reaction to 60 °C yielded the desired product **4a**, albeit in low yield and with the acyclic imine still present (entry 5). Gratifyingly, by changing to the more reactive λ^5^-phosphazene bearing a trimethylphosphine substituent, the reaction proceeded smoothly and resulted to the cyclized product **4a** in 84% yield (entry 6). Further attempts, using toluene as solvent and high temperature did not improve the reaction outcome (entry 7).

**Table 1:**
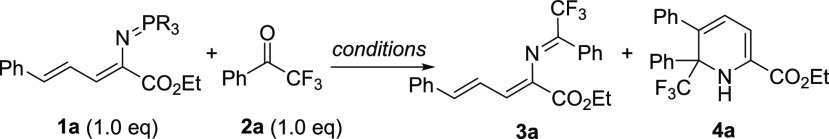
Optimization of Reaction Conditions^a^

					yield (%)^b^	
entry	PR_3_	solvent [0.1 M]	temp (°C)	time (h)	**3a**	**4a**
1	PPh_3_	CH_2_Cl_2_	rt	72	20	–
2	PPh_2_Me	CH_2_Cl_2_	rt	48	70	–
3	PMe_3_	CH_2_Cl_2_	rt	12	92	–
4	PMe_3_	CH_2_Cl_2_	rt	96	92	–
5	PPh_3_	CHCl_3_	60	72	25	30
**6**	**PMe**_**3**_	**CHCl**_**3**_	**60**	**72**	**–**	**84**
7	PMe_3_	PhMe	110	72	–	80

a Reaction conditions: **1a** (0.15 mmol) and **2a** (0.15 mmol) were stirred at given temperature for given time.

b Isolated yield.

With the optimized conditions in hand, we first explored the scope and limitations of our method by using a series of readily available ketones **2**. The aryl group of the ketone was systematically varied (Scheme 2). Both *para-* and *meta*-substituted ketones with electron-donating or electron-withdrawing groups were well tolerated and provided the desired products (**4a**–**4j**) in moderate to excellent yields (43–93%). In the case of *ortho-*substituted ketones, the yields were notably decreased and afforded the product **4k** in 24% yield due to the steric hindrance. It is noteworthy that, in the case of example **4l**, the standard conditions afforded mainly the noncyclized imine and only traces of the cyclized one. To obtain the desired cyclized product for this substrate, alternative conditions were used, in which the isolated acyclic imine in toluene was heated to 110 °C for 72 h. Strikingly, the scope could also be extended to heteroaryl-substituted ketones providing product **4m** in 60%. To our delight, this method was also applicable to ketones bearing difluoromethyl, chlorodifluoromethyl, and ethoxycarbonyl groups, affording the products (**4n**–**4p**) in moderate to excellent yields (48–97%). Remarkably, the 7-fluoroisatin could be used in our method, affording the valuable dihydropyridine-based spirooxindole **4q**, albeit in moderate yield, 45%. Unfortunately, when acetophenone and aliphatic trifluoromethyl ketones such as 1,1,1-trifluoroacetone were tested under the standard conditions, the reaction did not take place, probably due to isomerization of imine to enamine.

**Scheme 2:**
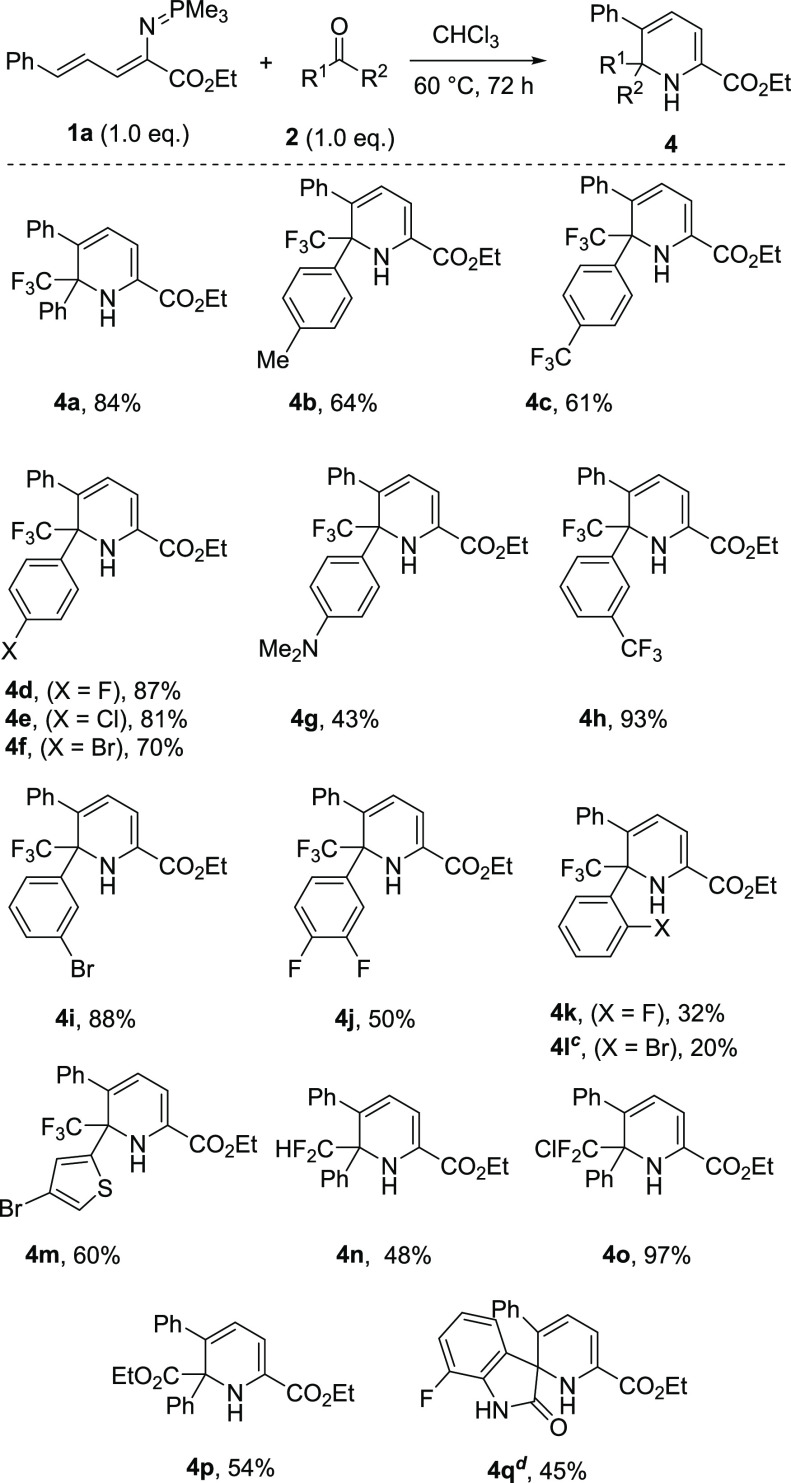
Substrate Scope with Different Ketones^,^ Reaction conditions: **1a** (0.15 mmol), **2** (0.15 mmol), in CHCl_3_ [0.1 M] were stirred at 60 °C for 72 h. Isolated yield. Reaction was conducted by heating the corresponding isolated noncyclized product in toluene, at 110 °C for 72 h. Isolated as inseparable mixture with the intermediate acyclic imine in a ratio of 4:1.

To rapidly expand the chemical space accessible via our method, we further explored the transformation with a series of substituted vinyliminiphosphoranes **1**. As illustrated in Scheme 3, a range of products **5** were obtained. Pleasingly, *ortho*-, *meta*-, and *para*-substituted phenyl rings were well tolerated. Moreover, the presence of electron-donating as well as electron-withdrawing substituents still resulted in high reactivity affording the desired products **5a**–**5l** in moderate to excellent yields (49–94%). Compound **5a** was obtained in a scale up experiment, and its structure was confirmed by X-ray crystallographic analysis. A similar trend was observed with the heterocycle-containing compounds yielding the products **5m** and **5k** in 89% and 83% yield, respectively. Notably, switching to vinyliminophosphorane bearing an aliphatic moiety, in this case methyl, led to a dramatic decrease in reactivity, and only traces of product **5o** were obtained. We tested whether a direct protocol which does not require the preparation of vinyliminophosphoranes is feasible. Unfortunately, a one-pot procedure, using vinyl-azide (precursor of compound **1**), trimethylphosphine, and ketone **2a** did not lead to the formation of the desired product.

**Scheme 3:**
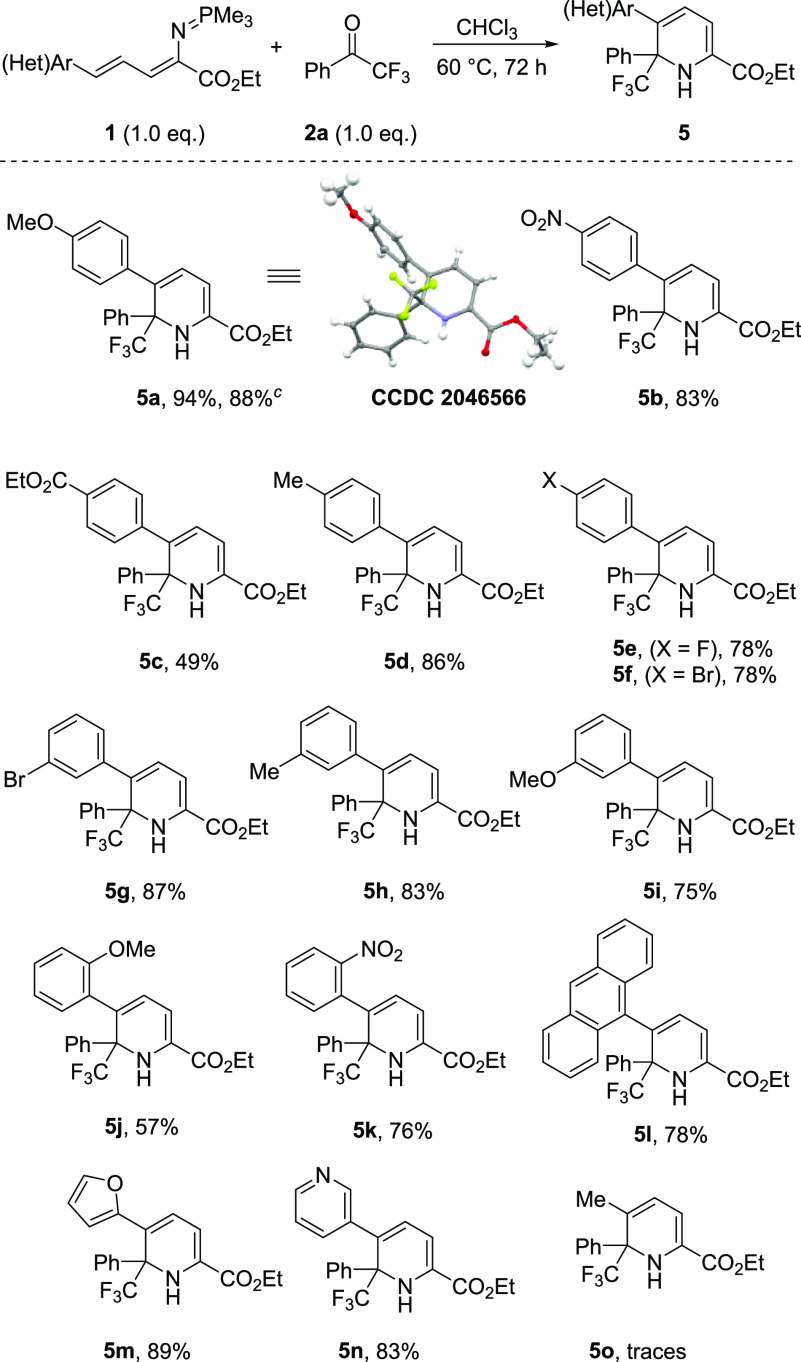
Substrate Scope with Different Vinyliminophosphoranes^,^ Reaction conditions: **1** (0.15 mmol), **2a** (0.15 mmol), in CHCl_3_ [0.1 M] were stirred at 60 °C for 72 h. Isolated yield. Isolated yield for 1.00 mmol scale.

Based on the above results and the reported literature^7f,16^ a putative reaction mechanism is proposed (Scheme 4). The reaction proceeds via an aza-Wittig reaction between the vinyliminophospharene **1a** and ketone **2a** to afford the corresponding azatriene **3a** through formation of imine and elimination of trimethyl phosphine oxide. The linear imine *s*-*trans, s*-*trans***3a** (confirmed by X-ray analysis, Scheme 4) must be converted to the “cyclization-reactive” *s*-*cis, s*-*cis* conformer **3a′** through bond rotations in order to undergo 1,6-electrocyclization. This isomerization to the reactive conformation is thermodynamically unfavored and can therefore be accessed under thermal conditions. The subsequent thermal 6π disrotatory electrocyclization provides the intermediate **A** which, by means of a [1,5]-hydride shift, results in the final product **4a**.

**Scheme 4:**
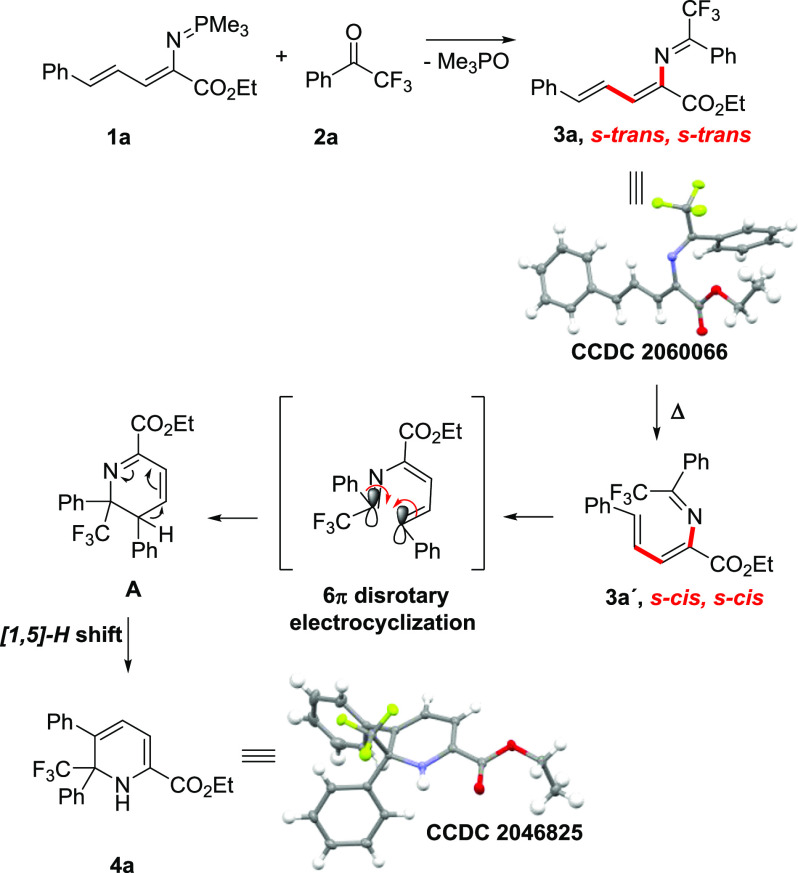
Proposed Reaction Mechanism

Furthermore, product **4a** could be converted onto its tetrahydropyridine **6a** or piperidine moiety **6b** as a single diastereomer upon reduction with hydrogen over Pd/C catalyst by simply changing the temperature and the reaction time (Scheme 5). Not only does this allow access to a novel range of compounds, but it also provides an option to explore a different dimension of chemical space.

**Scheme 5:**
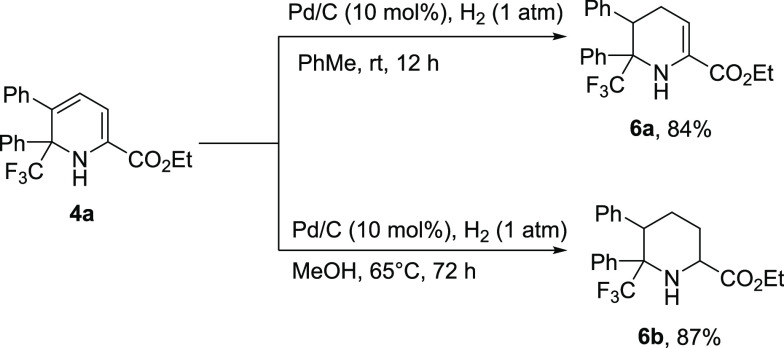
Further Transformation of **4a**

Finally, asymmetric 6π-electrocyclization reactions are a challenging task with several successful examples in literature.^17^ In this context, we performed a preliminary investigation on the catalytic asymmetric version of our method employing chiral Brønsted acids. As shown in Table 2, in the presence of several representative chiral phosphoric acids (**7** and **8**) and chiral disulfonimide **9**, the electrocyclization of substrate **3b** was achieved in generally good yields. However, the enantioselectivities in all cases were low (up to 34% *ee*). Among these catalysts, chiral phosphoric acid **7e** afforded product **5b** in comparatively lower yield but with a promising level of enantioselectivity (24%, entry 5). When decreasing the temperature from 60 to 50 °C, an increase in the enantioselectivity was observed, although it resulted in a lower yield (entry 6). A further decrease in temperature led to traces of product (entry 7). The low enantioselectivity of the reaction might be partly ascribed to the strong background reaction because the electrocyclization of substrate **3b** could occur in the absence of any catalysts (Table 1, entry 6).

**Table 2:**
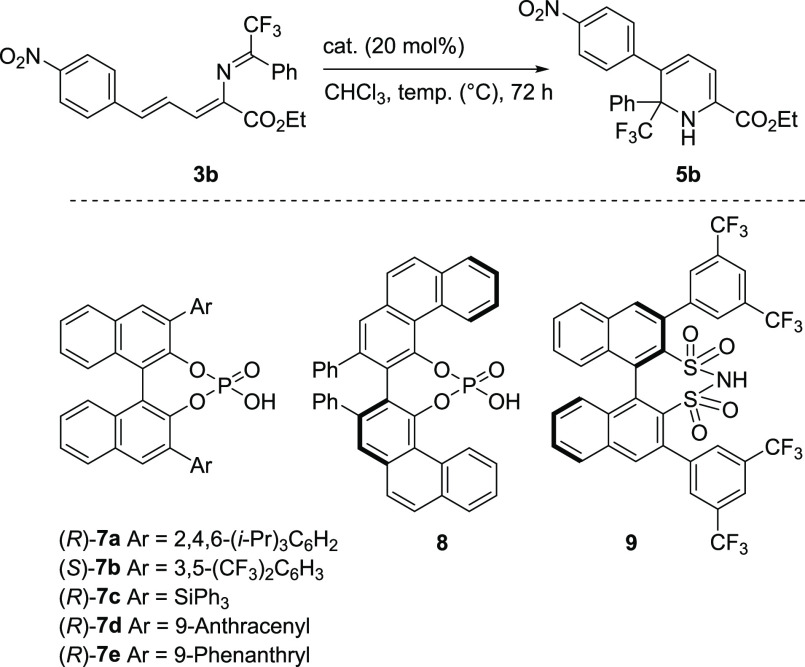
Preliminary Investigation of the Asymmetric Electrocyclization of Substrate **3b**^a^

entry	cat.	temp (°C)	yield (%)^b^	*ee* (%)^c^
1	(*R*)-**7a**	60	90	5
2	(*S*)-**7b**	60	88	3
3	(*R*)-**7c**	60	90	16
4	(*R*)-**7d**	60	70	13
5	(*R*)-**7e**	60	75	24
6	(*R*)-**7e**	50	40	34
7	(*R*)-**7e**	30	traces	–
8	**8**	60	80	3
9	**9**	60	85	2

a Reaction conditions: **3b** (0.025 mmol) in CHCl_3_ [0.1 M] using 20 mol % of chiral catalyst for 72 h.

b Isolated yield.

c Values of *ee* were determined using chiral HPLC.

In summary, we have developed a metal-free and efficient approach for the synthesis of unprecedented 1,6-dihydropyridines with quaternary stereocenters via an aza-Wittig/6π-electrocyclization process. This protocol provides an access to a new class of pyridine frameworks under mild reaction conditions, featuring good functional group tolerance and operational simplicity. These novel building blocks could access interesting bioactivities through a range of synthetic transformations. A plausible reaction mechanism for the developed cascade process is proposed. Finally, asymmetric synthesis of 1,6-dihydropyridines was studied using various Brønsted acids.

## Supporting Information Available

Experimental procedures, characterization data, and ^1^H, ^13^C, ^19^ F NMR spectra (PDF)

## Accession Codes

CCDC 2046566, 2046825, 2060066, and 2081540 contain the supplementary crystallographic data for this paper. These data can be obtained free of charge via www.ccdc.cam.ac.uk/data_request/cif, or by emailing data_request@ccdc.cam.ac.uk, or by contacting The Cambridge Crystallographic Data Centre, 12 Union Road, Cambridge CB2 1EZ, UK; fax: +44 1223 336033.
