# Supplementary material for: Cascade aza-Wittig/6π-Electrocyclization in the Synthesis of 1,6-Dihydropyridines
Source: Org Lett. 2021 Jul 22;23(15):6024–9. doi: 10.1021/acs.orglett.1c02099 (PMC8397428; doi:10.1021/acs.orglett.1c02099)
Supplement: Supplementary file 1 — ol1c02099_si_001.pdf [file ol1c02099_si_001.pdf]

Supporting Information

Cascade aza-Wittig/6π-Electrocyclization in the Synthesis of 1,6-Dihydropyridines

Vasiliki Polychronidou[1,2], Anna Krupp[2], Carsten Strohmann[2] and Andrey P. Antonchick*[1,2,3]

[1] Max-Planck-Institut für Molekulare Physiologie, Abteilung Chemische Biologie, Otto-Hahn-Straße 11, 44227 Dortmund, Germany

[2] Technische Universität Dortmund, Fakultät für Chemie und Chemische Biologie, Otto-Hahn- Straße 6, 44227 Dortmund, Germany

[3] Nottingham Trent University, Department of Chemistry and Forensics, Clifton Lane NG11 8NS Nottingham, United Kingdom

* E-mail: andrey.antonchick@ntu.ac.uk

<u>**Table of contents**</u>

1. General Information S3

2. Experimental Procedures S4

2.1. Preparation of Substrates S4

2.2. Synthesis of 1,6-Dihydropyridine-3-carboxylates S5

2.3. 1.00 mmol Scale Reaction for the Synthesis of Compound 5a S5

2.4. One-pot Reaction for the Synthesis of Compound 5i S5

2.5. Further Transformation of Compound 4a S6

2.6. General Procedure for Asymmetric Synthesis S6

3. Physical Data of Compounds S7

3.1. Physical Data of Vinyl Azides S7

3.2. Physical Data of 1,6-Dihydropyridines-3-carboxylates S14

3.3. Physical Data of Compounds 6a and 6b S35

4. X-ray Analysis of Compounds 3a, 3b, 4a and 5a S37

5. Copies of NMR spectra S43

6. Copies of Selected Chiral HPLC Chromatograms S108

7. References S112

1. General Information

Unless otherwise noted, all commercially available compounds were used as received without further purifications. Dry solvents were purchased from Acros or Sigma Aldrich and used without further treatment. Solvents for chromatography were technical grade. Analytical thin-layer chromatography (TLC) was performed on Merck silica gel aluminum plates with F-$_{254}$ indicator, visualized by irradiation with UV light. Column chromatography was performed using silica gel Merck 60 (particle size 0.040 - 0.063 mm).

^{1}H-NMR and ^{13}C-NMR were recorded on a Bruker DRX400 (400 MHz), Bruker DRX500 (500 MHz), INOVA500 (500 MHz) and Bruker DRX700 (700 MHz) using $CDCl_3$ as solvent. Data are reported in the following order: chemical shift (δ) values are reported in ppm with the solvent resonance as internal standard ($CDCl_3$: δ = 7.26 ppm for ^{1}H, δ = 77.16 ppm for ^{13}C); multiplicities are indicated s (singlet), d (doublet), t (triplet), q (quartet), m (multiplet); coupling constants (*J*) are given in Hertz (Hz).

Fourier transform infrared spectroscopy (FT-IR) spectra were obtained with a Bruker Tensor 27 spectrometer (ATR, neat) and are reported in terms of frequency of absorption (cm^{-1}).

High resolution mass spectra (HR-MS) were recorded on a LTQ Orbitrap mass spectrometer coupled to an Accela HPLC-System (HPLC column: Hypersyl GOLD, 50 mm x 1 mm, particle size 1.9 µm, ionization method: electron spray ionization).

The enantiomeric excesses were determined by HPLC analysis using a chiral stationary phase column (CHIRALCEL IC; eluent: (*iso*-hexane / *i*-PrOH = 100/2); 4.6 mm x 250 mm, particle size 5 µm). The chiral HPLC methods were calibrated with the corresponding racemic mixtures.

2. Experimental Procedures

2.1. Preparation of Substrates

General Procedure for the Preparation of Vinyl Azides

The requisite vinyl azides were prepared in one step from the condensation of ethyl azidoacetate and aromatic or heteroaromatic cinnamaldehydes following the method reported by Moody and co-workers.[1] The yields were not optimized.

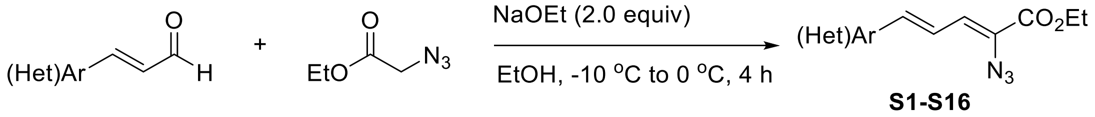

A solution of ethyl azidoacetate[2] (2.20 equiv) and trans-α, β-unsaturated aldehydes (1.00 equiv) in anhydrous ethanol [3 M] were added dropwise to a well-stirred solution containing sodium ethoxide (21 wt % solution in ethanol, 2.20 equiv) in anhydrous ethanol [2.5 M] at -10 °C under nitrogen atmosphere. The reaction mixture was stirred at 0 °C for 4 h. After reaction completion, the mixture was diluted with water. The phases were separated, and the resulting aqueous phase was extracted with ethyl acetate. The combined organic layers were washed with water and brine, dried over $MgSO_4$, and concentrated in vacuum. The crude reaction mixture was purified by means of short silica-gel column chromatography (eluent system: *n*-pentane – ethyl acetate) to afford the pure vinyl azides (**S1-S16**).

General Procedure for the Preparation of Vinyliminophosphoranes

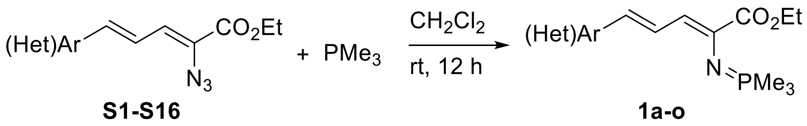

A solution of the corresponding vinyl azide (**S1 – S16)** (1.00 equiv) in anhydrous dichloromethane [0.4 M] was added dropwise to a solution of trimethylphosphine (1 M solution in toluene, 1.10 equiv) in anhydrous dichloromethane [0.2 M] at room temperature and stirred for 12 h. Upon reaction completion, the solvent was removed under reduced pressure. The compounds **1a-o** were used without further purification for the following reactions.

2.2. Synthesis of 1,6-Dihydropyridine-3-carboxylates

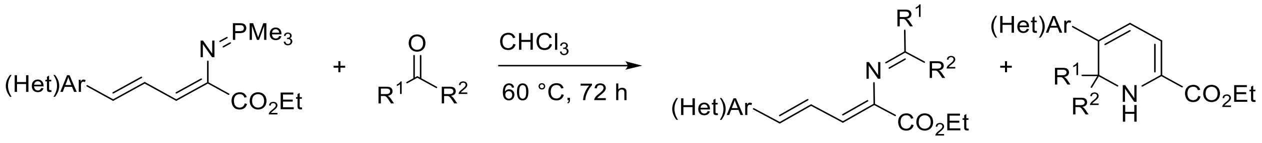

1a-o **2** **3a-o** **4a-4q & 5a-o**

General Procedure. Unless otherwise mentioned, to a 5.0 mL screw-capped seal tube were successively added a solution of compound **1** (0.15 mmol, 1.00 equiv) in anhydrous chloroform [0.1 M] and the respective ketone (0.15 mmol, 1.00 equiv). Then the tube was sealed, and the resulting mixture was stirred vigorously at 60 °C (on oil bath) till almost full consumption of imine, monitored by TLC analysis. Upon reaction completion, the solvent was evaporated, and the resulting crude was purified by means of short silica-gel column chromatography (eluent system: *n*-pentane - EtOAc) to afford the pure product.

2.3. 1.00 mmol Scale Reaction for the Synthesis of Compound 5a

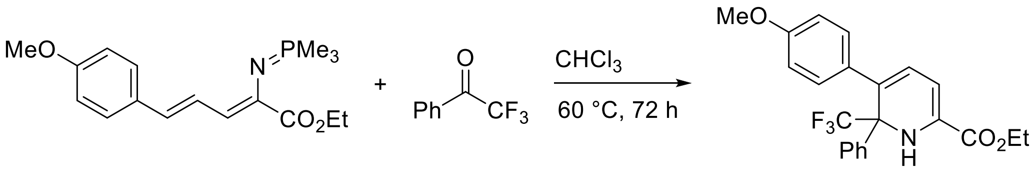

1a´, 1.00 mmol **2a** **5a**

To a 50.0 mL round-bottom flask, were successively added a solution of compound **1a** (378.06 mg, 1.00 mmol, 1.00 equiv) in anhydrous chloroform (10.0 mL) and 2,2,2-trifluoroacetophenone (141.84 µL, 1.00 mmol, 1.00 equiv). Then the flask was equipped with a findenser, and the resulting mixture was stirred vigorously at 60 °C (on oil bath). Upon reaction completion as monitored by TLC, the solvent was evaporated and the resulting crude was purified by means of short silica-gel column chromatography (eluent: 2% EtOAc in *n*-pentane) to afford the pure product as yellow amorphous solid (354.2 mg, 0.88 mmol, 88%).

2.4. One-pot Reaction for the Synthesis of Compound 5i

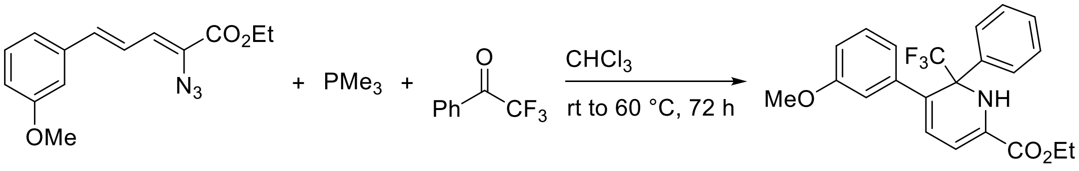

S8 **2a** **5i**

To a 5.0 mL screw-capped seal tube were successively added compound **S8** (50.0 mg, 0.18 mmol, 1.00 equiv) and trimethylphospine (1 M solution in toluene, 201.3 μL, 1.10 equiv) and dissolved in anhydrous chloroform (2.0 mL). After stirring for 5 min at room temperature, 2,2,2-trifluoroacetophenone (26.0 μL, 1.00 mmol, 1.00 equiv) was added and the reaction mixture was heated up to 60 °C (on oil bath). The reaction as monitored by TLC and GC-MS analysis did not lead to the formation of product **5i**.

2.5. Further Transformation of Compound 4a

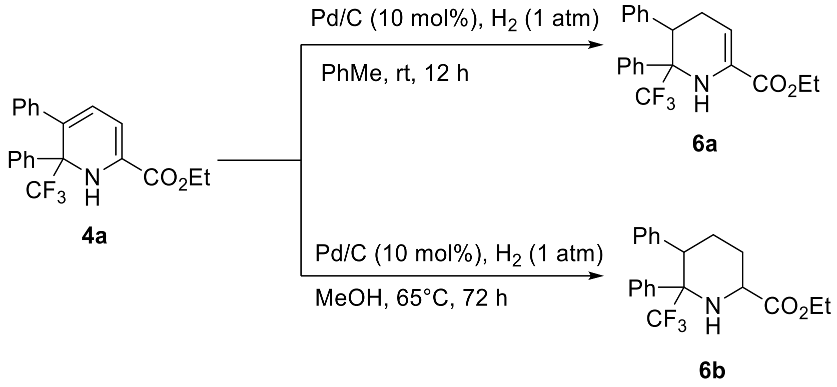

Procedure for synthesis of 6a. A solution of substrate **4a** (0.05 mmol, 20.00 mg, 1.00 equiv.) in toluene [0.05 M] was treated with Pd/C (50-100 wt.%) (0.005 mmol, 5.7 mg, 10 mol%) and then the reaction mixture was saturated with hydrogen gas (two cycles). The solution was then stirred at room temperature under hydrogen atmosphere for 12 h. The reaction was filtered through celite and washed with methanol (2 × 5 mL). Evaporation of the solvent afforded the expected product **6a** (17.0 mg, 0.045 mmol, 84% yield).

Procedure for synthesis of 6b. A solution of substrate **4a** (0.08 mmol, 28.00 mg, 1.00 equiv.) in methanol [0.05 M] was treated with Pd/C (50-100 wt.%) (0.01 mmol, 8.10 mg, 10 mol%) and then the reaction mixture was saturated with hydrogen gas (two cycles). The solution was then stirred at 65 °C (oil bath) under hydrogen atmosphere for 72 h. The reaction was filtered through celite and washed with methanol (2 × 5 mL). Evaporation of the solvent afforded the expected product **6b** (17.5 mg, 0.05 mmol, 87% yield).

2.6. General Procedure for Asymmetric Synthesis

To a 2.0 mL screw-capped glass tube were successively added the acyclic imine **3** (10 mg, 0.024 mmol, 1.00 equiv.) and the corresponding catalyst (0.005 mmol, 20 mol%) in chloroform [0.1 M]. Then the tube was sealed, and the resulting mixture was stirred vigorously at 60 °C (on oil bath) till almost full consumption of imine by TLC analysis. Upon reaction completion, the solvent was evaporated, and the

resulting crude was purified by means of short silica-gel column chromatography (eluent system: *n*-pentane - EtOAc) and analyzed by HPLC on a chiral stationary phase.

3. Physical Data of Compounds

3.1. Physical Data of Vinyl Azides

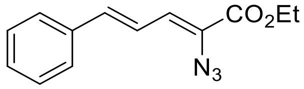

Ethyl (2*Z*, 4*E*) -2-azido-5-phenylpenta-2,4-dienoate (S1)

The general procedure was followed and purification by column chromatography (2% ethyl acetate in *n*-pentane) afforded **S1** as a light-yellow solid, (1.7 g, 7.1 mmol, 47% yield).

^{1}H NMR (400 MHz, $CDCl_3$) δ 7.44 – 7.39 (m, 2H), 7.31 – 7.25 (m, 2H), 7.25 – 7.19 (m, 1H), 7.10 (dd, *J* = 15.7, 11.2 Hz, 1H), 6.79 – 6.64 (m, 2H), 4.26 (q, *J* = 7.1 Hz, 2H), 1.30 (t, *J* = 7.1 Hz, 3H) ppm.

^{13}C NMR (101 MHz, $CDCl_3$) δ 163.3, 139.1, 136.5, 129.1, 128.9, 127.4, 127.0, 125.9, 122.4, 62.1, 14.4 ppm.
The analytical data were in good accordance with those reported in the literature.[3]

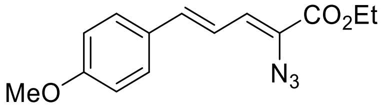

Ethyl (2*Z*, 4*E*) -2-azido-5-(4-methoxyphenyl) penta-2,4-dienoate (S2)

The general procedure was followed and purification by column chromatography (4% ethyl acetate in *n*-pentane) afforded **S2** as a light-yellow solid, (1.0 g, 3.8 mmol, 31% yield). The analytical data were in good accordance with those reported in the literature.[4]
^{1}H NMR (500 MHz, $CDCl_3$) δ 7.36 (d, *J* = 8.7 Hz, 2H), 6.96 (dd, *J* = 15.6, 11.3 Hz, 1H), 6.81 (d, *J* = 8.8 Hz, 2H), 6.73 – 6.62 (m, 2H), 4.25 (q, *J* = 7.1 Hz, 2H), 3.76 (s, 3H), 1.30 (t, *J* = 7.1 Hz, 3H) ppm.

^{13}C NMR (126 MHz, $CDCl_3$) δ 163.4, 160.5, 138.9, 129.4, 128.9, 127.7, 124.7, 120.3, 114.4, 62.0, 55.5, 14.4 ppm.

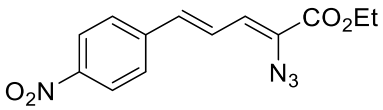

Ethyl (2*Z*,4*E*) -2-azido-5-(4-nitrophenyl) penta-2,4-dienoate (S3)

The general procedure was followed and purification by column chromatography (4% ethyl acetate in *n*-pentane) afforded **S3** as a bright orange solid, (1.0 g, 3.5 mmol, 32% yield). The analytical data were in good accordance with those reported in the literature.[4b, 5]

^{1}H NMR (500 MHz, $CDCl_3$) δ 8.20 (d, *J* = 8.8 Hz, 2H), 7.60 (d, *J* = 8.8 Hz, 2H), 7.31 (dd, *J* = 15.7, 11.2 Hz, 1H), 6.83 (d, *J* = 15.7 Hz, 1H), 6.72 (d, *J* = 11.1 Hz, 1H), 4.35 (q, *J* = 7.1 Hz, 2H), 1.39 (t, *J* = 7.1 Hz, 3H) ppm.

^{13}C NMR (126 MHz, $CDCl_3$) δ 162.9, 142.8, 135.6, 128.4, 127.7, 126.5, 125.0, 124.7, 124.3, 62.5, 14.3 ppm.

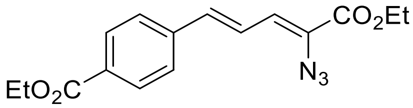

Ethyl 4-((1*E*, 3*Z*) -4-azido-5-ethoxy-5-oxopenta-1,3-dien-1-yl) benzoate (S4)

The general procedure was followed and purification by column chromatography (4% ethyl acetate in *n*-pentane) afforded **S4** as a bright orange solid, (0.94 g, 3.0 mmol, 28% yield).

^{1}H NMR (500 MHz, $CDCl_3$) δ 8.01 (d, *J* = 8.4 Hz, 2H), 7.52 (d, *J* = 8.0 Hz, 2H), 7.37 – 7.16 (m, 1H), 6.84 (s, 1H), 6.74 (dd, *J* = 11.3, 0.9 Hz, 1H), 4.36 (dq, *J* = 18.5, 7.1 Hz, 4H), 1.39 (dt, *J* = 9.3, 7.1 Hz, 6H)

^{13}C NMR (126 MHz, $CDCl_3$) δ 166.3, 163.1, 140.7, 137.5, 130.4, 130.2, 127.1, 127.1, 126.0, 124.6, 62.3, 61.2, 14.5, 14.3 ppm.

HRMS (ESI): calcd. for $[M+H-N_2]^+$ $C_{16}H_{18}NO_4$ = 288.1230; found 288.1235.

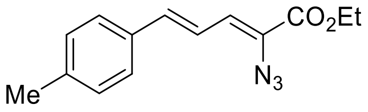

Ethyl (2*Z*, 4*E*) -2-azido-5-(p-tolyl) penta-2,4-dienoate (S5)

The general procedure was followed and purification by column chromatography (2% ethyl acetate in *n*-pentane) afforded **S5** as a light-yellow solid, (1.9 g, 7.5 mmol, 58% yield). The analytical data were in good accordance with those reported in the literature.[12]

^{1}H NMR (500 MHz, CDCl$_3$) δ 7.38 (d, J = 8.1 Hz, 2H), 7.16 (d, J = 7.9 Hz, 2H), 7.13 – 7.05 (m, 1H), 6.79 (d, J = 15.7 Hz, 1H), 6.75 (dd, J = 11.3, 0.9 Hz, 1H), 4.33 (q, J = 7.1 Hz, 2H), 2.36 (s, 3H), 1.37 (t, J = 7.1 Hz, 3H) ppm.

^{13}C NMR (126 MHz, CDCl$_3$) δ 163.2, 139.2, 139.1, 133.7, 129.6, 127.2, 125.2, 121.3, 77.3, 61.9, 21.4, 14.3 ppm.

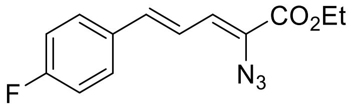

Ethyl (2*Z*, 4*E*)-2-azido-5-(4-fluorophenyl) penta-2,4-dienoate (S6)

The general procedure was followed and purification by column chromatography (2% ethyl acetate in *n*-pentane) afforded **S6** as a light-yellow solid, (1.2 g, 4.6 mmol, 36% yield).

^{1}H NMR (500 MHz, CDCl$_3$) δ 7.46 (dd, J = 8.8, 5.4 Hz, 2H), 7.15 – 7.00 (m, 3H), 6.84 – 6.69 (m, 2H), 4.33 (q, J = 7.1 Hz, 2H), 1.37 (t, J = 7.1 Hz, 3H) ppm.

^{13}C NMR (126 MHz, CDCl$_3$) δ 163.2, 163.2 (d, J_{CF} = 249.8 Hz), 137.6, 132.8 (d, J_{CF} = 3.4 Hz), 129.0 (d, J_{CF}= 8.2 Hz), 126.7, 125.9, 122.1 (d, J_{CF} = 2.5 Hz), 116.0 (d, J_{CF} = 21.8 Hz), 62.2, 14.4 ppm.

^{19}F NMR (470 MHz, CDCl$_3$) δ -111.64 (s, 1F) ppm.

HRMS (ESI): calcd. for $[M+H-N_2]^+$ $C_{13}H_{13}FNO_2$ = 284.0925; found 284.0925.

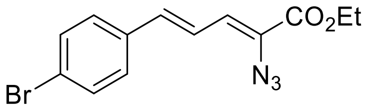

Ethyl (2*Z*, 4*E*)-2-azido-5-(4-bromophenyl) penta-2,4-dienoate (S7)

The general procedure was followed and purification by column chromatography (2% ethyl acetate in *n*-pentane) afforded **S7** as a light-yellow solid, (1.3 g, 4.0 mmol, 44% yield). The analytical data were in good accordance with those reported in the literature.[4a, 13]

^{1}H NMR (500 MHz, CDCl$_3$) δ 7.47 (d, J = 8.5 Hz, 2H), 7.34 (d, J = 8.5 Hz, 2H), 7.15 (dd, J = 15.6, 11.3 Hz, 1H), 6.79 – 6.66 (m, 2H), 4.33 (q, J = 7.1 Hz, 2H), 1.37 (t, J = 7.1 Hz, 3H) ppm.

^{13}C NMR (126 MHz, CDCl$_3$) δ 163.2, 137.5, 135.5, 132.1 (2C), 128.7 (2C), 126.4, 123.0, 62.2, 14.3 ppm.

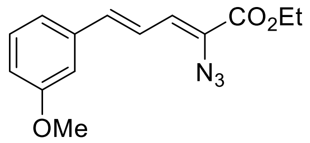

Ethyl (2*Z*, 4*E*)-2-azido-5-(3-methoxyphenyl) penta-2,4-dienoate (S8)

The general procedure was followed and purification by column chromatography (4% ethyl acetate in *n*-pentane) afforded **S8** as a light-yellow solid, (1.3 g, 4.8 mmol, 41% yield).

^{1}H NMR (500 MHz, $CDCl_3$) δ 7.29 – 7.22 (m, 1H), 7.14 (dd, *J* = 15.7, 11.2 Hz, 1H), 7.07 (d, *J* = 7.6 Hz, 1H), 7.00 (t, *J* = 2.1 Hz, 1H), 6.85 (ddd, *J* = 8.2, 2.6, 0.9 Hz, 1H), 6.81 – 6.70 (m, 2H), 4.33 (q, *J* = 7.1 Hz, 2H), 3.83 (s, 3H), 1.37 (t, *J* = 7.1 Hz, 3H) ppm.

^{13}C NMR (126 MHz, $CDCl_3$) δ 168.4, 160.0, 138.9, 137.9, 129.9, 126.8, 125.9, 122.6, 120.2, 114.9, 112.2, 62.0, 50.5, 14.3 ppm.

HRMS (ESI): calcd. for $[M+H-N_2]^+$ $C_{14}H_{16}NO_3$ = 246.1125; found 246.1125.

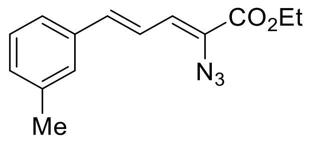

Ethyl (2*Z*, 4*E*)-2-azido-5-(m-tolyl) penta-2,4-dienoate (S9)

The general procedure was followed and purification by column chromatography (2% ethyl acetate in *n*-pentane) afforded **S9** as a light-yellow solid, (1.2 g, 4.7 mmol, 36% yield). The analytical data were in good accordance with the methyl-ester derivative, reported in the literature.[4a]

^{1}H NMR (500 MHz, $CDCl_3$) δ 7.33 – 7.21 (m, 3H), 7.19 – 7.10 (m, 2H), 6.79 (d, *J* = 15.7 Hz, 1H), 6.75 (dd, *J* = 11.2, 0.7 Hz, 1H), 4.34 (q, *J* = 7.1 Hz, 2H), 2.37 (s, 3H), 1.38 (t, *J* = 7.1 Hz, 3H) ppm.

^{13}C NMR (126 MHz, $CDCl_3$) δ 163.30, 139.29, 138.55, 136.44, 129.96, 128.82, 127.91, 127.13, 125.62, 124.70, 122.17, 62.10, 21.50, 14.35 ppm.

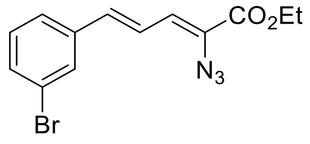

Ethyl (2*Z*, 4*E*) -2-azido-5-(3-bromophenyl) penta-2,4-dienoate (S10)

The general procedure was followed and purification by column chromatography (2% ethyl acetate in *n*-pentane) afforded **S10** as a light-yellow solid, (0.85 g, 2.6 mmol, 29% yield).

^{1}H NMR (500 MHz, CDCl$_3$) δ 7.62 (t, *J* = 1.8 Hz, 1H), 7.44 – 7.35 (m, 2H), 7.22 (t, *J* = 7.9 Hz, 1H), 7.19 – 7.10 (m, 1H), 6.73 (d, *J* = 3.8 Hz, 1H), 6.70 (s, 1H), 4.34 (q, *J* = 7.1 Hz, 2H), 1.38 (t, *J* = 7.1 Hz, 3H) ppm.

^{13}C NMR (126 MHz, CDCl$_3$) δ 163.1, 138.7, 137.0, 131.8, 130.4, 130.0, 126.8, 126.1, 125.9, 123.7, 123.1, 62.3, 14.3 ppm.

HRMS (ESI): calcd. for $[M+H-N_2]^+$ $C_{13}H_{13}{}^{79}BrNO_2$ = 294.0124; found 294.0126, calcd. for $[M+H-N_2]^+$ $C_{13}H_{13}{}^{81}BrNO_2$ = 296.0104; found 296.0106.

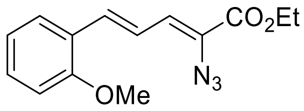

Ethyl (2*Z*, 4*E*)-2-azido-5-(2-methoxyphenyl) penta-2,4-dienoate (S11)

The general procedure was followed and purification by column chromatography (4% ethyl acetate in *n*-pentane) afforded **S11** as a light-yellow solid, (1.7 g, 6.3 mmol, 52% yield). The analytical data were in good accordance with those reported in the literature.[3]

^{1}H NMR (500 MHz, CDCl$_3$) δ 7.63 (dd, *J* = 7.8, 1.7 Hz, 1H), 7.37 – 7.29 (m, 2H), 7.29 – 7.21 (m, 1H), 7.06 – 6.99 (m, 1H), 6.95 (d, *J* = 7.9 Hz, 1H), 6.86 (dd, *J* = 7.2, 3.0 Hz, 1H), 4.39 (q, *J* = 7.1 Hz, 2H), 3.94 (s, 3H), 1.43 (t, *J* = 7.1 Hz, 3H) ppm.

^{13}C NMR (126 MHz, CDCl$_3$) δ 163.4, 157.5, 134.1, 130.3, 128.1, 127.4, 125.5, 125.1, 122.7, 120.9, 111.1, 62.0, 55.7, 14.4 ppm.

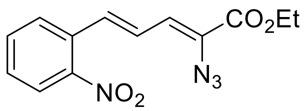

Ethyl (2*Z*, 4*E*) -2-azido-5-(2-nitrophenyl) penta-2,4-dienoate (S12)

The general procedure was followed and purification by column chromatography (4% ethyl acetate in *n*-pentane) afforded **S12** as a light-yellow solid, (0.90 g, 3.1 mmol, 28% yield). The analytical data were in good accordance with those reported in the literature.[3]

^{1}H NMR (500 MHz, CDCl$_3$) δ 7.95 (dd, *J* = 8.2, 1.3 Hz, 1H), 7.75 (dd, *J* = 8.0, 1.3 Hz, 1H), 7.60 (td, *J* = 7.7, 1.4 Hz, 1H), 7.44 (ddd, *J* = 8.4, 7.3, 1.3 Hz, 1H), 7.30 (d, *J* = 15.6 Hz, 1H), 7.16 (dd, *J* = 15.6, 11.0 Hz, 1H), 6.76 (dd, *J* = 11.1, 0.8 Hz, 1H), 4.35 (q, *J* = 7.1 Hz, 2H), 1.39 (t, *J* = 7.1 Hz, 3H) ppm.

^{13}C NMR (126 MHz, CDCl$_3$) δ 162.9, 148.2, 133.3, 132.6, 132.0, 129.1, 128.4, 128.0, 127.0, 125.4, 125.0, 62.4, 14.3 ppm.

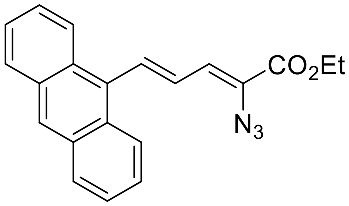

Ethyl (2*Z*, 4*E*)-5-(anthracen-9-yl)-2-azidopenta-2,4-dienoate (S13)

The general procedure was followed and purification by column chromatography (2% ethyl acetate in *n*-pentane) afforded **S13** as a bright orange solid, (0.62 g, 1.8 mmol, 21% yield).

^{1}H NMR (500 MHz, CDCl$_3$) δ 8.42 (s, 1H), 8.30 – 8.19 (m, 2H), 8.06 – 7.96 (m, 3H), 7.74 (d, *J* = 15.4 Hz, 1H), 7.53 – 7.46 (m, 4H), 7.16 – 6.95 (m, 1H), 4.40 (q, *J* = 7.2 Hz, 2H), 1.44 (t, *J* = 7.2 Hz, 3H) ppm.

^{13}C NMR (126 MHz, CDCl$_3$) δ 163.3, 135.5, 131.5, 131.4, 131.2, 129.6, 129.0, 128.6, 127.7, 126.5, 126.2, 125.6, 125.4, 62.3, 14.4 ppm.

HRMS (ESI): calcd. for $[M+H-N_2]^+$ $C_{21}H_{18}NO_2$ = 316.1332; found 316.1333.

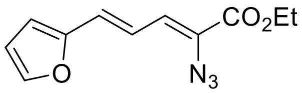

Ethyl (2*Z*, 4*E*)-2-azido-5-(furan-2-yl) penta-2,4-dienoate (S14)

The general procedure was followed and purification by column chromatography (2% ethyl acetate in *n*-pentane) afforded **S14** as a light-yellow solid, (1.9 g, 8.2 mmol, 51% yield). The analytical data were in good accordance with those reported in the literature.[3]

^{1}H NMR (500 MHz, CDCl$_3$) δ 7.44 (s, 1H), 7.03 (dd, *J* = 15.6, 11.5 Hz, 1H), 6.68 (dd, *J* = 11.6, 0.9 Hz, 1H), 6.59 (d, *J* = 15.6 Hz, 1H), 6.43 (td, *J* = 4.0, 3.4, 2.5 Hz, 2H), 4.32 (q, *J* = 7.1 Hz, 2H), 1.37 (t, *J* = 7.1 Hz, 3H) ppm.

^{13}C NMR (126 MHz, CDCl$_3$) δ 163.2, 152.8, 143.8, 126.5, 125.7, 125.5, 120.8, 112.3, 111.7, 62.1, 14.3 ppm.

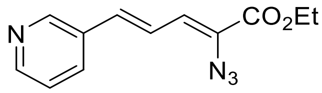

Ethyl (2*Z*, 4*E*)-2-azido-5-(pyridin-3-yl) penta-2,4-dienoate (S15)

The general procedure was followed and purification by column chromatography (10% ethyl acetate in *n*-pentane) afforded **S15** as a bright orange solid, (0.70 g, 2.9 mmol, 19% yield). The analytical data were in good accordance with the methyl-ester derivative, reported in the literature.[7]
^{1}H NMR (500 MHz, CDCl$_3$) δ 8.65 (d, *J* = 1.7 Hz, 1H), 8.51 (dd, *J* = 4.9, 1.6 Hz, 1H), 7.83 (dt, *J* = 8.0, 2.0 Hz, 1H), 7.30 (dd, *J* = 8.0, 4.8 Hz, 1H), 7.22 (dd, *J* = 15.8, 11.2 Hz, 1H), 6.82 – 6.56 (m, 2H), 4.34 (q, *J* = 7.1 Hz, 2H), 1.38 (t, *J* = 7.1 Hz, 3H) ppm.

^{13}C NMR (126 MHz, CDCl$_3$) δ 163.0, 149.5, 149.1, 134.6, 133.3, 132.4, 127.1, 125.7, 124.4, 123.9, 62.3, 14.3 ppm.

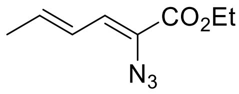

Ethyl (2*Z*, 4*E*) -2-azidohexa-2,4-dienoate (S16)

The general procedure was followed and purification by column chromatography (10% ethyl acetate in *n*-pentane) afforded **S16** as a light-yellow oil, (2.0 g, 11.0 mmol, 39% yield). The analytical data were in good accordance with those reported in the literature.[3]
^{1}H NMR (500 MHz, CDCl$_3$) δ 6.56 (d, *J* = 11.1 Hz, 1H), 6.49 – 6.39 (m, 1H), 6.12 – 6.02 (m, 1H), 4.29 (q, *J* = 7.1 Hz, 2H), 1.86 (dd, *J* = 6.9, 1.7 Hz, 3H), 1.34 (t, *J* = 7.1 Hz, 3H) ppm.

^{13}C NMR (126 MHz, CDCl$_3$) δ 163.5, 138.4, 127.4, 125.9, 123.9, 61.9, 19.1, 14.3 ppm.

3.2. Physical Data of 1,6-Dihydropyridines-3-carboxylates

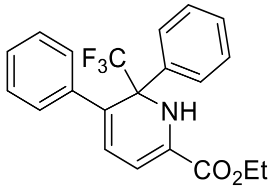

Ethyl 5,6-diphenyl-6-(trifluoromethyl)-1,6-dihydropyridine-2-carboxylate (4a)

Prepared according to the general procedure **A**. The product was isolated as yellow amorphous solid (45 mg, 0.12 mmol, 84% yield) (eluent: 1% EtOAc in *n*-pentane).

^{1}H NMR (500 MHz, CDCl$_3$) δ 7.61 (d, *J* = 8.3 Hz, 2H), 7.42 – 7.33 (m, 3H), 7.15 (t, *J* = 7.4 Hz, 1H), 7.08 (t, *J* = 7.5 Hz, 2H), 6.74 (d, *J* = 7.2 Hz, 2H), 6.35 (d, *J* = 6.5 Hz, 1H), 6.00 (dd, *J* = 6.5, 1.8 Hz, 1H), 5.06 (s, 1H), 4.29 (q, *J* = 7.1 Hz, 2H), 1.34 (t, *J* = 7.1 Hz, 3H) ppm.

^{13}C NMR (126 MHz, CDCl$_3$) δ 163.5, 140.7, 139.4, 130.5, 129.3, 128.7, 128.6, 128.1 (q, J_{CF} = 2.2 Hz, 2C), 127.6, 127.5, 127.0, 124.7, 100.9, 68.7 (q, J_{CF} = 27.5 Hz), 61.8, 14.3 ppm.

^{19}F NMR (470 MHz, CDCl$_3$) δ -73.16 (s, 3F) ppm.

FT-IR: ṽ = 3342, 1706, 1638, 1462, 1449, 1392, 1372, 1273, 1204, 1170, 1091, 1044, 1028, 1001 cm^{-1}.

HRMS (ESI): calcd. for $[M+H]^+$ $C_{21}H_{19}F_3NO_2$ = 374.1362; found 374.1365.

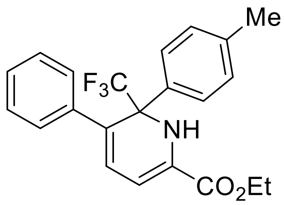

Ethyl 5-phenyl-6-(*p*-tolyl)-6-(trifluoromethyl)-1,6-dihydropyridine-2-carboxylate (4b)

Prepared according to general procedure **A**. The product was isolated as yellow amorphous solid (36 mg, 0.09 mmol, 64%) (eluent: 1% EtOAc in *n*-pentane).

^{1}H NMR (400 MHz, CDCl$_3$) δ 7.48 (d, *J* = 7.9 Hz, 2H), 7.21 – 7.12 (m, 3H), 7.09 (t, *J* = 7.4 Hz, 2H), 6.77 (d, *J* = 7.2 Hz, 2H), 6.33 (d, *J* = 6.5 Hz, 1H), 5.98 (dd, *J* = 6.5, 1.8 Hz, 1H), 5.02 (s, 1H), 4.28 (q, *J* = 7.1 Hz, 2H), 2.38 (s, 3H), 1.33 (t, *J* = 7.1 Hz, 3H) ppm.

^{13}C NMR (101 MHz, $CDCl_3$) δ 163.5, 139.5, 138.6, 137.9, 130.5, 129.4, 129.3 (d, J_{CF} = 3.5 Hz), 128.0 (q, J_{CF} = 2.2 Hz, 2C), 127.6, 127.5 (d, J_{CF} = 6.5 Hz), 127.3, 124.4, 100.9, 68.5 (q, J_{CF} = 27.4 Hz), 61.7, 21.2, 14.3 ppm.

^{19}F NMR (377 MHz, $CDCl_3$) δ -73.09 (s, 3F) ppm.

FT-IR: ṽ = 3353, 1704, 1638, 1496, 1443, 1375, 1274, 1254, 1171, 1160, 1149, 1079, 1037, 1005 cm^{-1}.

HRMS (ESI): calcd for $[M+H]^+$ $C_{22}H_{21}F_3NO_2$ = 388.1518; found 388.1521.

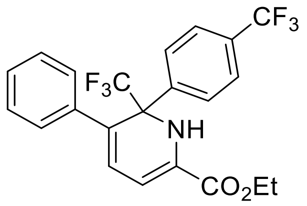

Ethyl 5-phenyl-6-(trifluoromethyl)-6-(4-(trifluoromethyl) phenyl)-1,6-dihydropyridine-2-carboxylate (4c)

Prepared according to the general procedure **A**. The product was isolated as yellow amorphous solid (39 mg, 0.09 mmol, 61%) (eluent: 1% EtOAc in *n*-pentane).

^{1}H NMR (500 MHz, $CDCl_3$) δ 7.73 (d, *J* = 8.3 Hz, 2H), 7.64 (d, *J* = 8.5 Hz, 2H), 7.22 – 7.15 (m, 1H), 7.11 (t, *J* = 7.5 Hz, 2H), 6.76 (d, *J* = 7.3 Hz, 2H), 6.38 (d, *J* = 6.5 Hz, 1H), 6.03 (dd, *J* = 6.4, 1.8 Hz, 1H), 5.10 (s, 1H), 4.29 (q, *J* = 7.1 Hz, 2H), 1.34 (t, *J* = 7.1 Hz, 3H) ppm.

^{13}C NMR (126 MHz, $CDCl_3$) δ 163.4, 144.1, 138.8, 130.8 (q, J_{CF} = 32.6 Hz), 130.6, 129.2, 128.7, 128.5 (q, J_{CF} = 2.4 Hz,), 128.2, 127.8 (d, J_{CF} = 1.9 Hz), 127.0 (d, J_{CF} = 54.4 Hz), 125.5 (q, J_{CF} = 3.8 Hz), 124.7 (d, J_{CF} = 73.1 Hz), 122.8, 101.4, 68.5 (q, J_{CF} = 28.2, 28.6 Hz), 62.0, 14.3 ppm.

^{19}F NMR (470 MHz, $CDCl_3$) -62.73 (s, 3F), -72.99 (s, 3F) ppm.

FT-IR: ṽ = 3320, 1695, 1637, 1497, 1414, 1395, 1379, 1330, 1318, 1282, 1205, 1170, 1118, 1001 cm^{-1}.

HRMS (ESI): calcd. for $[M+H]^+$ $C_{22}H_{18}F_6NO_2$ = 442.1236; found 442.1232.

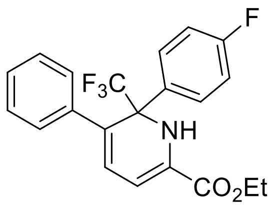

Ethyl 6-(4-fluorophenyl)-5-phenyl-6-(trifluoromethyl)-1,6-dihydropyridine-2-carboxylate (4d)

Prepared according to general procedure **A**. The product was isolated as yellow amorphous solid (50 mg, 0.13 mmol, 87%) (eluent: 1% EtOAc in *n*-pentane).

^{1}H NMR (500 MHz, $CDCl_3$) δ 7.58 (dd, *J* = 8.7, 5.2 Hz, 2H), 7.22 – 7.15 (m, 1H), 7.13 – 7.03 (m, 4H), 6.75 (d, *J* = 7.4 Hz, 2H), 6.34 (d, *J* = 6.5 Hz, 1H), 6.00 (dd, *J* = 6.5, 1.8 Hz, 1H), 5.06 (s, 1H), 4.29 (q, *J* = 7.1 Hz, 2H), 1.34 (t, *J* = 7.2 Hz, 3H) ppm.

^{13}C NMR (126 MHz, $CDCl_3$) δ 162.6 (d, J_{CF} = 249.0 Hz), 163.5, 139.2, 136.6 (d, J_{CF} = 3.5 Hz), 130.5, 130.1 (dd, J_{CF} = 8.3, 2.4 Hz), 129.3, 129.1, 127.7, 127.6, 126.9, 124.6, 115.5 (d, J_{CF} = 21.4 Hz), 101.1, 68.2 (q, J_{CF} = 27.6 Hz), 61.9, 14.3 ppm.

^{19}F NMR (470 MHz, $CDCl_3$) δ -73.41 (s, 3F), -112.99 (s, 1F) ppm.

FT-IR: ṽ = 3356, 1701, 1637, 1595, 1498, 1473, 1442, 1376, 1275, 1258, 1242, 1170, 1129, 1103 cm^{-1}.

HRMS (ESI): calcd. for $[M+H]^+$ $C_{21}H_{18}F_4NO_2$ = 392.1268; found 392.1268.

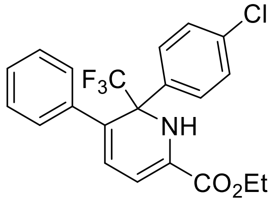

Ethyl 6-(4-chlorophenyl) -5-phenyl-6-(trifluoromethyl)-1,6-dihydropyridine-2-carboxylate (4e)

Prepared according to general procedure **A**. The product was isolated as yellow amorphous solid (48 mg, 0.12 mmol, 81%) (eluent: 1% EtOAc in *n*-pentane).

^{1}H NMR (500 MHz, $CDCl_3$) δ 7.53 (d, *J* = 8.4 Hz, 2H), 7.38 – 7.33 (m, 2H), 7.21 – 7.15 (m, 1H), 7.11 (t, *J* = 7.5 Hz, 2H), 6.77 (d, *J* = 7.3 Hz, 2H), 6.35 (d, *J* = 6.5 Hz, 1H), 6.00 (dd, *J* = 6.5, 1.8 Hz, 1H), 5.04 (s, 1H), 4.29 (q, *J* = 7.1 Hz, 2H), 1.34 (t, *J* = 7.1 Hz, 3H) ppm.

^{13}C NMR (126 MHz, CDCl$_3$) δ 163.4, 139.1 (d, J_{CF} = 8.8 Hz), 134.8, 130.5, 129.5 (q, J_{CF} = 2.4 Hz), 129.3, 128.9, 128.8, 127.9, 127.8, 127.7, 126.8, 124.5, 101.2, 68.3 (q, J_{CF} = 27.9 Hz), 61.9, 14.3 ppm.

^{19}F NMR (470 MHz, CDCl$_3$) δ -73.20 (s, 3F) ppm.

FT-IR: ṽ = 3399, 3335, 1702, 1638, 1595, 1493, 1443, 1393, 1373, 1283, 1256, 1156, 1034, 1013 cm^{-1}.

HRMS (ESI): calcd. for [M+H]$^+$ $C_{21}H_{18}{}^{35}ClF_3NO_2$ = 408.0972; found 408.0972; calcd. for [M+H]$^+$ $C_{21}H_{18}{}^{37}ClF_3NO_2$ = 410.0943; found 410.0942.

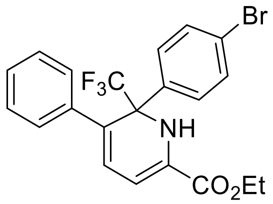

Ethyl 6-(4-bromophenyl)-5-phenyl-6-(trifluoromethyl)-1,6-dihydropyridine-2-carboxylate (4f)

Prepared according to general procedure **A**. The product was isolated as yellow amorphous solid (46 mg, 0.10 mmol, 70%) (eluent: 1% EtOAc in *n*-pentane).

^{1}H NMR (500 MHz, CDCl$_3$) δ 7.54 – 7.43 (m, 4H), 7.22 – 7.15 (m, 1H), 7.12 (t, *J* = 7.5 Hz, 2H), 6.77 (d, *J* = 7.2 Hz, 2H), 6.35 (d, *J* = 6.5 Hz, 1H), 6.00 (dd, *J* = 6.4, 1.8 Hz, 1H), 5.05 (s, 1H), 4.29 (q, *J* = 7.1 Hz, 2H), 1.34 (t, *J* = 7.2 Hz, 3H) ppm.

^{13}C NMR (126 MHz, CDCl$_3$) δ 163.4, 139.3 (d, J_{CF} = 75.3 Hz), 131.7, 130.5, 129.8 (q, J_{CF} = 2.3 Hz), 129.3, 128.8, 127.9, 127.8, 127.7, 126.8, 124.5, 123.1, 101.2, 68.4 (q, J_{CF} = 27.7 Hz), 61.9, 14.3 ppm.

^{19}F NMR (470 MHz, CDCl$_3$) δ -73.13 (s, 3F) ppm.

FT-IR: ṽ = 3399, 3335, 1702, 1638, 1595, 1493, 1443, 1393, 1373, 1283, 1156, 1095, 1077, 1013 cm^{-1}.

HRMS (ESI): calcd. for [M+H]$^+$ $C_{21}H_{18}{}^{79}BrF_3NO_2$ = 452.0467; found 452.0464; calcd. for [M+H]$^+$ $C_{21}H_{18}{}^{81}BrF_3NO_2$ = 454.0447; found 454.0443.

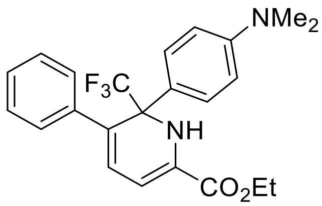

Ethyl 6-(4-(dimethylamino) phenyl)-5-phenyl-6-(trifluoromethyl)-1,6-dihydropyridine-2-carboxylate (4g)

Prepared according to general procedure **A**. The product was isolated as orange amorphous solid (26 mg, 0.06 mmol, 43%) (eluent: 20% EtOAc in *n*-pentane).

^{1}H NMR (500 MHz, $CDCl_3$) δ 7.43 (d, *J* = 8.5 Hz, 2H), 7.16 (t, *J* = 7.3 Hz, 1H), 7.10 (t, *J* = 7.4 Hz, 2H), 6.80 (d, *J* = 7.2 Hz, 2H), 6.73 (bs, 2H), 6.31 (d, *J* = 6.5 Hz, 1H), 5.96 (dd, *J* = 6.4, 1.8 Hz, 1H), 4.99 (s, 1H), 4.27 (q, *J* = 7.1 Hz, 2H), 3.00 (s, 6H), 1.33 (t, *J* = 7.1 Hz, 3H) ppm.

^{13}C NMR (126 MHz, $CDCl_3$) δ 163.6, 139.8, 130.4, 129.8, 129.3, 128.9, 127.6, 127.3, 127.1, 127.1, 124.7, 112.0, 111.0, 100.7, 68.3 (q, J_{CF} = 27.4 Hz), 61.7, 40.6, 14.3 ppm.

^{19}F NMR (470 MHz, $CDCl_3$) δ -73.02 (s, 3F) ppm.

FT-IR: ṽ = 3348, 1700, 1637, 1609, 1525, 1484, 1448, 1370, 1281, 1258, 1217, 1099, 1040, 1006 cm^{-1}.

HRMS (ESI): calcd. for $[M+H]^+$ $C_{23}H_{24}F_3N_2O_2$ = 417.1784; found 417.1781.

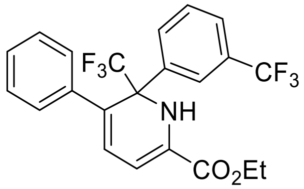

Ethyl 5-phenyl-6-(trifluoromethyl)-6-(3-(trifluoromethyl) phenyl)-1,6-dihydropyridine-2-carboxylate (4h)

Prepared according to general procedure **A**. The product was isolated as yellow amorphous solid (60 mg, 0.14 mmol, 93%) (eluent: 1% EtOAc in *n*-pentane).

^{1}H NMR (400 MHz, CDCl$_3$) δ 7.86 (s, 1H), 7.76 (d, *J* = 8.0 Hz, 1H), 7.62 (d, *J* = 7.8 Hz, 1H), 7.49 (t, *J* = 7.9 Hz, 1H), 7.22 – 7.14 (m, 1H), 7.10 (t, *J* = 7.4 Hz, 2H), 6.72 (d, *J* = 7.3 Hz, 2H), 6.37 (d, *J* = 6.5 Hz, 1H), 6.04 (dd, *J* = 6.5, 1.8 Hz, 1H), 5.12 (s, 1H), 4.31 (q, *J* = 7.1 Hz, 2H), 1.35 (t, *J* = 7.1 Hz, 3H) ppm.

^{13}C NMR (101 MHz, CDCl$_3$) δ 163.4, 140.1 (d, J_{CF} = 257.1 Hz), 131.4, 131.0 (d, J_{CF} = 32.5 Hz), 130.7, 129.3, 129.1, 128.7, 128.2, 127.8, 127.8, 127.2, 125.5 (q, J_{CF} = 3.6 Hz), 125.3, 124.3, 122.6, 101.5, 68.4 (q, J_{CF} = 28.0 Hz), 62.0, 14.3 ppm.

^{19}F NMR (377 MHz, CDCl$_3$) δ -62.68 (s, 3F), -73.45 (s, 3F) ppm.

FT-IR: ṽ = 3339, 1702, 1643, 1486, 1441, 1392, 1375, 1332, 1285, 1239, 1151, 1099, 1072, 1001 cm^{-1}.

HRMS (ESI): calcd. for $[M+H]^+$ $C_{22}H_{18}F_6NO_2$ = 442.1236; found 442.1233.

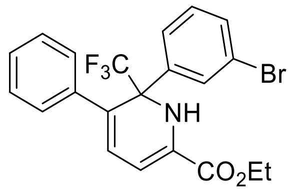

Ethyl 6-(3-bromophenyl) -5-phenyl-6-(trifluoromethyl) -1,6-dihydropyridine-2-carboxylate (4i)

Prepared according to general procedure **A**. The product was isolated as yellow amorphous solid (58 mg, 0.13 mmol, 88%) (eluent: 1% EtOAc in *n*-pentane).

^{1}H NMR (500 MHz, CDCl$_3$) 7.86 (s, 1H), 7.57 (t, *J* = 7.7 Hz, 2H), 7.36 – 7.29 (m, 1H), 7.28 – 7.23 (m, 1H), 7.19 (t, *J* = 7.5 Hz, 2H), 6.84 (d, *J* = 7.3 Hz, 2H), 6.43 (d, *J* = 6.5 Hz, 1H), 6.08 (dd, *J* = 6.4, 1.8 Hz, 1H), 5.15 (s, 1H), 4.37 (q, *J* = 7.1 Hz, 2H), 1.42 (t, *J* = 7.1 Hz, 3H) ppm.

^{13}C NMR (126 MHz, CDCl$_3$) δ 163.4, 142.6, 138.9, 131.9, 131.5 (d, J_{CF} = 2.4 Hz), 130.5, 130.0, 129.3, 128.8, 128.0, 127.8, 127.7, 126.5 (d, J_{CF} = 2.3 Hz), 124.4, 122.9, 101.2, 68.4 (q, J_{CF} = 27.7 Hz), 61.9, 14.3 ppm.

^{19}F NMR (470 MHz, CDCl$_3$) δ -73.08 (s, 3F) ppm.

FT-IR: ṽ = 3336, 1688, 1638, 1566, 1496, 1394, 1377, 1274, 1257, 1152, 1098, 1078, 1040, 1015 cm^{-1}.

HRMS (ESI): calcd. for $[M+H]^+$ $C_{21}H_{18}{}^{79}BrF_3NO_2$ = 452.0467; found 452.0465, calcd. for $[M+H]^+$ $C_{21}H_{18}{}^{81}BrF_3NO_2$ = 454.0447; found 454.0443.

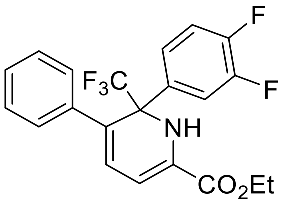

Ethyl 6-(3,4-difluorophenyl)-5-phenyl-6-(trifluoromethyl)-1,6-dihydropyridine-2-carboxylate (4j)

Prepared according to general procedure **A**. The product was isolated as yellow amorphous solid (30 mg, 0.07 mmol, 50%) (eluent: 1% EtOAc in *n*-pentane).

^{1}H NMR (600 MHz, $CDCl_3$) δ 7.44 (m, *J* = 11.8, 7.4, 2.4 Hz, 1H), 7.32 (d, *J* = 8.6 Hz, 1H), 7.22 – 7.09 (m, 4H), 6.77 (d, *J* = 7.3 Hz, 2H), 6.35 (d, *J* = 6.5 Hz, 1H), 6.01 (dd, *J* = 6.5, 1.8 Hz, 1H), 5.07 (s, 1H), 4.30 (q, *J* = 7.1 Hz, 2H), 1.35 (t, *J* = 7.1 Hz, 3H) ppm.

^{13}C NMR (151 MHz, $CDCl_3$) δ 163.4, 150.2 (dd, J_{CF} = 223.7, 12.6 Hz), 150.2 (dd, J_{CF} = 277.0, 12.6 Hz), 138.8, 137.5 (t, J_{CF} = 4.2 Hz), 130.5, 129.2, 128.7, 128.0, 127.9, 126.5, 124.6, 124.3, 117.9 (d, J_{CF} = 19.5 Hz), 117.3 (d, J_{CF} = 17.2 Hz), 101.4, 68.1 (q, J_{CF} = 27.7 Hz), 62.0, 14.3 ppm.

^{19}F NMR (470 MHz, $CDCl_3$) δ -73.39 (s, 3F), -135.89 – -136.10 (m, 1F), -136.99 – -137.19 (m, 1F) ppm.

FT-IR: ṽ = 3330, 1696, 1639, 1515, 1494, 1443, 1423, 1277, 1214, 1167, 1070, 1050, 1035, 1001 cm^{-1}.

HRMS (ESI): calcd. for $[M+H]^+$ $C_{21}H_{17}F_5NO_2$ = 410.1174; found 410.1172.

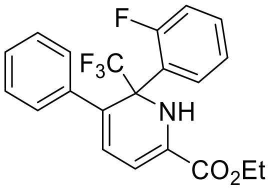

Ethyl 6-(2-fluorophenyl)-5-phenyl-6-(trifluoromethyl)-1,6-dihydropyridine-2-carboxylate (4k)

Prepared according to general procedure **A**. The product was isolated as yellow amorphous solid (18 mg, 0.05 mmol, 32%) (eluent: 1% EtOAc in *n*-pentane).

^{1}H NMR (500 MHz, CDCl$_3$) δ 7.45 – 7.32 (m, 2H), 7.19 – 7.02 (m, 5H), 6.92 (d, *J* = 7.3 Hz, 2H), 6.40 (d, *J* = 6.6 Hz, 1H), 6.00 (dd, *J* = 6.6, 1.9 Hz, 1H), 4.99 (s, 1H), 4.28 (q, *J* = 7.1 Hz, 2H), 1.34 (t, *J* = 7.1 Hz, 3H) ppm.

^{13}C NMR (126 MHz, CDCl$_3$) δ 163.5, 162.2 (d, J_{CF} = 253.6 Hz), 139.1, 131.2 (d, J_{CF} = 9.1 Hz), 130.9, 128.9, 128.0, 127.8, 127.5, 127.2 (d, J_{CF} = 7.3 Hz), 126.5, 124.2, 123.6 (d, J_{CF} = 3.9 Hz), 117.7, 117.5, 101.2, 67.1 (q, J_{CF} = 28.3 Hz), 61.8, 14.3 ppm.

^{19}F NMR (470 MHz, CDCl$_3$) δ -74.00 (s, 3F), -106.74 (s, 1F) ppm.

FT-IR: ṽ = 3164, 3002, 1443, 1375, 1038, 918 cm^{-1}.

HRMS (ESI): calcd for $[M+H]^+$. $C_{21}H_{18}F_4NO_2$ = 392.1268; found 392.1267.

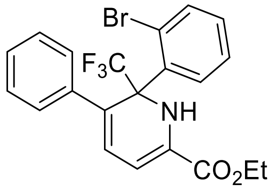

Ethyl 6-(2-bromophenyl)-5-phenyl-6-(trifluoromethyl)-1,6-dihydropyridine-2-carboxylate (4l)

Following the general procedure, the intermediate acyclic imine was isolated as the major product. To obtain the desired cyclized product, the imine was dissolved in toluene and heated at 110 °C (oil bath) for 72 h. The cyclized product was isolated as yellow amorphous solid (9.2 mg, 0.02 mmol, 20%) (eluent: 1% EtOAc in *n*-pentane).

^{1}H NMR (500 MHz, CDCl$_3$) δ 7.73 (dd, *J* = 7.8, 1.5 Hz, 1H), 7.45 – 7.41 (m, 1H), 7.29 – 7.24 (m, 2H), 7.21 (td, *J* = 7.6, 1.6 Hz, 1H), 7.14 – 7.09 (m, 1H), 7.05 (t, *J* = 7.5 Hz, 2H), 6.84 – 6.76 (m, 2H), 6.40 (d, *J* = 6.7 Hz, 1H), 6.03 (dd, *J* = 6.7, 1.9 Hz, 1H), 4.67 (s, 1H), 4.30 (qt, *J* = 7.4, 3.7 Hz, 2H), 1.35 (t, *J* = 7.1 Hz, 3H) ppm.

^{13}C NMR (126 MHz, CDCl$_3$) δ 163.5, 139.2, 136.9, 136.6, 131.3, 130.3, 129.7, 129.0, 129.0, 128.9, 127.7, 127.3, 126.9, 126.7, 124.5, 101.1, 70.2 (d, J_{CF} = 27.8 Hz), 61.8, 14.3 ppm.

^{19}F NMR (470 MHz, CDCl$_3$) δ -54.22 (s, 3F) ppm.

FT-IR: ṽ = 3356, 1701, 1637, 1595, 1498, 1442, 1376, 1275, 1196, 1170, 1150, 1129, 1103, 1027 cm^{-1}.

HRMS (ESI): calcd for [M+H] $^+$. $C_{21}H_{18}{}^{79}BrF_3NO_2$ = 452.0468; found 452.0464; calcd. for [M+H] $^+$ $C_{21}H_{18}{}^{81}BrF_3NO_2$ = 454.0447; found 454.0443.

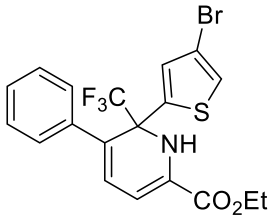

Ethyl 6-(4-bromothiophen-2-yl)-5-phenyl-6-(trifluoromethyl)-1,6-dihydropyridine-2-carboxylate (4m)

Prepared according to general procedure **A**. The product was isolated as yellow amorphous solid (40 mg, 0.09 mmol, 60%) (eluent: 1% EtOAc in *n*-pentane).

^{1}H NMR (500 MHz, CDCl$_3$) δ 7.33 (d, *J* = 1.4 Hz, 1H), 7.27 – 7.22 (m, 1H), 7.19 (t, *J* = 7.4 Hz, 2H), 6.86 (d, *J* = 7.3 Hz, 3H), 6.29 (d, *J* = 6.4 Hz, 1H), 6.08 (dd, *J* = 6.4, 1.7 Hz, 1H), 5.36 (s, 1H), 4.31 (q, *J* = 7.1 Hz, 2H), 1.35 (t, *J* = 7.1 Hz, 3H) ppm.

^{13}C NMR (126 MHz, CDCl$_3$) δ 163.2, 145.4, 138.3, 129.9 (d, J_{CF} = 107.6 Hz), 129.3, 129.0 (q, J_{CF} = 2.7 Hz), 128.0, 127.8, 127.7, 126.1, 124.9, 123.8, 109.9, 102.6, 66.2 (q, J_{CF} = 29.1 Hz), 62.0, 14.3 ppm.

^{19}F NMR (470 MHz, CDCl$_3$) δ -75.56 (s, 3F) ppm.

FT-IR: ṽ = 3357, 3115, 1685, 1629, 1524, 1487, 1393, 1337, 1285, 1253, 1207, 1149, 1029, 1012 cm^{-1}.

HRMS (ESI): calcd. for [M+H] $^+$ $C_{19}H_{16}{}^{79}BrF_3NO_2S$ = 458.0032; found 458.0028; calcd. for [M+H] $^+$ $C_{19}H_{16}{}^{81}BrF_3NO_2S$ = 460.0011; found 460.0006.

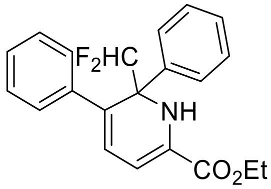

Ethyl 6-(difluoromethyl)-5,6-diphenyl-1,6-dihydropyridine-2-carboxylate (4n)

Prepared according to general procedure **A**. The product was isolated as yellow amorphous solid (25 mg, 0.07 mmol, 48%) (eluent: 1% EtOAc in *n*-pentane).

^{1}H NMR (500 MHz, CDCl$_3$) δ 7.58 – 7.49 (m, 2H), 7.41 – 7.31 (m, 3H), 7.22 – 7.12 (m, 3H), 6.92 – 6.88 (m, 2H), 6.38 – 6.13 (m, 2H), 5.95 (dd, *J* = 6.4, 1.7 Hz, 1H), 5.10 (s, 1H), 4.27 (qd, *J* = 7.1, 4.7 Hz, 2H), 1.33 (t, *J* = 7.1 Hz, 3H) ppm.

^{13}C NMR (126 MHz, CDCl$_3$) δ 163.7, 141.8, 138.7, 131.0, 130.8, 128.8, 128.4 (d, J_{CF} = 76.6 Hz), 128.1 (d, J_{CF} = 87.2 Hz), 127.4, 125.9, 116.5, 114.5, 112.5, 101.0, 65.9 (t, J_{CF} = 18.9 Hz), 61.7, 14.3 ppm.

^{19}F NMR (470 MHz, CDCl$_3$) δ -125.61 (dd, J_{FF} = 276.8, 54.7 Hz, 1F), -134.70 (dd, J_{FF} = 276.8, 54.6 Hz, 1F) ppm.

FT-IR: ṽ = 3409, 1704, 1676, 1637, 1492, 1447, 1372, 1278, 1151, 1111, 1071, 1032, 953, 904, 861 cm^{-1}.

HRMS (ESI): calcd for [M+H]$^{+}$. $C_{21}H_{20}F_2NO_2$ = 356.1457; found 356.1459.

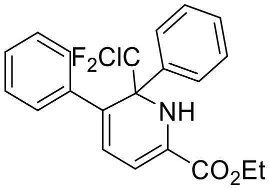

Ethyl 6-(chlorodifluoromethyl)-5,6-diphenyl-1,6-dihydropyridine-2-carboxylate (4o)

Prepared according to general procedure **A**. The product was isolated as yellow amorphous solid (55.3 mg, 0.14 mmol, 97%) (eluent: 1% EtOAc in *n*-pentane).

^{1}H NMR (500 MHz, CDCl$_3$) δ 7.63 – 7.58 (m, 2H), 7.40 – 7.34 (m, 3H), 7.16 – 7.10 (m, 1H), 7.05 (t, *J* = 7.7 Hz, 2H), 6.58 (d, *J* = 7.0 Hz, 2H), 6.27 (d, *J* = 6.5 Hz, 1H), 5.99 (dd, *J* = 6.5, 1.9 Hz, 1H), 5.27 (s, 1H), 4.32 (qd, *J* = 7.1, 2.2 Hz, 2H), 1.36 (t, *J* = 7.1 Hz, 3H) ppm.

^{13}C NMR (126 MHz, CDCl$_3$) δ 163.7, 141.7, 139.9, 130.8, 129.7, 128.7, 128.6, 128.3, 127.6 (d, J_{CF} = 78.0 Hz), 127.4, 125.8 (dd, J_{CF} = 7.1, 3.7 Hz), 119.5, 110.7, 100.5, 72.74 (t, J_{CF} = 23.2 Hz), 61.8, 14.4 ppm.

^{19}F NMR (470 MHz, CDCl$_3$ δ -55.51, -83.18 (dd, *J*FF = 163.1 Hz, 23.3 Hz, 1F), -60.84, -90.56 (dd, *J*FF = 162.9 Hz, 23.3 Hz, 1F) ppm.

FT-IR: ṽ = 3343, 3058, 1701, 1637, 1494, 1467, 1390, 1372, 1292, 1256, 1174, 1084, 1049, 1011 cm^{-1}.

HRMS (ESI): calcd. for [M+H]$^{+}$ $C_{21}H_{19}{}^{35}ClF_2NO_2$ = 390.1067; found .390.1068; calcd. for [M+H]$^{+}$ $C_{21}H_{19}{}^{37}ClF_2NO_2$ = 392.1037; found 392.1038.

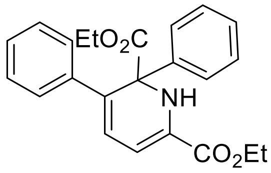

Diethyl 2,3-diphenyl-1,2-dihydropyridine-2,6-dicarboxylate (4p)

Prepared according to general procedure **A**. The product was isolated as orange amorphous solid (29.8 mg, 0.08 mmol, 54%) (eluent: 10% EtOAc in *n*-pentane).

^{1}H NMR (500 MHz, $CDCl_3$) δ 7.36 – 7.32 (m, 2H), 7.23 – 7.19 (m, 3H), 7.15 – 7.12 (m, 2H), 7.09 – 7.05 (m, 3H), 6.41 (d, *J* = 6.2 Hz, 1H), 6.25 (d, *J* = 6.2 Hz, 1H), 5.51 (s, 1H), 4.38 – 4.20 (m, 4H), 1.33 (t, *J* = 7.1 Hz, 3H), 1.23 (t, *J* = 7.1 Hz, 3H) ppm.

^{13}C NMR (126 MHz, $CDCl_3$) δ 174.7, 163.6, 140.5, 139.2, 133.5, 132.7, 129.2, 128.4, 128.2, 128.1, 127.5, 127.0, 124.4, 105.9, 69.5, 61.8, 61.5, 14.4, 14.3.

FT-IR: ṽ = 3391, 1731, 1703, 1624, 1447, 1390, 1370, 1275, 1261, 1227, 1155, 1104, 1018, 942 cm^{-1}.

HRMS (ESI): calcd. for $[M+H]^+$ $C_{23}H_{24}NO_4$ = 378.1700; found 378.1701.

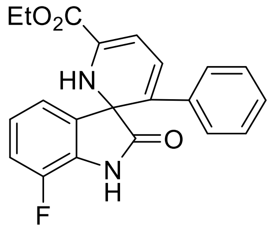

Ethyl 7-fluoro-2-oxo-3'-phenyl-1'H-spiro[indoline-3,2'-pyridine]-6'-carboxylate (4q)

Prepared according to general procedure **A**. The product was isolated as orange amorphous solid (24 mg, 0.07 mmol, 45%, inseparable mixture with the intermediate acyclic imine, 4:1 ratio of product vs. intermediate) after purification by column chromatography (eluent: 8% EtOAc in *n*-pentane) and preparative TLC (eluent: 15% EtOAc in *n*-pentane).

(Product is mixture with intermediate acyclic imine. Signals are reported for main product.)

^{1}H NMR (500 MHz, CDCl$_3$) δ 7.72 (s, 1H), 7.59 (dd, *J* = 8.1, 1.3 Hz, 2H), 7.48 – 7.40 (m, 3H), 7.12 (d, *J* = 3.9 Hz, 1H), 7.01 – 6.94 (m, 1H), 6.91 – 6.80 (m, 1H), 6.58 (d, *J* = 7.4 Hz, 1H), 6.37 (d, *J* = 3.9 Hz, 1H), 5.86 (s, 1H), 4.03 (qq, *J* = 10.8, 7.1 Hz, 1H), , 1.15 (t, *J* = 7.1 Hz, 3H) ppm.

^{13}C NMR (126 MHz, CDCl$_3$) δ 174.8, 160.8, 148.0, 146.1, 144.1, 131.5, 131.1, 129.7, 129.2, 129.1, 127.4, 123.1 (d, J_{CF} = 5.4 Hz), 119.6, 118.0 (d, J_{CF} = 3.2 Hz), 116.1 (d, J_{CF} = 17.3 Hz), 110.2, 60.2, 59.2 (d, J_{CF} = 3.5 Hz), 14.3 ppm.

^{19}F NMR (470 MHz, CDCl$_3$) δ -134.5 (dd, *J* = 9.9, 4.7 Hz, 1F) ppm.

FT-IR: ṽ = 3257, 1747, 1669, 1605, 1460, 1400, 1323, 1252, 1228, 1198, 1088, 1019, 908, 819 cm^{-1}.

HRMS (ESI): calcd for [M+H]$^+$. $C_{21}H_{18}FN_2O_3$ = 365.1296, found 365.1300.

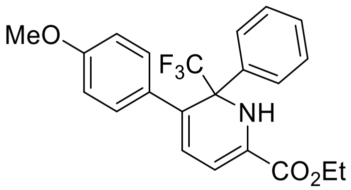

Ethyl 5-(4-methoxyphenyl)-6-phenyl-6-(trifluoromethyl)-1,6-dihydropyridine-2-carboxylate (5a)

Prepared according to general procedure **A**. The product was isolated as yellow amorphous solid (50 mg, 0.12 mmol, 94%) (eluent: 2% EtOAc in *n*-pentane).

^{1}H NMR (500 MHz, CDCl$_3$) δ 7.61 (d, *J* = 8.4 Hz, 2H), 7.45 – 7.33 (m, 3H), 6.75 – 6.65 (m, 2H), 6.65 – 6.57 (m, 2H), 6.32 (d, *J* = 6.5 Hz, 1H), 5.99 (dd, *J* = 6.5, 1.9 Hz, 1H), 5.08 – 4.90 (m, 1H), 4.28 (q, *J* = 7.1 Hz, 2H), 3.72 (s, 3H), 1.33 (t, *J* = 7.1 Hz, 3H). ppm.

^{13}C NMR (126 MHz, CDCl$_3$) δ 163.4, 158.9, 140.7, 131.7, 130.3, 130.1, 128.8, 128.5, 128.5, 128.0 (q, J_{CF} = 2.3 Hz), 126.9, 124.6, 112.9, 101.1, 68.5 (q, J_{CF} = 27.3 Hz), 61.6, 55.1, 14.2 ppm.

^{19}F NMR (470 MHz, CDCl$_3$) δ -72.98 (s, 3F) ppm.

FT-IR: ṽ = 3339, 1706, 1638, 1607, 1572, 1493, 1443, 1388, 1276, 1258, 1242, 1207, 1069, 1006 cm^{-1}.

HRMS (ESI): calcd for [M+H]$^+$. $C_{22}H_{21}F_3NO_3$ = 404.1468, found 404.1465.

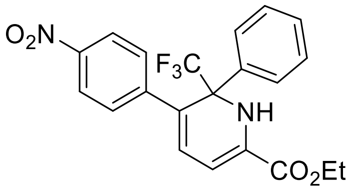

Ethyl 5-(4-nitrophenyl) -6-phenyl-6-(trifluoromethyl) -1,6-dihydropyridine-2-carboxylate (5b)

Prepared according to general procedure **A**. The product was isolated as orange amorphous solid (44 mg, 0.11 mmol, 83%) (eluent: 2% EtOAc in *n*-pentane).

^{1}H NMR (500 MHz, CDCl$_3$) δ 8.02 – 7.82 (m, 2H), 7.59 (d, *J* = 8.1 Hz, 2H), 7.47 – 7.35 (m, 3H), 6.85 (d, *J* = 8.9 Hz, 2H), 6.44 (d, *J* = 6.6 Hz, 1H), 6.00 (dd, *J* = 6.6, 1.9 Hz, 1H), 5.16 (s, 1H), 4.31 (q, *J* = 7.1 Hz, 2H), 1.35 (t, *J* = 7.1 Hz, 3H) ppm.

^{13}C NMR (126 MHz, CDCl$_3$) δ 163.1, 146.9, 146.2, 140.2, 131.7, 130.0, 129.6, 129.2, 128.9, 128.1 (d, J_{CF} = 2.3 Hz), 126.9, 126.3, 124.6, 122.9, 100.1, 68.6 (d, J_{CF} = 27.8 Hz), 62.1, 14.3 ppm.

^{19}F NMR (470 MHz, CDCl$_3$) δ -73.90 (s, 3F) ppm.

FT-IR: ṽ = 3343, 1698, 1633, 1593, 1515, 1493, 1452, 1280, 1212, 1151, 1106, 1072, 1046, 1030 cm^{-1}.

HRMS (ESI): calcd. for $[M+H]^+$ $C_{21}H_{18}F_3N_2O_4$ m/z 419.1213; found 419.1212.

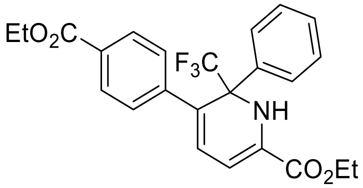

Ethyl 5-(4-(ethoxycarbonyl) phenyl) -6-phenyl-6-(trifluoromethyl) -1,6-dihydropyridine-2-carboxylate (5c)

Prepared according to general procedure **A**. The product was isolated as a yellow amorphous solid (30 mg, 0.07 mmol, 49%) (eluent: 2% EtOAc in *n*-pentane).

^{1}H NMR (500 MHz, CDCl$_3$) δ 7.74 (d, *J* = 8.5 Hz, 2H), 7.59 (d, *J* = 7.3 Hz, 2H), 7.44 – 7.37 (m, 3H), 6.78 (d, *J* = 8.5 Hz, 2H), 6.39 (d, *J* = 6.5 Hz, 1H), 5.99 (dd, *J* = 6.5, 1.9 Hz, 1H), 5.09 (s, 1H), 4.30 (dq, *J* = 10.8, 7.1 Hz, 4H), 1.34 (td, *J* = 7.1, 1.2 Hz, 6H) ppm.

^{13}C NMR (126 MHz, $CDCl_3$) δ 166.4, 163.3, 144.0, 140.5, 131.1, 129.3, 129.2, 128.9, 128.9, 128.7, 128.5, 128.2 (d, J_{CF} = 2.2 Hz), 127.9, 125.8 (d, J_{CF} = 291.6 Hz), 100.6, 68.6 (q, J_{CF} = 27.8 Hz)., 61.9, 61.0, 14.4, 14.3 ppm.

^{19}F NMR (470 MHz, $CDCl_3$) δ -73.61 (s, 3F) ppm.

FT-IR: ṽ = 3329, 1714, 1704, 1631, 1607, 1491, 1451, 1377, 1283, 1246, 1205, 1158, 1108, 1026 cm^{-1}.

HRMS (ESI): calcd. for $[M+H]^+$ $C_{24}H_{23}F_3NO_4$ = 446.1574; found 446.1574.

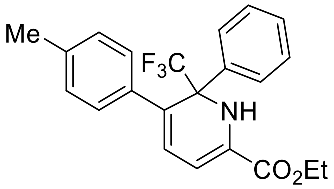

Ethyl 6-phenyl-5-(p-tolyl) -6-(trifluoromethyl) -1,6-dihydropyridine-2-carboxylate (5d)

Prepared according to general procedure **A**. The product was isolated as yellow amorphous solid (46 mg, 0.12 mmol, 86%) (eluent: 1% EtOAc in *n*-pentane).

^{1}H NMR (500 MHz, $CDCl_3$) δ 7.62 (d, *J* = 7.5 Hz, 2H), 7.43 – 7.32 (m, 3H), 6.90 (d, *J* = 8.0 Hz, 2H), 6.64 (d, *J* = 8.2 Hz, 2H), 6.34 (d, *J* = 6.5 Hz, 1H), 6.00 (dd, *J* = 6.5, 1.8 Hz, 1H), 5.05 (s, 1H), 4.29 (q, *J* = 7.1 Hz, 2H), 2.25 (s, 3H), 1.34 (t, *J* = 7.1 Hz, 3H) ppm.

^{13}C NMR (126 MHz, $CDCl_3$) δ 163.5, 140.8, 137.3, 136.5, 130.3, 129.3, 129.1, 128.6, 128.5, 128.4, 128.1 (d, J_{CF} = 2.3 Hz), 127.3, 125.9 (d, J_{CF} = 291.2 Hz), 101.1, 68.7 (q, J_{CF} = 27.4 Hz), 61.7, 21.2, 14.3 ppm.

^{19}F NMR (470 MHz, $CDCl_3$) δ -73.01 (s, 3F) ppm.

FT-IR: ṽ = 3353, 1704, 1637, 1501, 1477, 1449, 1379, 1255, 1204, 1191, 1178, 1147, 1118, 1022 cm^{-1}.

HRMS (ESI): calcd for $[M+H]^+$. $C_{22}H_{21}F_3NO_2$ = 388.1519; found 388.1521.

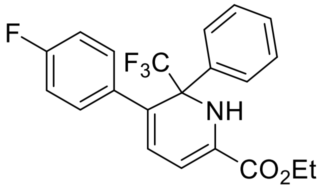

Ethyl 5-(4-fluorophenyl) -6-phenyl-6-(trifluoromethyl) -1,6-dihydropyridine-2-carboxylate (5e)

Prepared according to general procedure **A**. The product was isolated as yellow amorphous solid (49 mg, 0.13 mmol, 78%) (eluent: 1% EtOAc in *n*-pentane).

^{1}H NMR (500 MHz, $CDCl_3$) δ 7.59 (d, *J* = 7.5 Hz, 2H), 7.48 – 7.33 (m, 3H), 6.83 – 6.72 (m, 2H), 6.72 – 6.59 (m, 2H), 6.31 (d, *J* = 6.5 Hz, 1H), 5.98 (dd, *J* = 6.5, 1.8 Hz, 1H), 5.08 (s, 1H), 4.29 (q, *J* = 7.1 Hz, 2H), 1.34 (t, *J* = 7.1 Hz, 3H) ppm.

^{13}C NMR (126 MHz, $CDCl_3$) δ 163.4, 162.2 (d, *J*CF = 247.0 Hz), 140.6, 135.4, 135.3, 131.0 (d, J_{CF} = 8.0 Hz), 130.7, 128.8, 128.6, 128.1 (t, J_{CF} = 2.4 Hz), 127.8, 125.8 (d, J_{CF} = 291.4 Hz), 114.6 (d, J_{CF} = 21.3 Hz), 100.7, 68.6 (q, J_{CF} = 27.5 Hz), 61.8, 14.3 ppm.

^{19}F NMR (470 MHz, $CDCl_3$) δ -73.37 (s, 3F), -114.73 (s, 1F) ppm.

FT-IR: ṽ = 3344, 1697, 1633, 1594, 1505, 1394, 1375, 1283, 1254, 1219, 1175, 1162, 1069, 1016 cm^{-1}.

HRMS (ESI): calcd. for $[M+H]^+$ $C_{21}H_{18}F_4NO_2$ = 392.1268; found 392.1266.

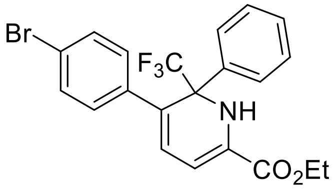

Ethyl 5-(4-bromophenyl) -6-phenyl-6-(trifluoromethyl) -1,6-dihydropyridine-2-carboxylate (5f)

Prepared according to general procedure **A**. The product was isolated as yellow amorphous solid (48 mg, 0.11 mmol, 78%) (eluent: 1% EtOAc in *n*-pentane).

^{1}H NMR (500 MHz, $CDCl_3$) δ 7.58 (d, *J* = 7.4 Hz, 2H), 7.39 (m, 3H), 7.22 – 7.16 (m, 2H), 6.62 – 6.50 (m, 2H), 6.32 (d, *J* = 6.5 Hz, 1H), 5.97 (dd, *J* = 6.5, 1.8 Hz, 1H), 5.07 (s, 1H), 4.29 (q, *J* = 7.1 Hz, 2H), 1.34 (t, *J* = 7.1 Hz, 3H) ppm.

^{13}C NMR (126 MHz, $CDCl_3$) 163.4, 140.5, 138.3, 130.9, 130.8, 128.9, 128.7, 128.1 (d, J_{CF} = 2.4 Hz), 128.0, 127.8, 127.0, 124.7, 121.7, 100.6, 68.5 (q, J_{CF} = 27.6 Hz), 61.9, 14.3 ppm.

^{19}F NMR (470 MHz, $CDCl_3$) δ -73.50 (s, 3F) ppm.

FT-IR: ṽ = 3340, 1712, 1639, 1503, 1484, 1449, 1378, 1203, 1176, 1158, 1148, 1108, 1018, 1010 cm^{-1}.

HRMS (ESI): calcd for $[M+H]^+$. $C_{21}H_{18}{}^{79}BrF_3NO_2$ = 452.0468; found 452.0465; calcd. for $[M+H]^+$ $C_{21}H_{18}{}^{81}BrF_3NO_2$ = 454.0447; found 454.0443.

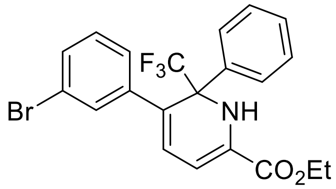

Ethyl 5-(3-bromophenyl)-6-phenyl-6-(trifluoromethyl)-1,6-dihydropyridine-2-carboxylate (5g)

Prepared according to general procedure **A**. The product was isolated as yellow amorphous solid (45 mg, 0.10 mmol, 87%) (eluent: 1% EtOAc in *n*-pentane).

^{1}H NMR (500 MHz, CDCl$_3$) δ 7.59 (d, J = 7.2 Hz, 2H), 7.46 – 7.34 (m, 3H), 7.28 (d, J = 1.0 Hz, 1H), 6.92 (t, J = 7.9 Hz, 1H), 6.83 (t, J = 1.9 Hz, 1H), 6.60 (d, J = 8.0 Hz, 1H), 6.33 (d, J = 6.5 Hz, 1H), 5.97 (dd, J = 6.5, 1.9 Hz, 1H), 5.09 (s, 1H), 4.29 (q, J = 7.1 Hz, 2H), 1.34 (t, J = 7.1 Hz, 3H) ppm.

^{13}C NMR (126 MHz, CDCl$_3$) δ 163.3, 141.4, 140.4, 132.3, 131.1, 130.4, 129.0 (d, J_{CF} = 15.7 Hz), 128.7, 128.3, 128.2 (q, J_{CF} = 2.3 Hz), 127.9, 127.5, 127.0, 124.7, 121.6, 100.5, 68.5 (q, J_{CF} = 27.7 Hz), 61.9, 14.3 ppm.

^{19}F NMR (470 MHz, CDCl$_3$) δ -73.78 (s, 3F) ppm.

FT-IR: ṽ = 3348, 1792, 1702, 1639, 1590, 1555, 1440, 1376, 1282, 1208, 1148, 1074, 1018, 1003 cm^{-1}.

HRMS (ESI): calcd. for $[M+H]^+$ $C_{21}H_{18}{}^{79}BrF_3NO_2$ = 452.0475; found 452.0467; calcd. for $[M+H]^+$ $C_{21}H_{18}{}^{81}BrF_3NO_2$ = 454.0447; found 454.0445.

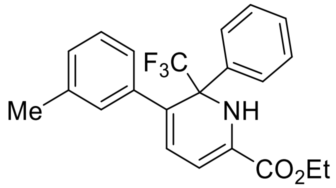

Ethyl 6-phenyl-5-(m-tolyl)-6-(trifluoromethyl)-1,6-dihydropyridine-2-carboxylate (5h)

Prepared according to general procedure **A**. The product was isolated as yellow amorphous solid (45 mg, 0.12 mmol, 83%) (eluent: 1% EtOAc in *n*-pentane).

^{1}H NMR (500 MHz, CDCl$_3$) δ 7.61 (d, *J* = 7.5 Hz, 2H), 7.44 – 7.35 (m, 3H), 6.98-6.93 (m, 2H), 6.53 (s, 1H), 6.49 (d, *J* = 6.7 Hz, 1H), 6.33 (d, *J* = 6.5 Hz, 1H), 5.99 (dd, *J* = 6.4, 1.8 Hz, 1H), 5.06 (s, 1H), 4.29 (q, *J* = 7.1 Hz, 2H), 2.16 (s, 3H), 1.34 (t, *J* = 7.1 Hz, 3H). ppm.

^{13}C NMR (126 MHz, CDCl$_3$) δ 163.5, 140.8, 139.3, 137.1, 130.5, 130.1, 129.5, 128.6, 129.5, 128.2, 127.4, 127.4, 127.0, 126.3, 124.7, 101.0, 68.7 (q, J_{CF} = 27.3 Hz), 61.8, 21.4, 14.3 ppm.

^{19}F NMR (470 MHz, CDCl$_3$) δ -73.31 (s, 3F) ppm.

FT-IR: ṽ = 3337, 1699, 1626, 1493, 1465, 1451, 1374, 1285, 1210, 1187, 1093, 1074, 1027, 1003 cm^{-1}.

HRMS (ESI): calcd for $[M+H]^+$. $C_{22}H_{21}F_3NO_2$ = 388.1519; found 388.1519.

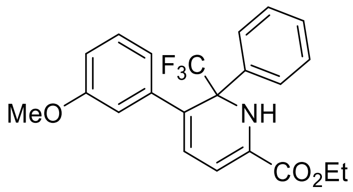

Ethyl 5-(3-methoxyphenyl) -6-phenyl-6-(trifluoromethyl) -1,6-dihydropyridine-2-carboxylate (5i)

Prepared according to general procedure **A**. The product was isolated as yellow amorphous solid (40 mg, 0.10 mmol, 75%) (eluent: 2% EtOAc in *n*-pentane).

^{1}H NMR (500 MHz, CDCl$_3$) δ 7.62 (d, *J* = 8.0 Hz, 2H), 7.45 – 7.33 (m, 3H), 7.01 (t, *J* = 8.0 Hz, 1H), 6.70 (ddd, *J* = 8.3, 2.6, 0.8 Hz, 1H), 6.42 (d, *J* = 7.8 Hz, 1H), 6.36 (d, *J* = 6.5 Hz, 1H), 6.24 – 6.13 (m, 1H), 5.98 (dd, *J* = 6.5, 1.9 Hz, 1H), 5.05 (s, 1H), 4.29 (q, *J* = 7.1 Hz, 2H), 3.52 (s, 3H), 1.34 (t, *J* = 7.1 Hz, 3H) ppm.

^{13}C NMR (126 MHz, CDCl$_3$) δ 163.5, 158.6, 141.0, 140.7, 130.6, 129.0, 128.9, 128.7, 128.6, 128.6, 128.2 (d, J_{CF} = 2.1 Hz), 127.6, 122.0, 114.3, 113.6, 100.7, 61.8, 55.0, 38.9, 14.3 ppm.

^{19}F NMR (470 MHz, CDCl$_3$) δ -73.47 (s, 3F) ppm.

FT-IR: ṽ = 3346, 2963, 1708, 1633, 1595, 1493, 1377, 1266, 1217, 1204, 1153, 1083, 1028, 1002, cm^{-1}.

HRMS (ESI): calcd. for $[M+H]^+$ $C_{22}H_{21}F_3NO_3$ = 404.1468; found 404.1467.

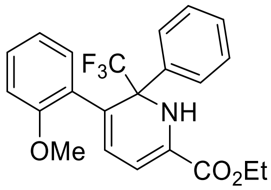

Ethyl 5-(2-methoxyphenyl)-6-phenyl-6-(trifluoromethyl)-1,6-dihydropyridine-2-carboxylate (5j)

Prepared according to general procedure **A**. The product was isolated as yellow amorphous solid (36 mg, 0.09 mmol, 57%) (eluent: 2% EtOAc in *n*-pentane).

^{1}H NMR (500 MHz, $CDCl_3$) δ 7.51 – 7.37 (m, 2H), 7.26 – 7.23 (m, 3H), 7.15 – 7.10 (m, 1H), 6.67 (td, *J* = 7.4, 1.1 Hz, 1H), 6.61 (d, *J* = 8.2 Hz, 2H), 6.15 (d, *J* = 6.4 Hz, 1H), 5.98 (dd, *J* = 6.4, 1.8 Hz, 1H), 5.10 (s, 1H), 4.29 (qd, *J* = 7.1, 1.1 Hz, 2H), 3.33 (s, 3H), 1.33 (t, *J* = 7.1 Hz, 3H) ppm.

^{13}C NMR (126 MHz, $CDCl_3$) δ 163.7, 157.4, 139.8, 131.8, 131.3, 129.0, 128.4, 128.1, 127.9 (d, J_{CF} = 2.5 Hz), 127.8, 127.5, 125.3, 125.2, 119.4, 110.3, 101.1, 68.0 (q, J_{CF} = 27.4 Hz), 61.7, 55.1, 14.4 ppm.

^{19}F NMR (470 MHz, $CDCl_3$) δ -75.55 (s, 3F) ppm.

FT-IR: ṽ = 3342, 1698, 1630, 1595, 1578, 1495, 1463, 1392, 1273, 1230, 1152, 1116, 1054, 1023 cm^{-1}.

HRMS (ESI): calcd. for $[M+H]^+ C_{22}H_{21}F_3NO_3$ = 404.1468; found 404.1466.

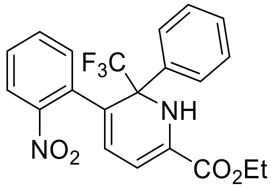

Ethyl 5-(2-nitrophenyl) -6-phenyl-6-(trifluoromethyl)-1,6-dihydropyridine-2-carboxylate (5k)

Prepared according to general procedure **A**. The product was isolated as yellow amorphous solid (47 mg, 0.11 mmol, 76%) (eluent: 2% EtOAc in *n*-pentane).

^{1}H NMR (500 MHz, $CDCl_3$) δ 7.55 (dd, *J* = 7.9, 1.6 Hz, 1H), 7.32 – 7.20 (m, 7H), 6.91 (s, 1H), 6.26 (d, *J* = 6.5 Hz, 1H), 5.99 (dd, *J* = 6.5, 1.8 Hz, 1H), 5.16 (s, 1H), 4.26 (qd, *J* = 7.1, 3.2 Hz, 2H), 1.30 (t, *J* = 7.1 Hz, 3H) ppm.

^{13}C NMR (126 MHz, CDCl$_3$) δ 163.4, 149.6, 137.7, 133.4, 133.1, 132.4, 131.6, 130.0, 128.7 (d, J_{CF} = 20.4 Hz), 128.5, 128.0 (d, J_{CF} = 2.5 Hz), 127.6, 125.2, 123.9, 121.9, 101.1, 67.7 (q, J_{CF} = 27.7 Hz), 62.0, 14.3 ppm.

^{19}F NMR (470 MHz, CDCl$_3$) δ -75.89 (s, 3F) ppm.

FT-IR: ṽ = 3337, 1701, 1634, 1570, 1523, 1451, 1373, 1341, 1275, 1209, 1173, 1145, 1065, 1024 cm^{-1}.

HRMS (ESI): calcd. for $[M+H]^+$ $C_{21}H_{18}F_3N_2O_4$ = 419.1213; found 419.1210.

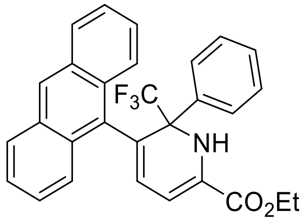

Ethyl 5-(anthracen-9-yl)-6-phenyl-6-(trifluoromethyl)-1,6-dihydropyridine-2-carboxylate (5l)

Prepared according to general procedure **A**. The product was isolated as yellow amorphous solid (40 mg, 0.09 mmol, 78%) (eluent: 1% EtOAc in *n*-pentane).

^{1}H NMR (500 MHz, CDCl$_3$) δ 8.43 – 8.31 (m, 2H), 8.01 (d, *J* = 8.2 Hz, 1H), 7.73 (d, *J* = 8.5 Hz, 1H), 7.59 – 7.44 (m, 2H), 7.27 (d, *J* = 9.5 Hz, 1H), 7.17 – 7.07 (m, 1H), 6.93 – 6.86 (m, 3H), 6.83 – 6.72 (m, 3H), 6.53 (d, *J* = 6.2 Hz, 1H), 6.27 (dd, *J* = 6.2, 1.8 Hz, 1H), 5.53 (s, 1H), 4.44 (qd, *J* = 7.2, 5.3 Hz, 2H), 1.45 (t, *J* = 7.2 Hz, 3H) ppm.

^{13}C NMR (126 MHz, CDCl$_3$) δ 163.9, 136.2, 133.0, 132.1, 131.5, 131.2, 130.7 (d, J_{CF} = 53.4 Hz), 128.6, 127.8, 127.8 (d, J_{CF} = 2.5 Hz), 127.6, 127.4, 127.4, 127.1 (d, J_{CF} = 2.8 Hz), 126.7, 125.5, 125.2, 124.7, 124.4 (d, J_{CF} = 26.5 Hz), 101.9, 67.1 (q, J_{CF} = 27.6 Hz) 62.1, 14.4 ppm.

^{19}F NMR (470 MHz, CDCl$_3$) δ -75.79 (s, 3F) ppm.

FT-IR: ṽ = 3337, 3048, 1697, 1624, 1487, 1460, 1391, 1369, 1302, 1278, 1155, 1095, 1059, 1006 cm^{-1}.

HRMS (ESI): calcd for $[M+H]^+$ $C_{29}H_{23}F_3NO_2$ = 474.1675; found 474.1670.

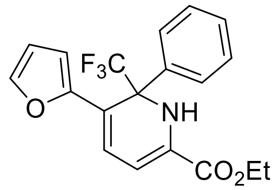

Ethyl 5-(furan-2-yl)-6-phenyl-6-(trifluoromethyl)-1,6-dihydropyridine-2-carboxylate (5m)

Prepared according to general procedure **A**. The product was isolated as yellow amorphous solid (49 mg, 0.14 mmol, 89%) (eluent: 1% EtOAc in *n*-pentane).

^{1}H NMR (500 MHz, $CDCl_3$) δ 7.72 (d, *J* = 8.1 Hz, 2H), 7.40 – 7.31 (m, 3H), 7.22 (d, *J* = 1.8 Hz, 1H), 7.03 (d, *J* = 7.0 Hz, 1H), 6.10 (dd, *J* = 3.5, 1.8 Hz, 1H), 6.07 (dd, *J* = 7.0, 2.0 Hz, 1H), 5.27 (d, *J* = 3.5 Hz, 1H), 4.92 (s, 1H), 4.27 (qd, *J* = 7.1, 1.3 Hz, 2H), 1.33 (t, *J* = 7.1 Hz, 3H) ppm.

^{13}C NMR (126 MHz, $CDCl_3$) δ 163.2, 151.3, 141.5, 140.7, 130.2, 128.8 (d, J_{CF} = 2.3 Hz), 128.7, 128.3, 126.5 (d, J_{CF} = 293.7 Hz), 123.6, 117.1, 111.7, 110.2 (d, J_{CF} = 2.1 Hz), 101.4, 66.7 (q, J_{CF} = 28.6 Hz), 61.8, 14.3 ppm.

^{19}F NMR (470 MHz, $CDCl_3$) δ -74.67 (s, 3F) ppm.

FT-IR: ṽ = 3321, 1703, 1627, 1545, 1577, 1402, 1375, 1277, 1208, 1174, 1105, 1074, 1026, 1013 cm^{-1}.

HRMS (ESI): calcd. for $[M+H]^+$ $C_{19}H_{17}F_3NO_3$ = 364.1155; found 364.1156.

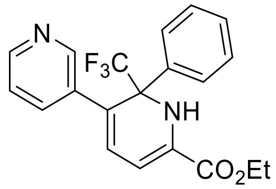

Ethyl 2-phenyl-2-(trifluoromethyl)-1,2-dihydro-[3,3'-bipyridine]-6-carboxylate (5n)

Prepared according to general procedure **A**. The product was isolated as yellow amorphous solid (45 mg, 0.12 mmol, 83%) (eluent: 5% EtOAc in *n*-pentane).

^{1}H NMR (500 MHz, $CDCl_3$) δ 8.37 (s, 1H), 8.07 (s, 1H), 7.58 (d, *J* = 7.5 Hz, 2H), 7.43 – 7.34 (m, 3H), 6.97 (dd, *J* = 8.1, 4.8 Hz, 1H), 6.89 – 6.82 (m, 1H), 6.36 (d, *J* = 6.6 Hz, 1H), 5.99 (dd, *J* = 6.5, 1.9 Hz, 1H), 5.13 (s, 1H), 4.30 (q, *J* = 7.1 Hz, 2H), 1.34 (t, *J* = 7.1 Hz, 3H) ppm.

^{13}C NMR (126 MHz, $CDCl_3$) δ 163.3, 149.7, 148.2, 140.2, 136.5, 135.4, 131.4, 129.1, 128.9, 128.8, 128.2 (d, J_{CF} = 2.3 Hz), 125.8 (d, J_{CF} = 291.7 Hz), 125.0, 122.4, 100.3, 68.4 (q, J_{CF} = 27.7 Hz), 62.0, 14.3 ppm.

^{19}F NMR (470 MHz, $CDCl_3$) δ -73.99 (s, 3F) ppm.

FT-IR: ṽ = 2981, 1744, 1709, 1682, 1636, 1476, 1449, 1373, 1260, 1155, 1073, 1045, 1024, 1011 cm^{-1}.

HRMS (ESI): calcd. for $[M+H]^+$ $C_{20}H_{18}F_3N_2O_2$ = 375.1315; found 375.1321.

3.3. Physical Data of Compounds 6a and 6b

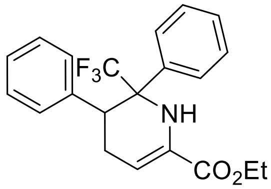

Ethyl 5,6-diphenyl-6-(trifluoromethyl)-1,4,5,6-tetrahydropyridine-2-carboxylate (6a)

^{1}H NMR (500 MHz, CDCl$_3$) δ 7.52 (d, *J* = 7.6 Hz, 2H), 7.44 – 7.36 (m, 3H), 7.34 – 7.27 (m, 5H), 5.85 (t, *J* = 4.2 Hz, 1H), 5.12 (s, 1H), 4.37 (m, 2H), 3.76 (d, *J* = 4.7 Hz, 1H), 2.29 – 2.15 (m, 2H), 1.40 (t, *J* = 7.1 Hz, 3H) ppm.

^{13}C NMR (126 MHz, CDCl$_3$) δ 163.8, 141.2, 138.5, 131.9, 129.1, 129.0, 128.6, 128.2, 127.3, 126.7, 124.4, 107.8, 65.4 (q, J_{CF} = 26.1 Hz), 61.7, 42.7, 28.2, 14. ppm.

^{19}F NMR (470 MHz, CDCl$_3$) δ -71.48 (s, 3F) ppm.

FT-IR: ṽ = 3398, 1705, 1655, 1478, 1447, 1374, 1283, 1271, 1242, 1213, 1172, 1152, 1109, 1018 cm^{-1}.

HRMS (ESI): calcd. for $[M+H]^+$ calcd. $C_{21}H_{21}F_3NO_2$ m/z 376.1519; found 376.1525.

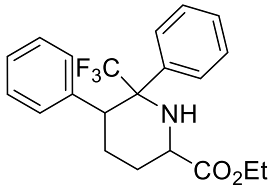

Ethyl 5,6-diphenyl-6-(trifluoromethyl) piperidine-2-carboxylate (6b)

^{1}H NMR (500 MHz, CDCl$_3$) δ7.73 (d, *J* = 7.5 Hz, 2H), 7.67 (d, *J* = 7.9 Hz, 2H), 7.49 (t, *J* = 7.8 Hz, 2H), 7.42 – 7.38 (m, 1H), 7.38 – 7.31 (m, 2H), 7.32 – 7.28 (m, 1H), 4.29 (q, *J* = 7.1 Hz, 2H), 3.80 (dd, *J* = 4.8, 2.9 Hz, 1H), 3.74 (dd, *J* = 12.0, 4.0 Hz, 1H), 2.14 – 2.02 (m, 1H), 1.91 – 1.79 (m, 1H), 1.72 – 1.60 (m, 2H), 1.34 (t, *J* = 7.1 Hz, 3H) ppm.

^{13}C NMR (126 MHz, CDCl$_3$) δ 173.2, 141.4, 135.6, 130.6, 129.0, 128.5, 128.4, 128.0, 127.0, 125.5 (d, J_{CF} = 285.5 Hz), 66.4 (q, J_{CF} = 25.5 Hz), 61.4, 52.8, 41.8, 27.3, 22.0, 14.3 ppm.

^{19}F NMR (470 MHz, CDCl$_3$) δ -72.79 (s, 3F) ppm.

FT-IR: ṽ = 2939, 2253, 1731, 1494, 1446, 1372, 1341, 1280, 1263, 1234, 1187, 1156, 1083, 1032 cm^{-1}.

HRMS (ESI): calcd. for $[M+H]^+$ calcd. $C_{21}H_{23}F_3NO_2$ m/z 378.1675; found 378.1679.

4. X-ray Analysis of Compounds 3a, 3b, 4a and 5a

The crystal structures of compounds **3a**, **3b**, **4a** and **5a** was determined using the *Bruker D8 Venture* four-circle diffractometer equipped with a *PHOTON II* CPAD detector by *Bruker AXS GmbH*. The X-ray radiation was generated by the *IμS* microfocus source Mo (λ = 0.71073 Å) from *Incoatec GmbH* equipped with HELIOS mirror optics and a single-hole collimator by *Bruker AXS GmbH*. The selected single crystal of **3a**, **3b**, **4a** and **5a** were covered with an inert oil (perfluoropolyalkyl ether) and mounted on the *MicroMount* from *MiTeGen*. The APEX 3 Suite (v.2018.7-2) software integrated with SAINT (integration) and SADABS (adsorption correction) programs by *Bruker AXS GmbH* were used for data collection. The processing and finalization of the crystal structure were performed using the Olex2 program.[8] The crystal structures were solved by the ShelXT[9] structure solution program using the Intrinsic Phasing option, which were further refined by the ShelXL[10] refinement package using Least Squares minimization. The non-hydrogen atoms were anisotropically refined. The C-bound H atoms were placed in geometrically calculated positions, and a fixed isotropic displacement parameter was assigned to each atom according to the riding-model: C–H = 0.95–1.00 Å with *U*iso(H) = 1.5*U*eq (CH3) and 1.2*U*eq (CH_2, CH) for other hydrogen atoms. The N-bound hydrogen atoms on N1, N2, N3 and N4 were located on the Difference-Fourier-Map and refined independently in every structure. The crystallographic data for the structures of **3a**, **3b**, **4a** and **5a** has been published as supplementary publication number 2060066 (**3a**), 2081540 (**3b**) 2046825 (**4a**), 2046566 (**5a**) in the Cambridge Crystallographic Data Centre. A copy of these data can be obtained for free by applying to CCDC, 12 Union Road, Cambridge CB2 IEZ, UK, fax: 144-(0)1223-336033 or e-mail: deposit@ccdc.cam.ac.uk.

Crystal Structure for Compound 3a

CCDC Number: 2060066

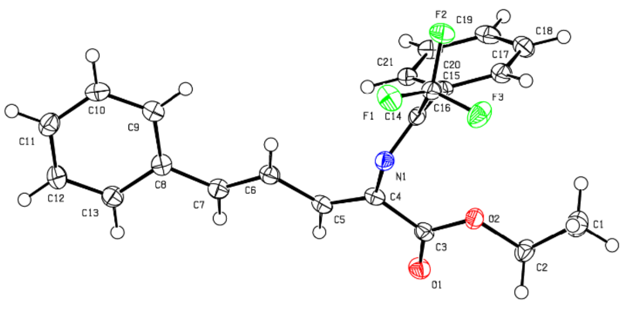

Figure 1. Ortep plot of the molecular structure in the crystal of compound 3a.[11] The displacement ellipsoids are drawn at the 50% probability level. Co-crystallized solvent has been omitted for clarity.

Table 1. Crystallographic data of compound 3a.

**Compound**	**3a**
Empirical formula	$C_{21}H_{18}F_3NO_2$
Formula weight	373.36
Temperature/K	100.0
Crystal system	monoclinic
Space group	$P2_1/c$
a/Å	15.4189(10)
b/Å	13.2984(9)
c/Å	8.8308(5)
α/°	90
β/°	98.773(2)
γ/°	90
Volume/Å^3	1789.5(2)
Z	4
ρ_{calc}g/cm^3	1.386
μ/mm^{-1}	0.110
F(000)	776.0
Crystal size/mm^3	0.121 × 0.116 × 0.1
Radiation	MoKα (λ = 0.71073)
2Θ range for data collection/°	4.066 to 52.74
Index ranges	$-19 \leq h \leq 19$,<br>$-16 \leq k \leq 16$,<br>$-11 \leq l \leq 11$
Reflections collected	27328
Independent reflections	3655 [R_{int} = 0.0978,<br>R_{sigma} = 0.0512]
Data/restraints/parameters	3655/0/245
Goodness-of-fit on F^2	1.069
Final *R* indexes [I>=2σ (I)]	R_1 = 0.0489,<br>wR_2 = 0.0962
Final *R* indexes [all data]	R_1 = 0.0842,<br>wR_2 = 0.1145
Largest diff. peak/hole / e Å^{-3}	0.22/-0.26
Flack parameter	–

Crystal Structure for Compound 3b

CCDC Number: 2081540

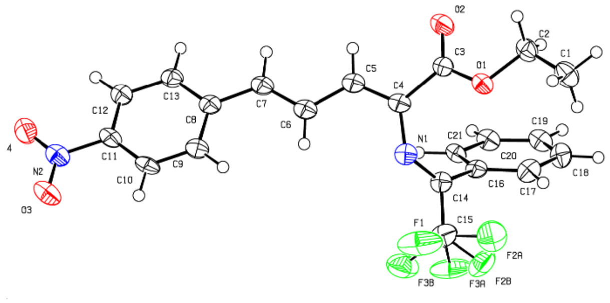

Figure 2. Ortep plot of the molecular structure in the crystal of compound 3b.[11] The displacement ellipsoids are drawn at the 50% probability level. Co-crystallized solvent has been omitted for clarity.

Table 2. Crystallographic data of compound 3b.

**Compound**	**3b**
Empirical formula	$C_{21}H_{17}F_3N_2O_4$
Formula weight	418.36
Temperature/K	100.0
Crystal system	Triclinic
Space group	*P*-1
a/Å	7.6686(17)
b/Å	8.2975(19)
c/Å	17.313(4)
α/°	89.757(8)
β/°	78.124(8)
γ/°	65.124(7)
Volume/Å^3	973.8(4)
Z	2
ρ_{calc}g/cm^3	1.427
μ/mm^{-1}	0.118
F(000)	432.0
Crystal size/mm^3	0.238 × 0.147 × 0.074
Radiation	MoKα (λ = 0.71073)
2Θ range for data collection/°	5.436 to 52.884
Index ranges	$-9 \leq h \leq 9$,<br>$-10 \leq k \leq 10$,<br>$-21 \leq l \leq 21$
Reflections collected	21521
Independent reflections	3982 [R_{int} = 0.1023,<br>R_{sigma} = 0.0743]
Data/restraints/parameters	3982/0/306
Goodness-of-fit on F^2	1.108

Final *R* indexes [I>=2σ (I)]	R_1 = 0.0974, wR_2 = 0.2314
Final *R* indexes [all data]	R_1 = 0.1401, wR_2 = 0.2511
Largest diff. peak/hole / e Å^{-3}	0.41/–0.48
Flack parameter	–

Crystal Structure for Compound 4a

CCDC Number: 2046825

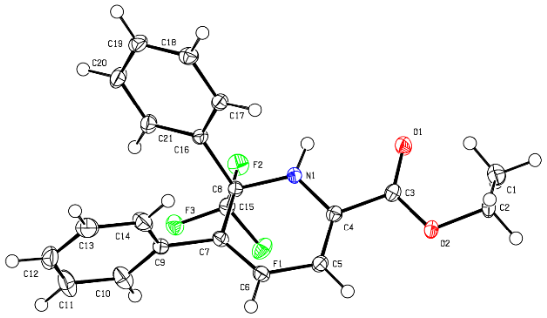

Figure 3. Ortep plot of the molecular structure in the crystal of compound 4a.[11] The displacement ellipsoids are drawn at the 50% probability level.

Table 3. Crystallographic data of compound 4a.

**Compound**	**4a**
Empirical formula	$C_{21}H_{18}F_3NO_2$
Formula weight	373.36
Temperature/K	100.0
Crystal system	monoclinic
Space group	$P2_1/c$
a/Å	14.6948(6)
b/Å	6.2081(3)
c/Å	19.2023(8)
α/°	90
β/°	99.111(2)
γ/°	90
Volume/Å^3	1729.66(13)
Z	4
ρ_{calc}g/cm^3	1.434
μ/mm^{-1}	0.114
F(000)	776.0
Crystal size/mm^3	0.209 × 0.139 × 0.091
Radiation	MoKα (*λ* = 0.71073)
2Θ range for data collection/°	5.492 to 60

Index ranges	$-20 \le h \le 20$, $-8 \le k \le 8$, $-27 \le l \le 21$
Reflections collected	34035
Independent reflections	5056 [R_{int} = 0.0626, R_{sigma} = 0.0406]
Data/restraints/parameters	5056/0/249
Goodness-of-fit on F^2	1.034
Final R indexes [I>=2σ (I)]	R_1 = 0.0476, wR_2 = 0.0989
Final R indexes [all data]	R_1 = 0.0743, wR_2 = 0.1128
Largest diff. peak/hole / e Å^{-3}	0.34/–0.27
Flack parameter	–

Crystal Structure for Compound 5a

CCDC Number: 2046566

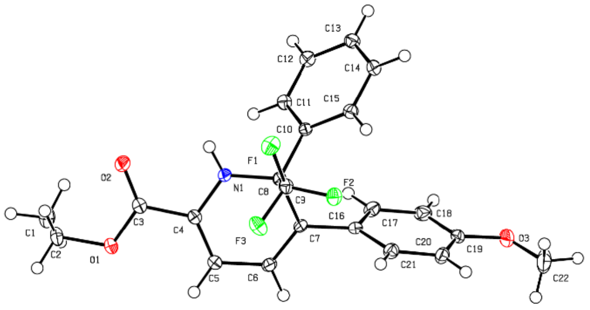

Figure 4. *Ortep plot of the molecular structure in the crystal of compound 5a.*[11] *The displacement ellipsoids are drawn at the 50% probability level.*

Table 4. Crystallographic data of compound 5a.

**Compound**	**5a**
Empirical formula	$C_{22}H_{20}F_3NO_3$
Formula weight	403.39
Temperature/K	100.0
Crystal system	monoclinic
Space group	$P2_1/n$
a/Å	14.374(3)
b/Å	6.0868(7)
c/Å	21.363(3)
α/°	90
β/°	92.254(8)
γ/°	90
Volume/Å^3	1861.2(5)
Z	4

ρ_{calc}g/cm^3	1.440
μ/mm^{-1}	0.115
F(000)	840.0
Crystal size/mm^3	0.275 × 0.168 × 0.118
Radiation	MoKα (λ = 0.71073)
2Θ range for data collection/°	5.692 to 59.996
Index ranges	$-20 \leq h \leq 20$,<br>$-8 \leq k \leq 8$,<br>$-29 \leq l \leq 30$
Reflections collected	20668
Independent reflections	5406 [R_{int} = 0.0306,<br>R_{sigma} = 0.0284]
Data/restraints/parameters	5426/0/268
Goodness-of-fit on F^2	1.050
Final *R* indexes [I>=2σ (I)]	R_1 = 0.0422,<br>wR_2 = 0.0994
Final *R* indexes [all data]	R_1 = 0.0527,<br>wR_2 = 0.1077
Largest diff. peak/hole / e Å^{-3}	0.43/–0.27
Flack parameter	–

5. Copies of NMR spectra

^{1}H NMR (500 MHz, $CDCl_3$)

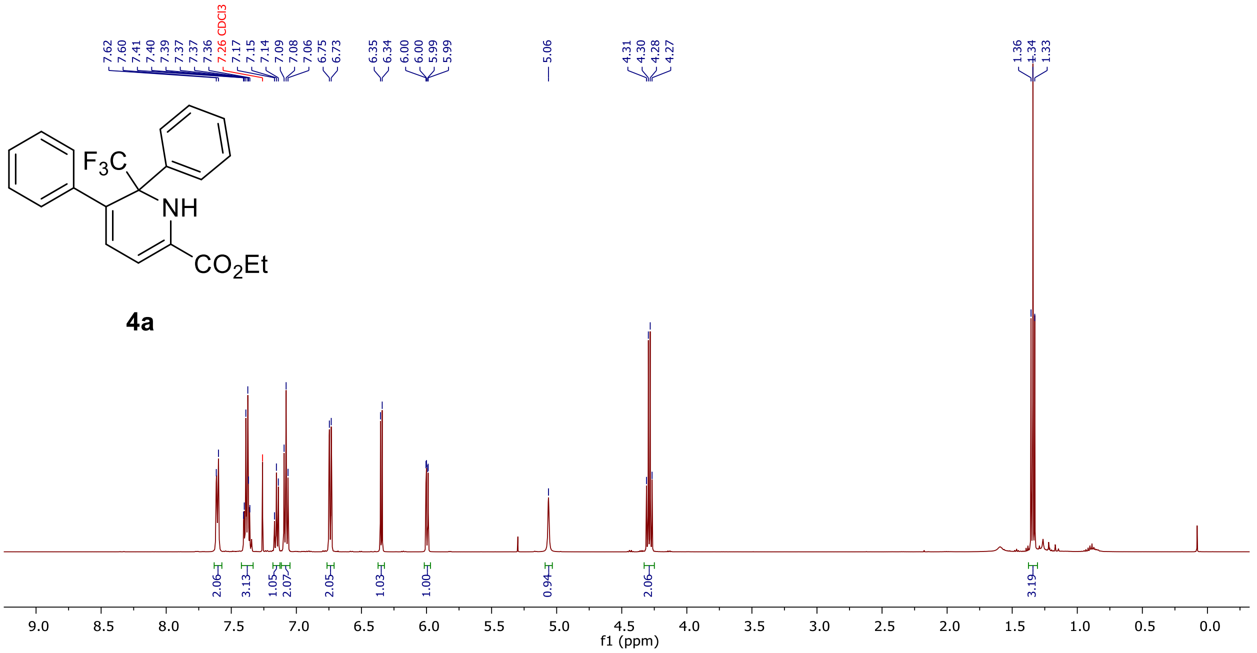

^{13}C NMR (126 MHz, $CDCl_3$)

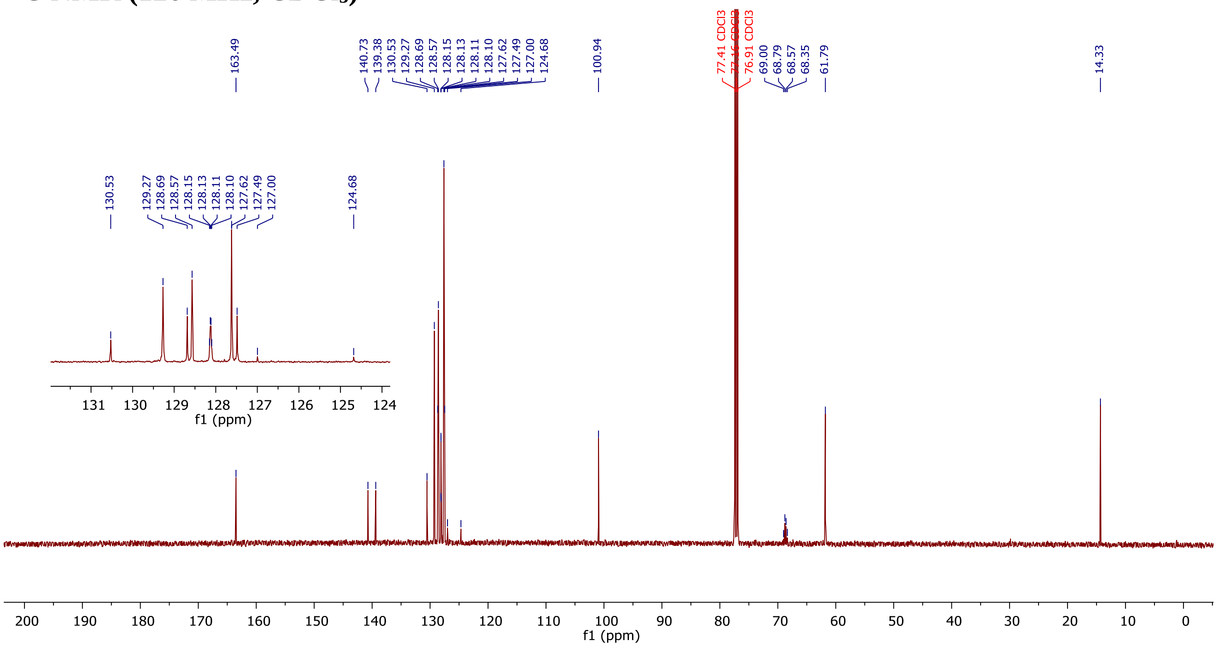

^{19}F NMR (470 MHz, $CDCl_3$)

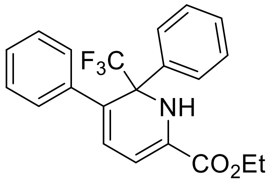

4a

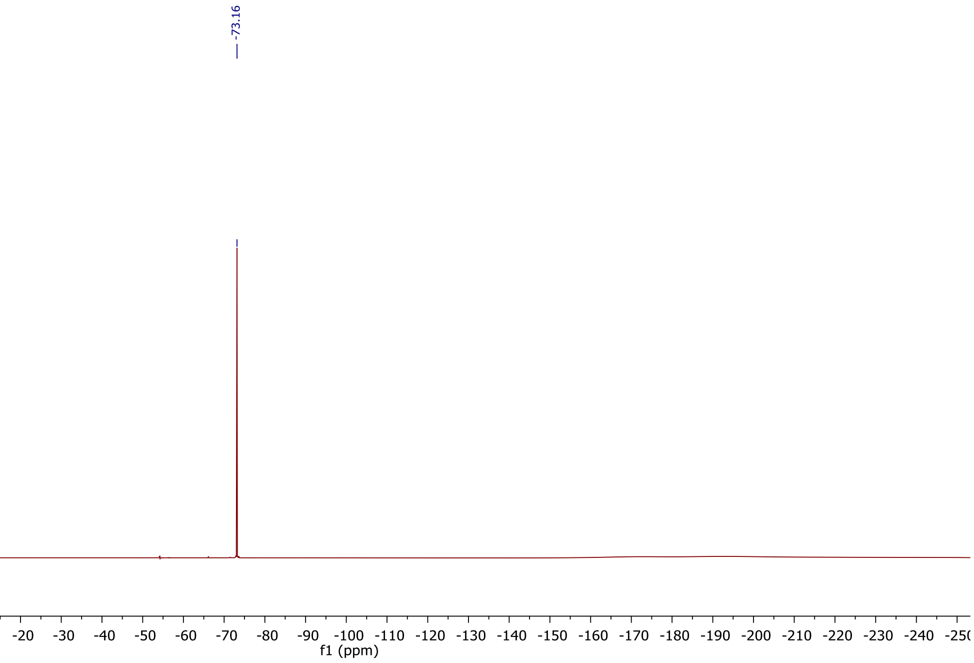

^{1}H NMR (400 MHz, $CDCl_3$)

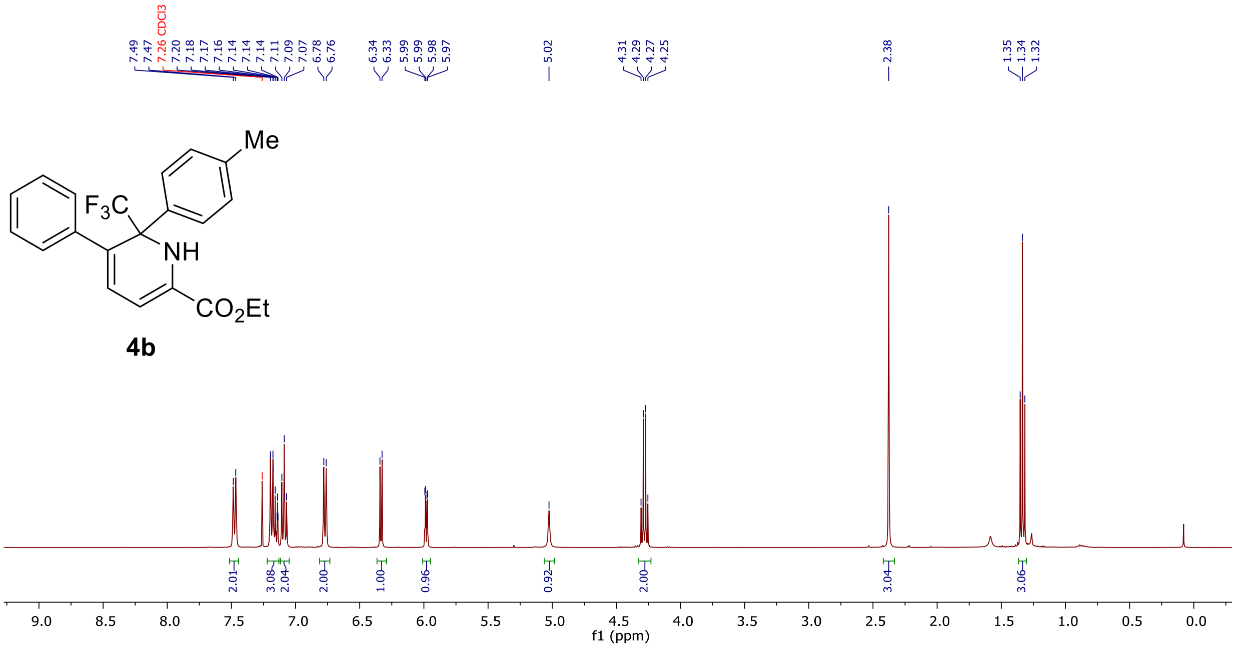

^{13}C NMR (101 MHz, $CDCl_3$)

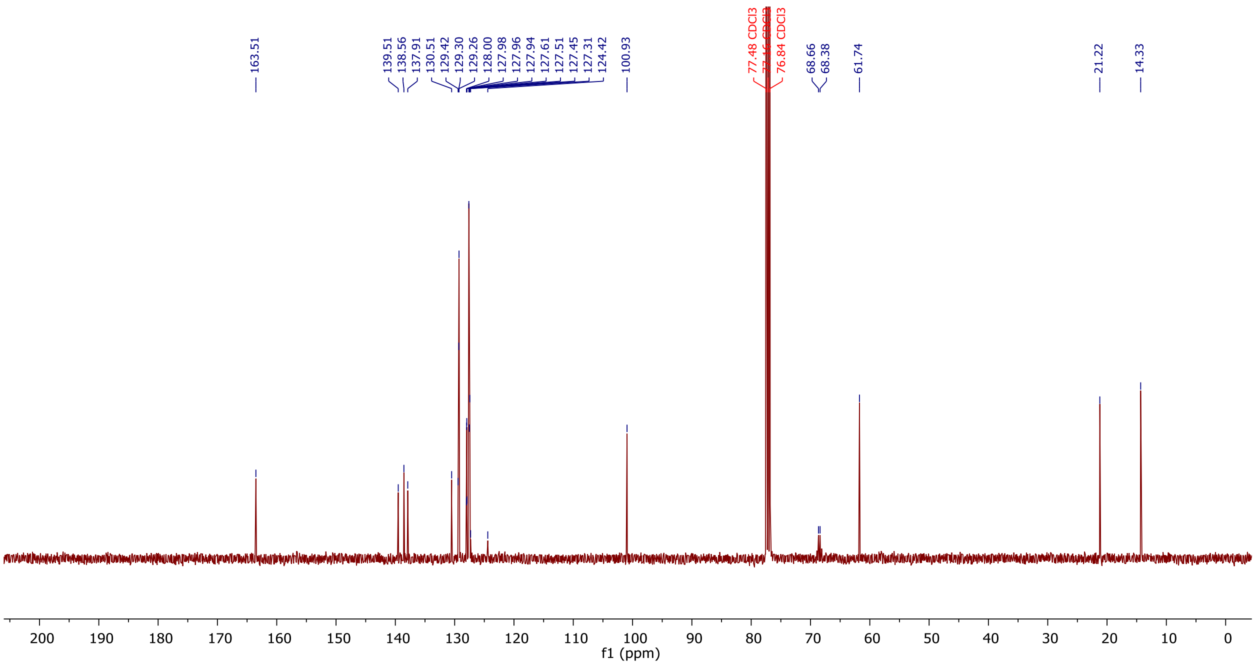

^{19}F NMR (377 MHz, $CDCl_3$)

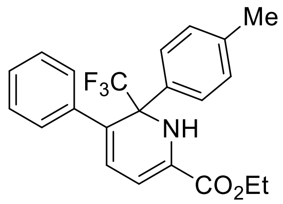

4b

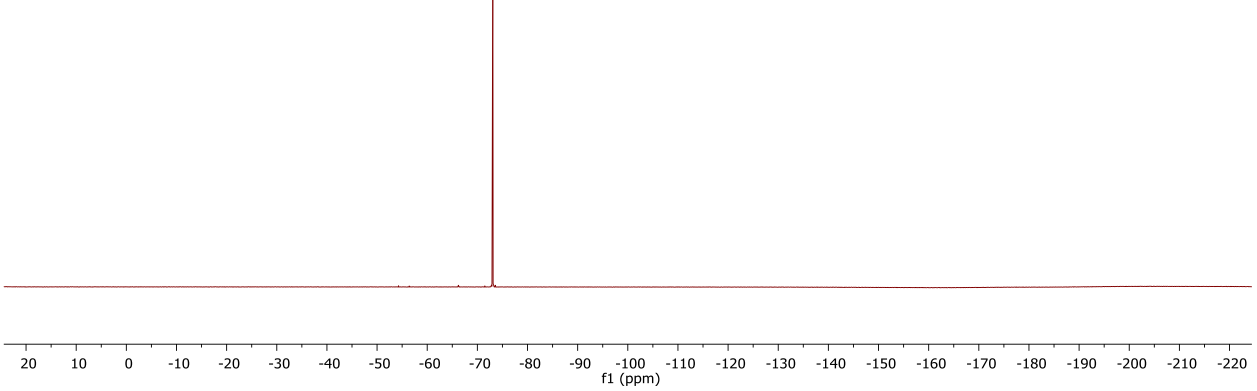

^{1}H NMR (500 MHz, CDCl$_3$)

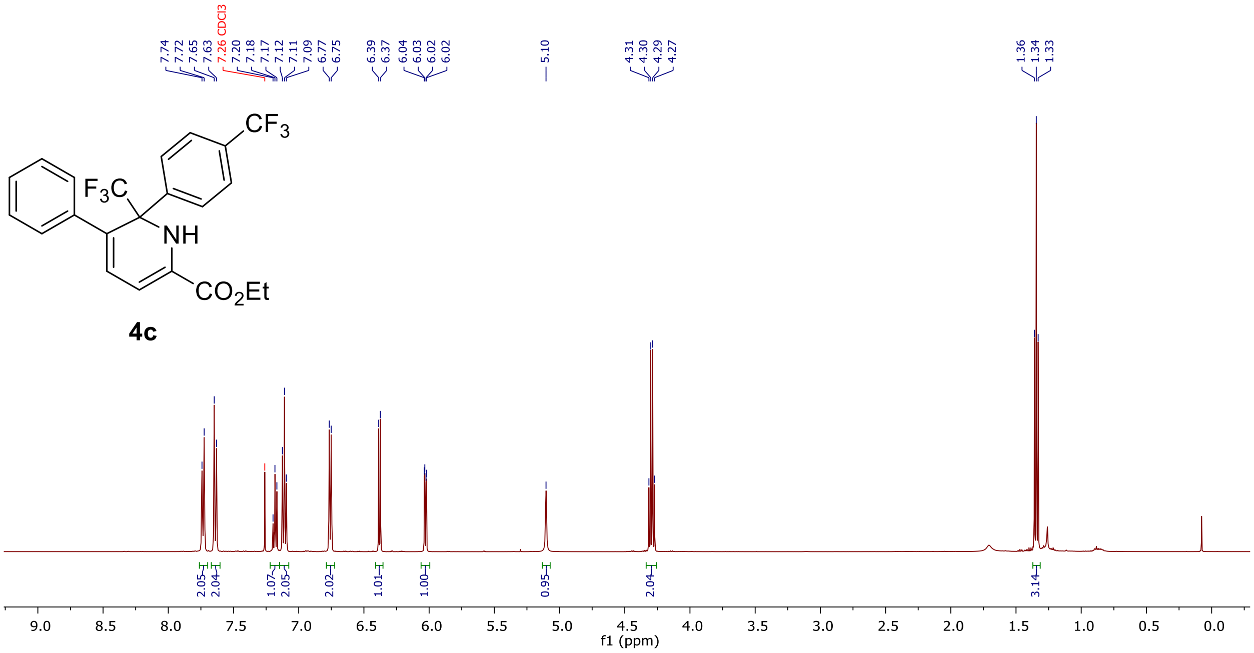

4c

^{13}C NMR (126 MHz, CDCl$_3$)

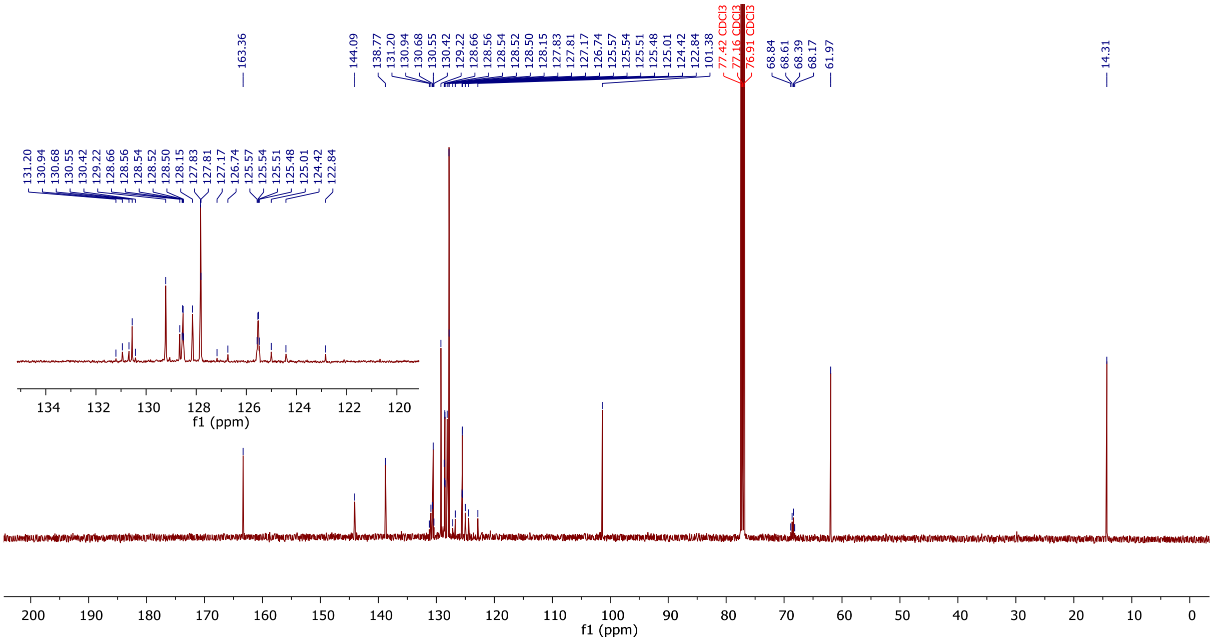

^{19}F NMR (470 MHz, $CDCl_3$)

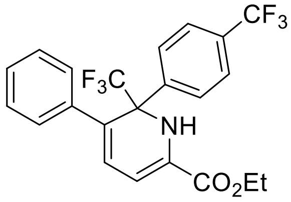

4c

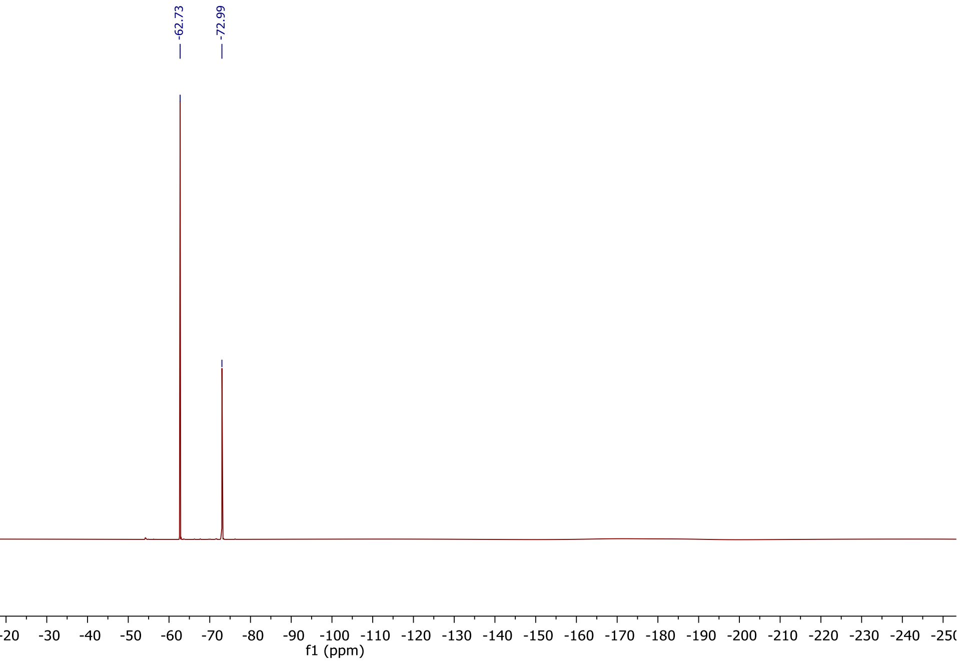

^{1}H NMR (500 MHz, $CDCl_3$)

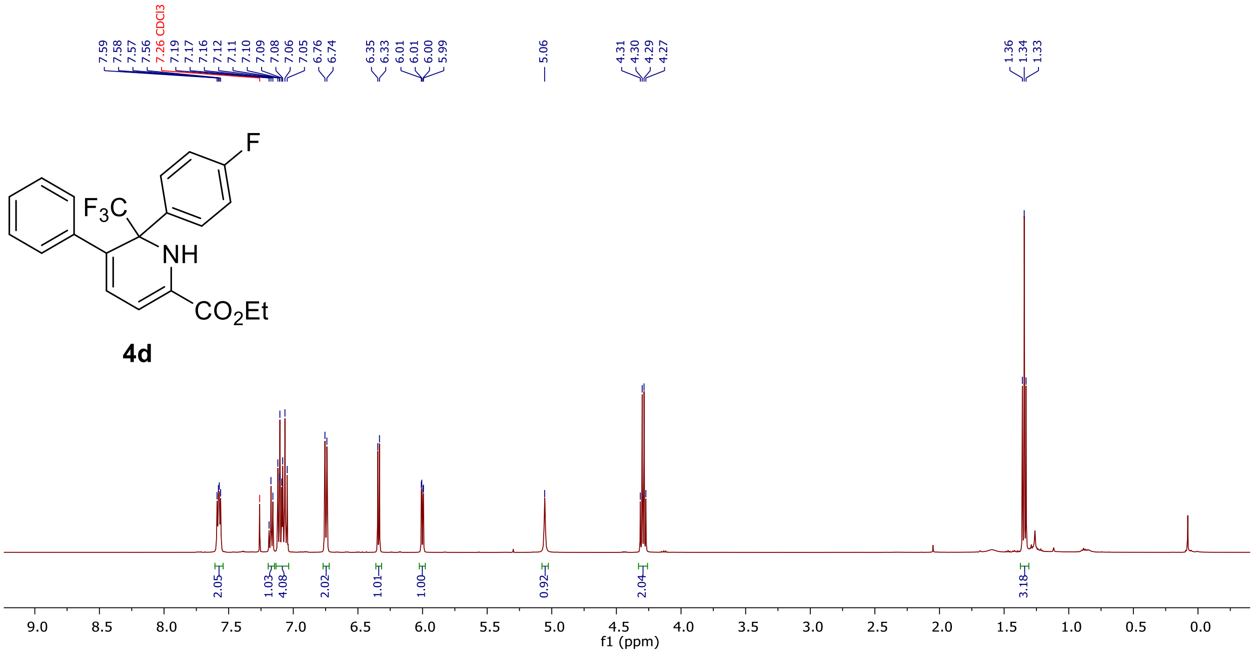

^{13}C NMR (126 MHz, $CDCl_3$)

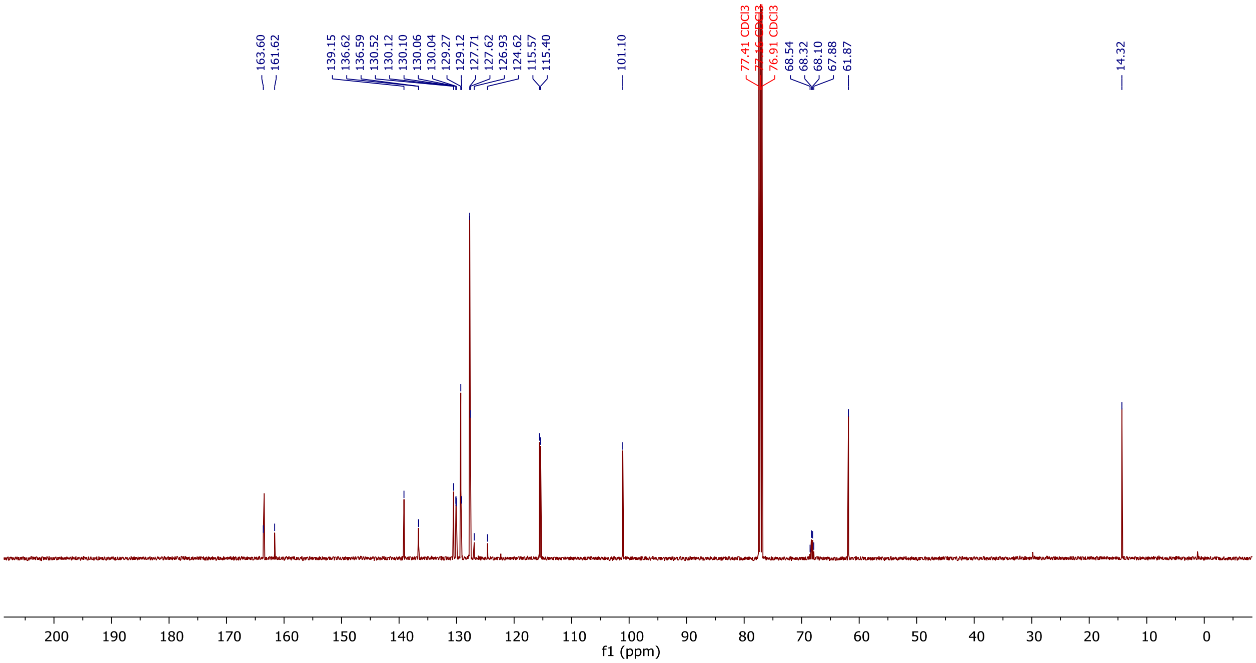

^{19}F NMR (470 MHz, $CDCl_3$)

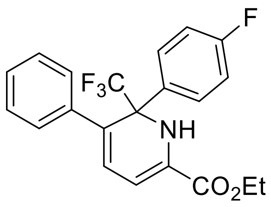

4d

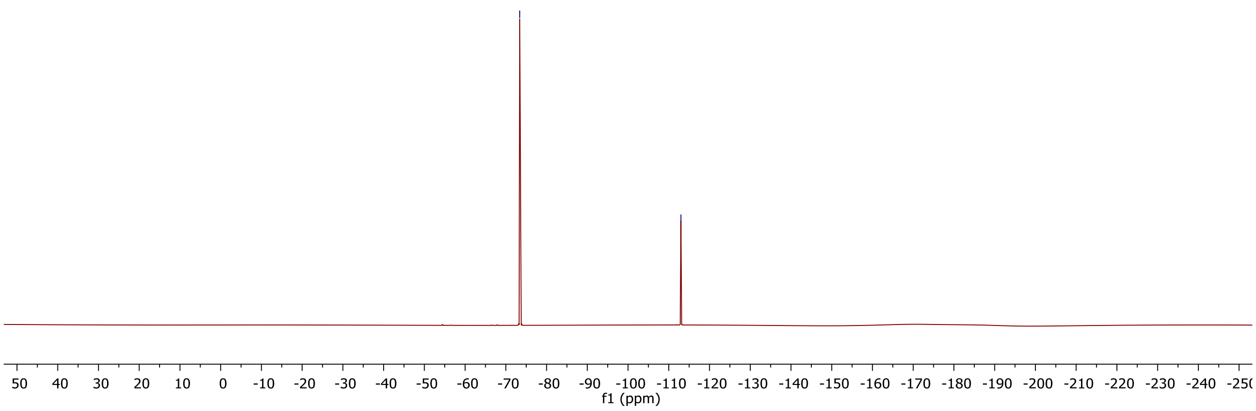

^{1}H NMR (500 MHz, $CDCl_3$)

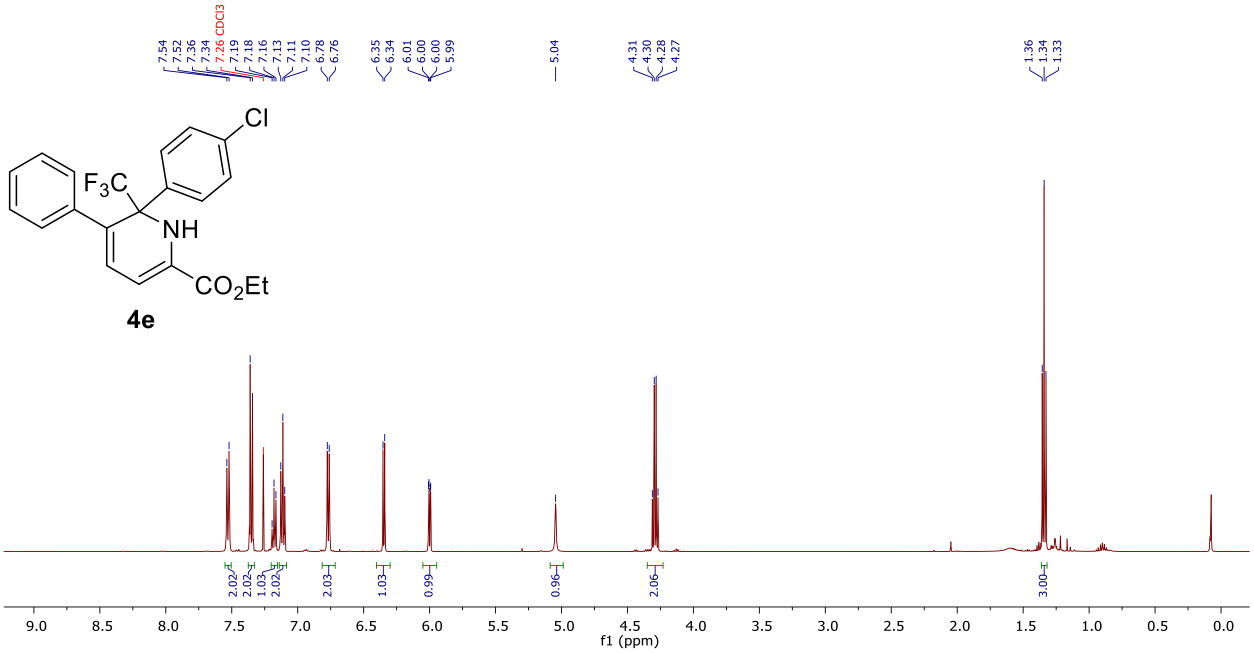

^{13}C NMR (126 MHz, $CDCl_3$)

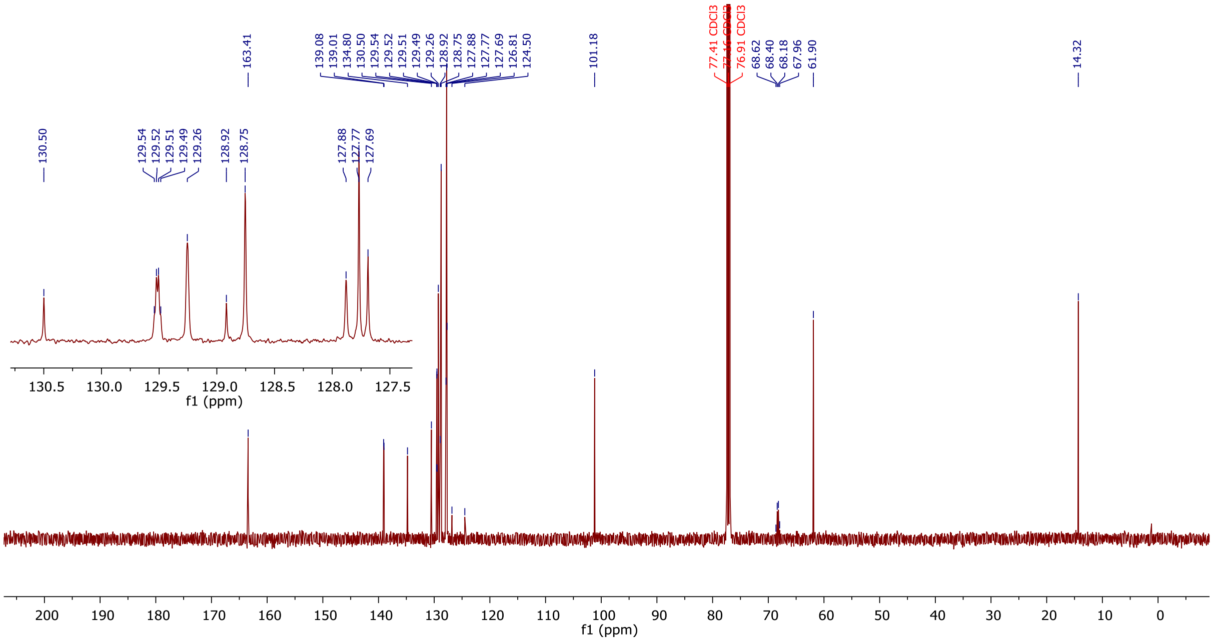

^{19}F NMR (470 MHz, $CDCl_3$)

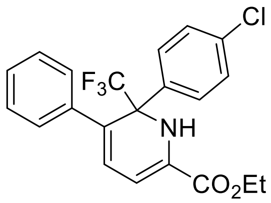

4e

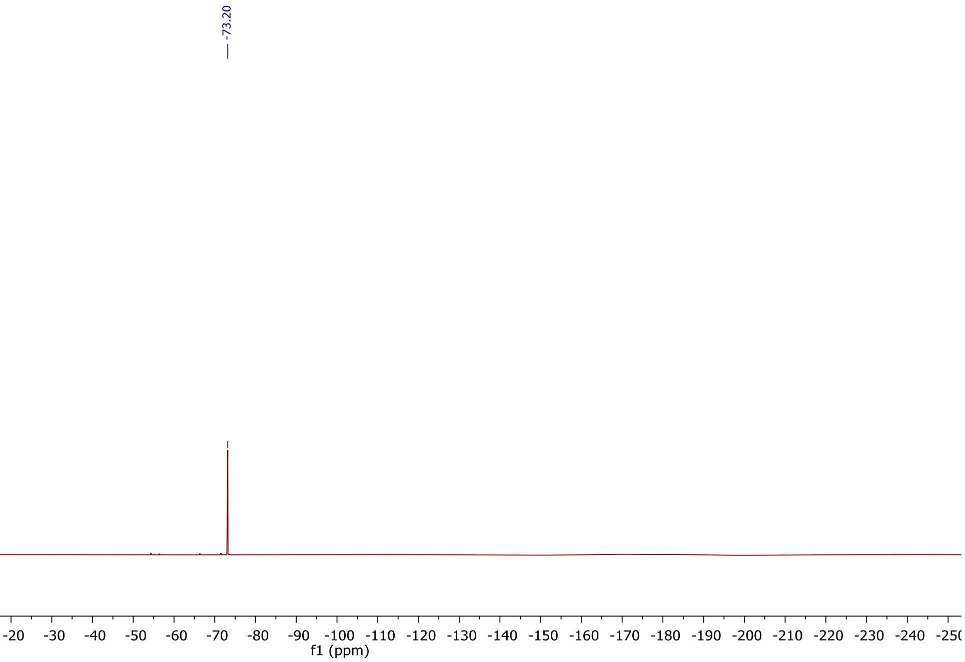

^{1}H NMR (500 MHz, $CDCl_3$)

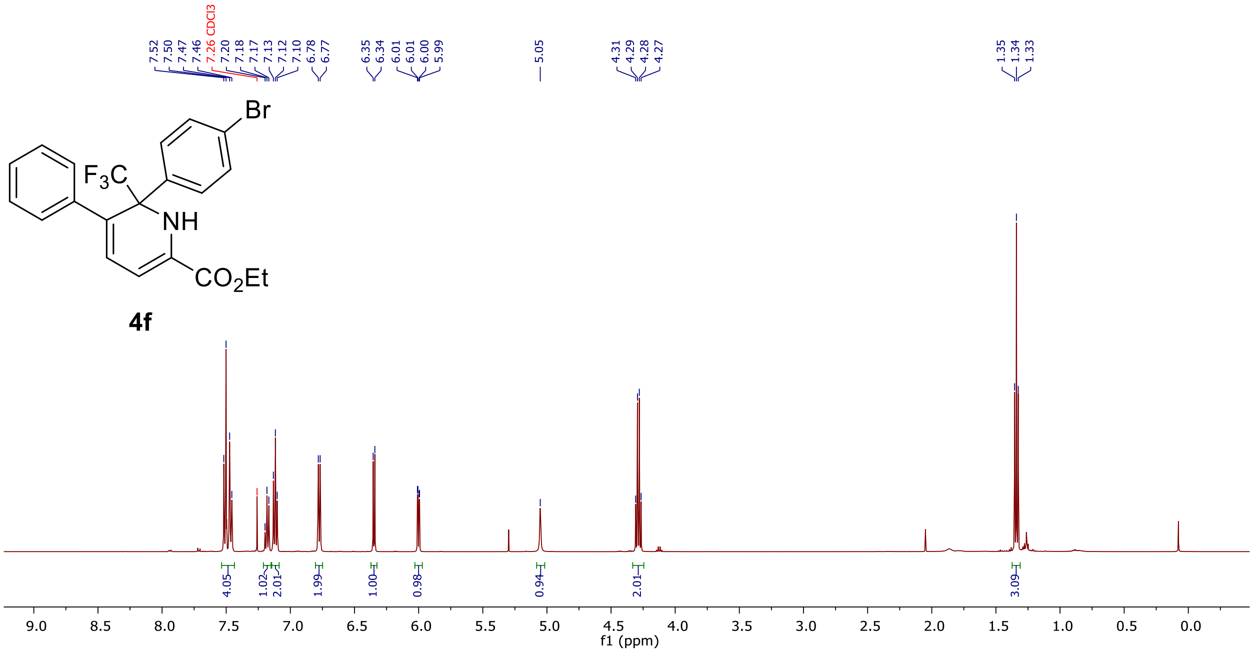

^{13}C NMR (126 MHz, $CDCl_3$)

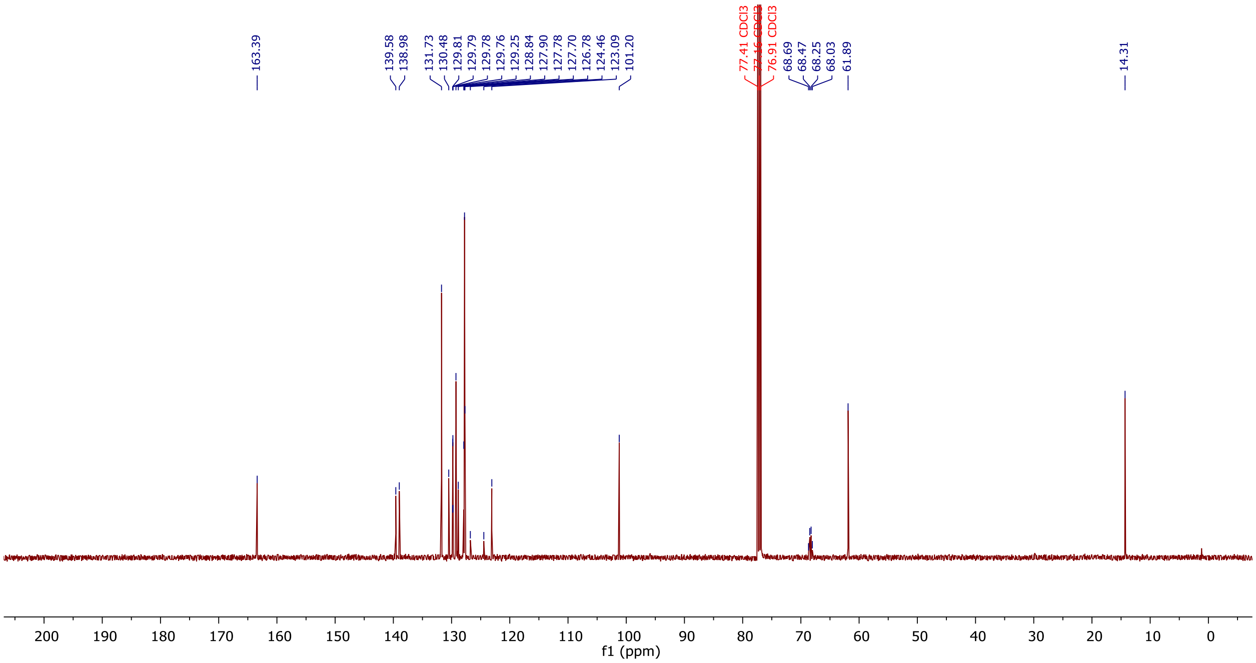

^{19}F NMR (470 MHz, $CDCl_3$)

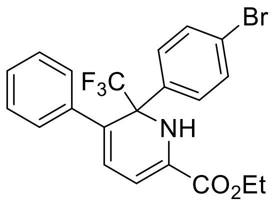

4f

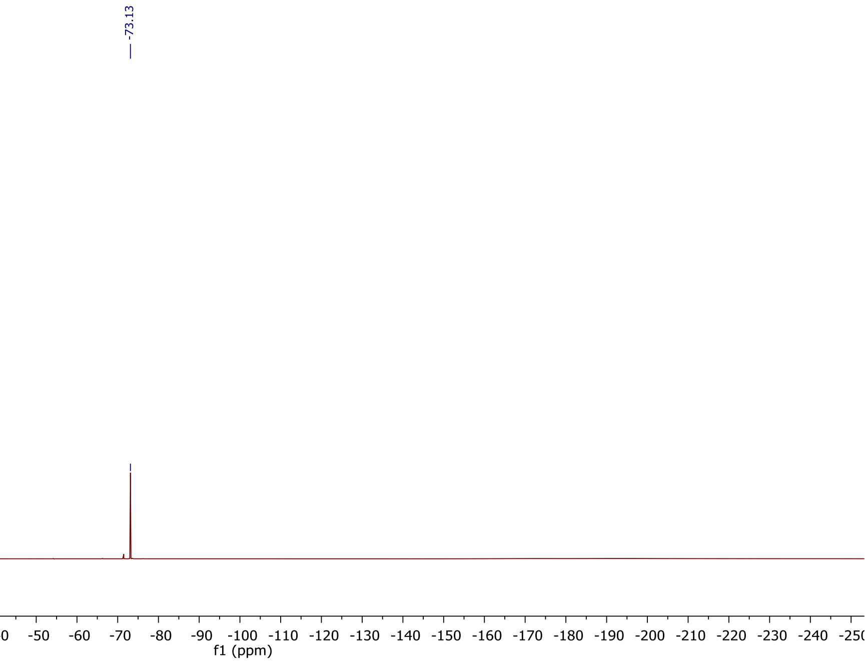

^{1}H NMR (500 MHz, $CDCl_3$)

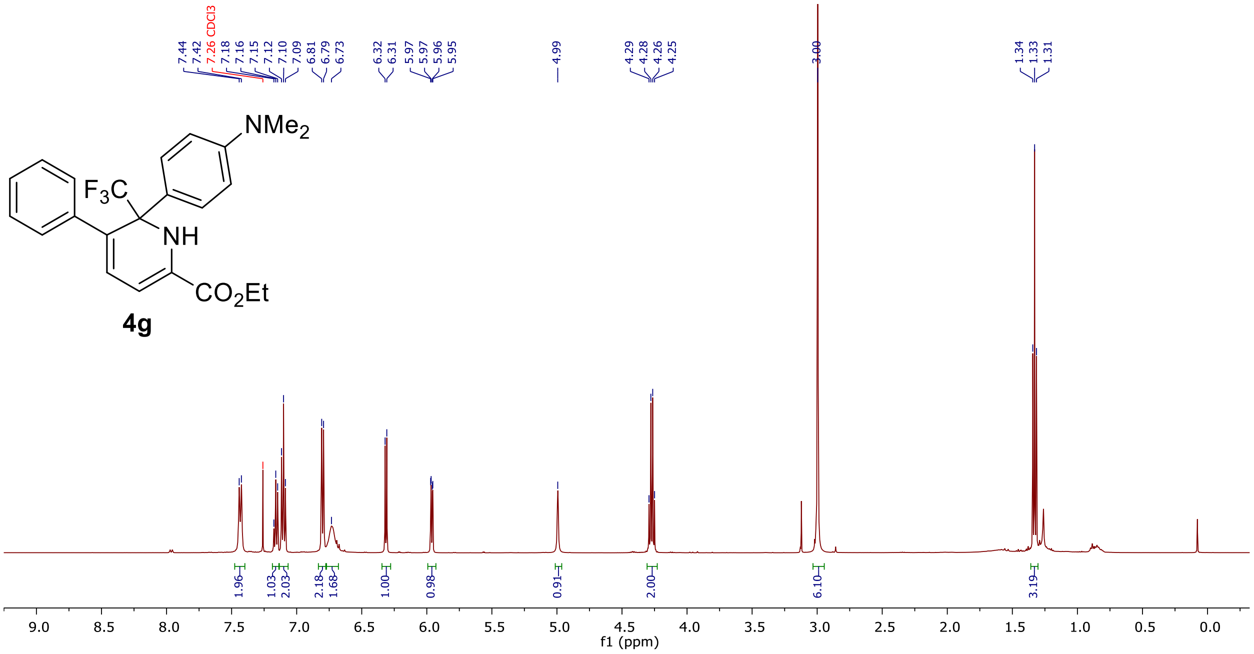

^{13}C NMR (126 MHz, $CDCl_3$)

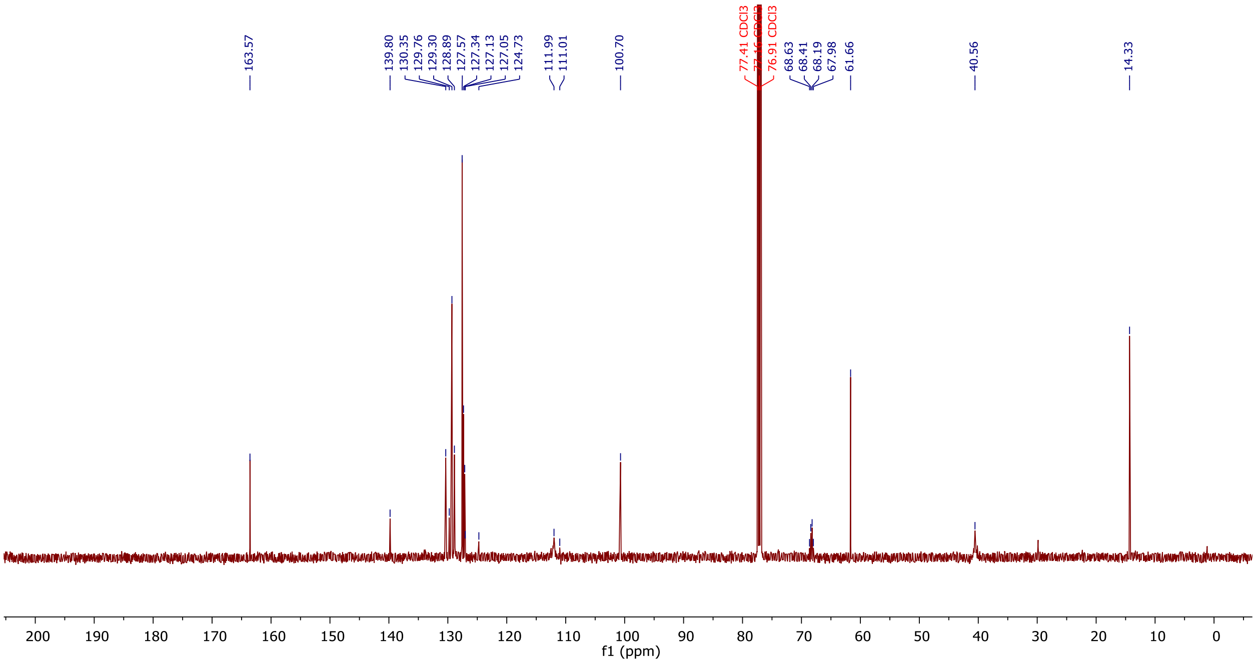

^{19}F NMR (470 MHz, $CDCl_3$)

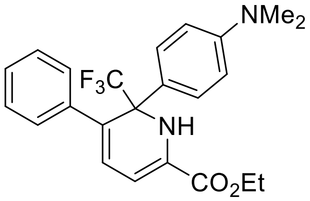

4g

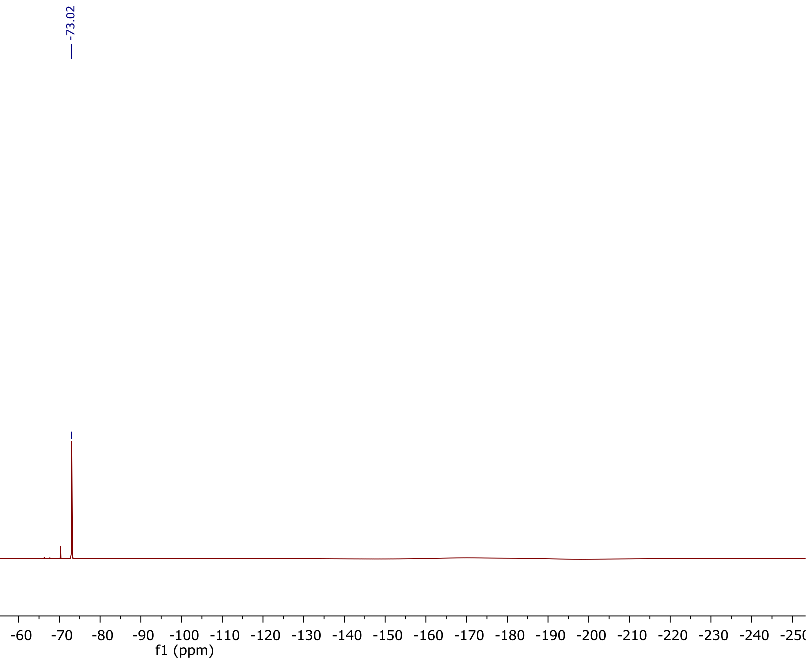

^{1}H NMR (400 MHz, $CDCl_3$)

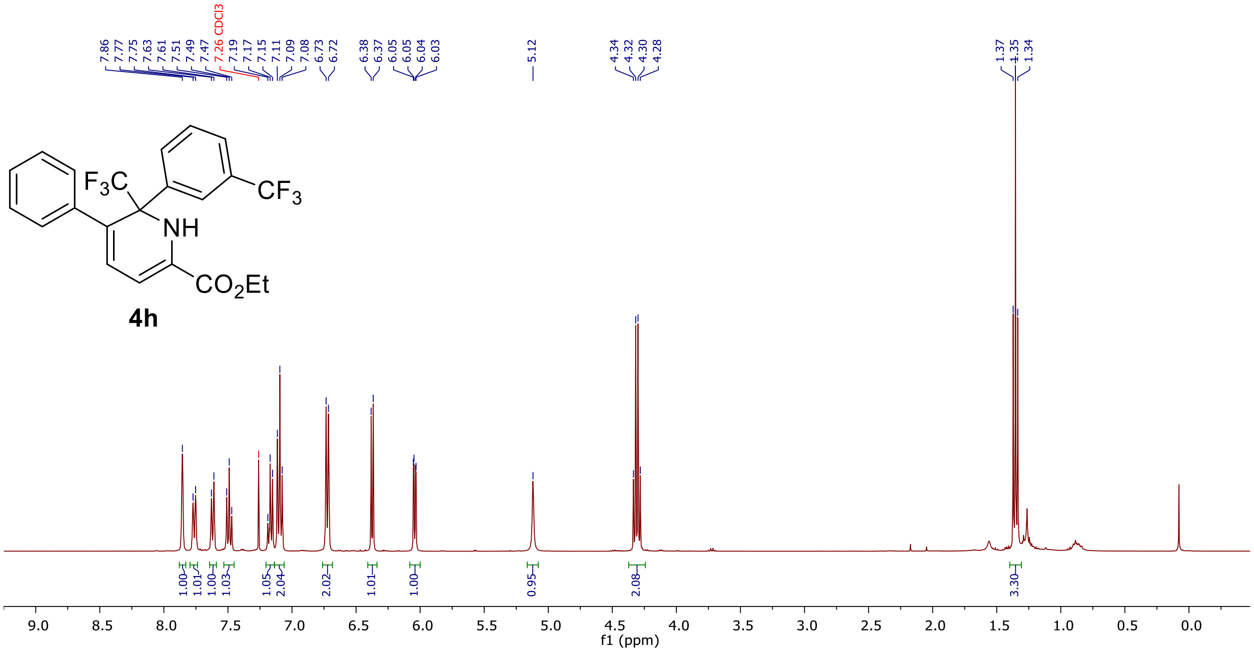

^{13}C NMR (101 MHz, $CDCl_3$)

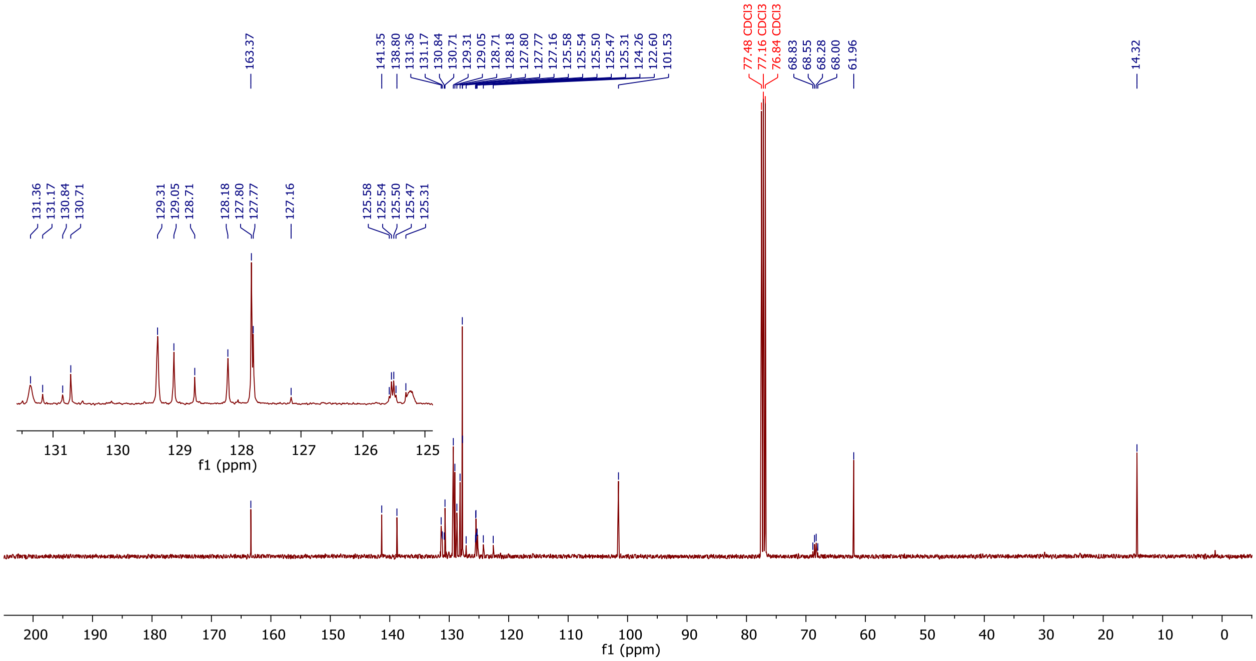

^{19}F NMR (377 MHz, $CDCl_3$)

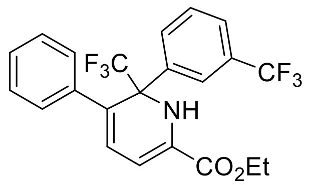

4h

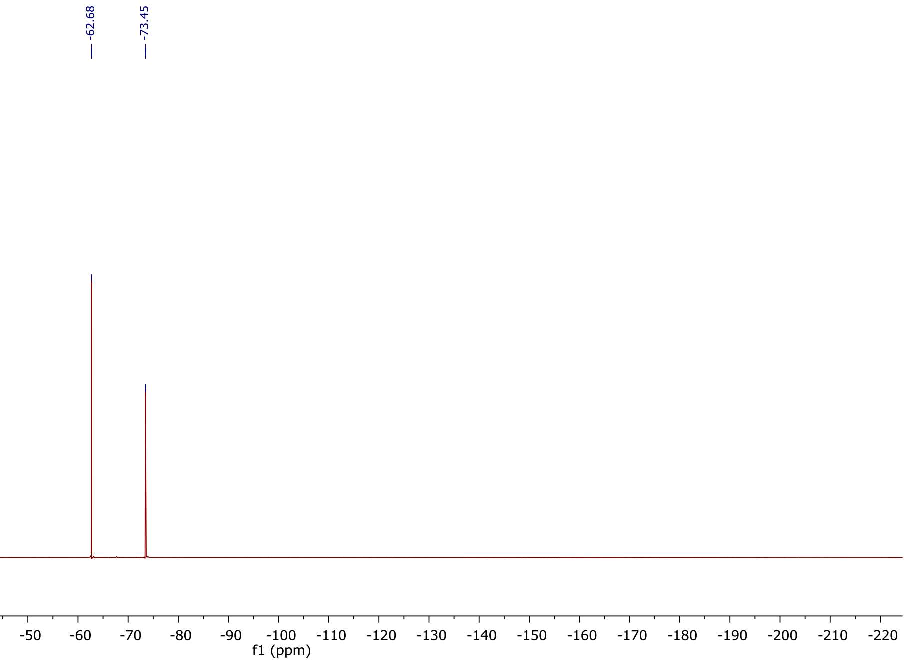

^{1}H NMR (500 MHz, $CDCl_3$)

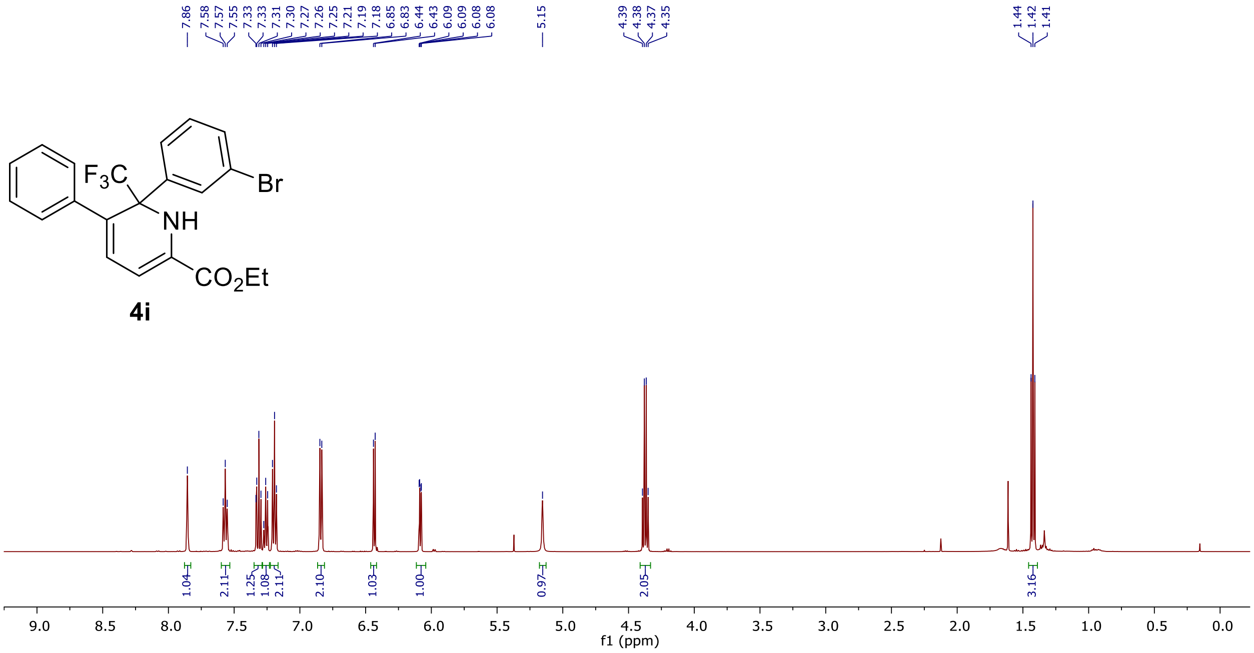

^{13}C NMR (126 MHz, $CDCl_3$)

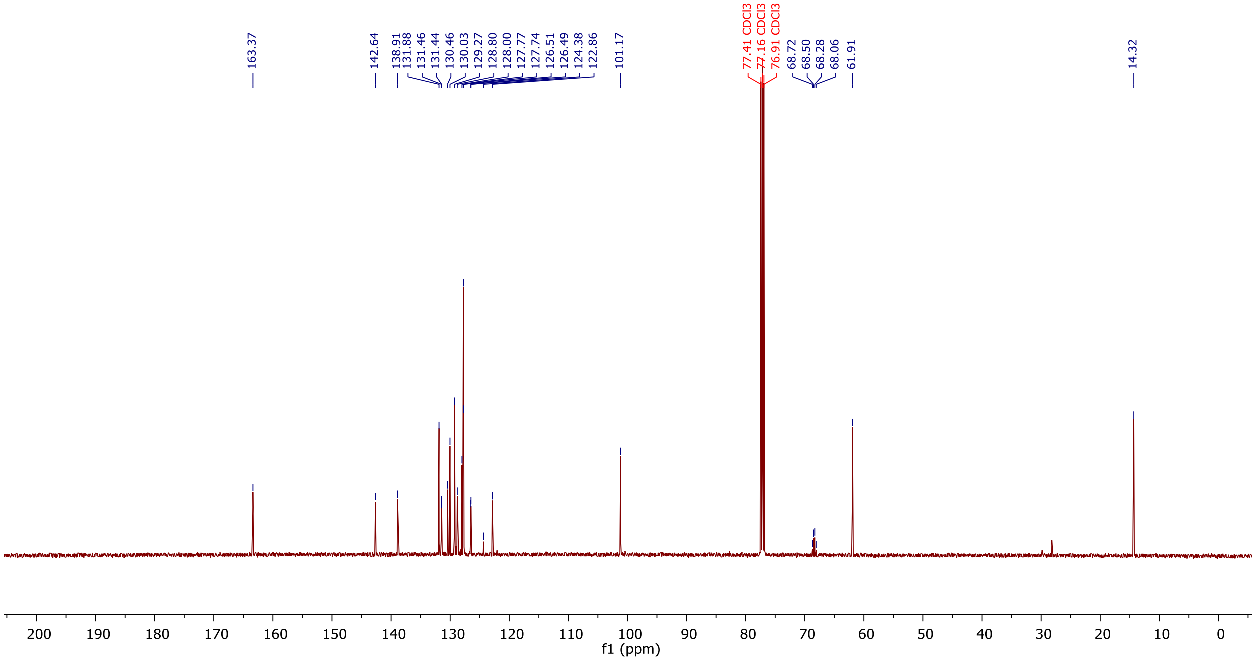

^{19}F NMR (470 MHz, $CDCl_3$)

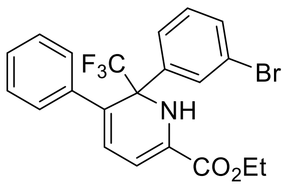

4i

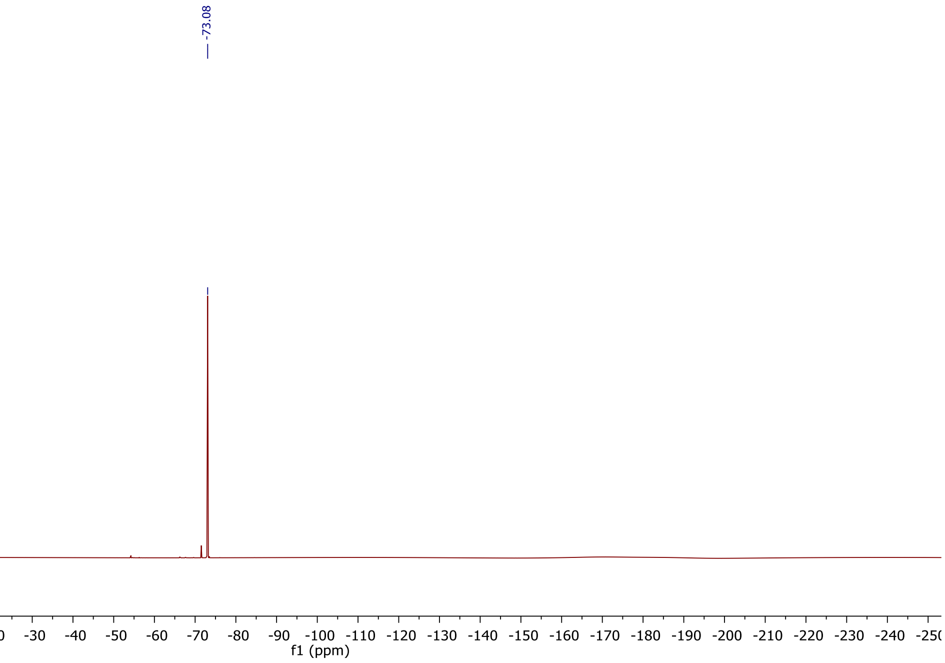

^{1}H NMR (600 MHz, $CDCl_3$)

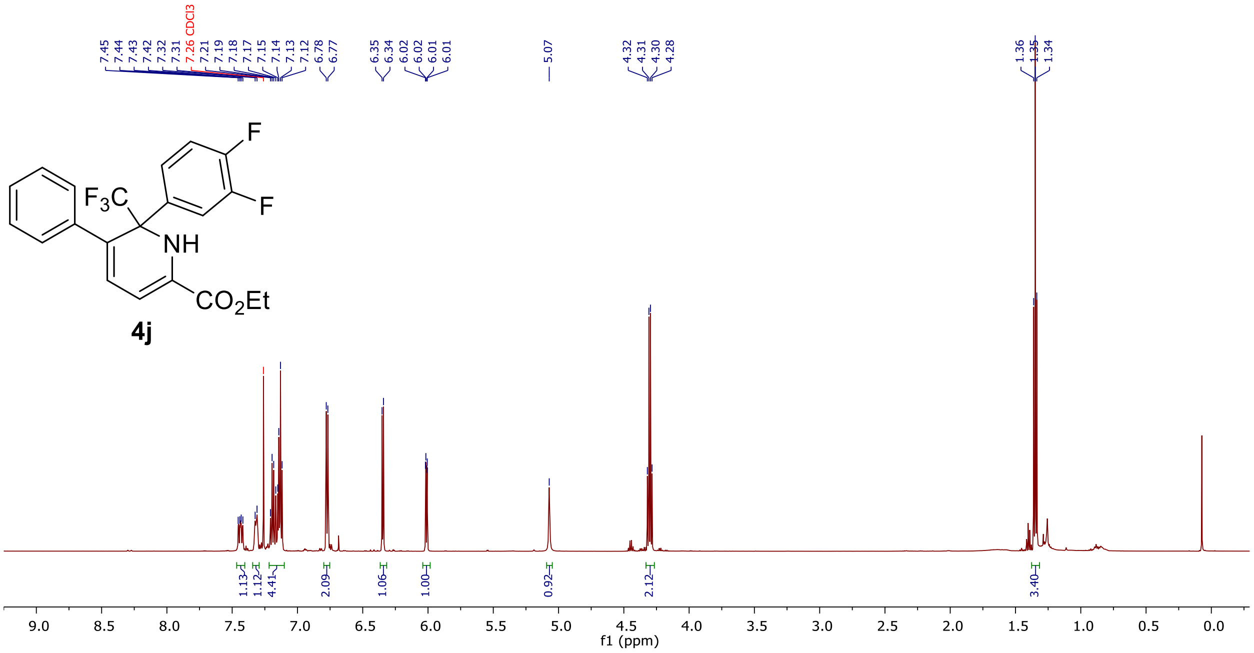

^{13}C NMR (151 MHz, $CDCl_3$)

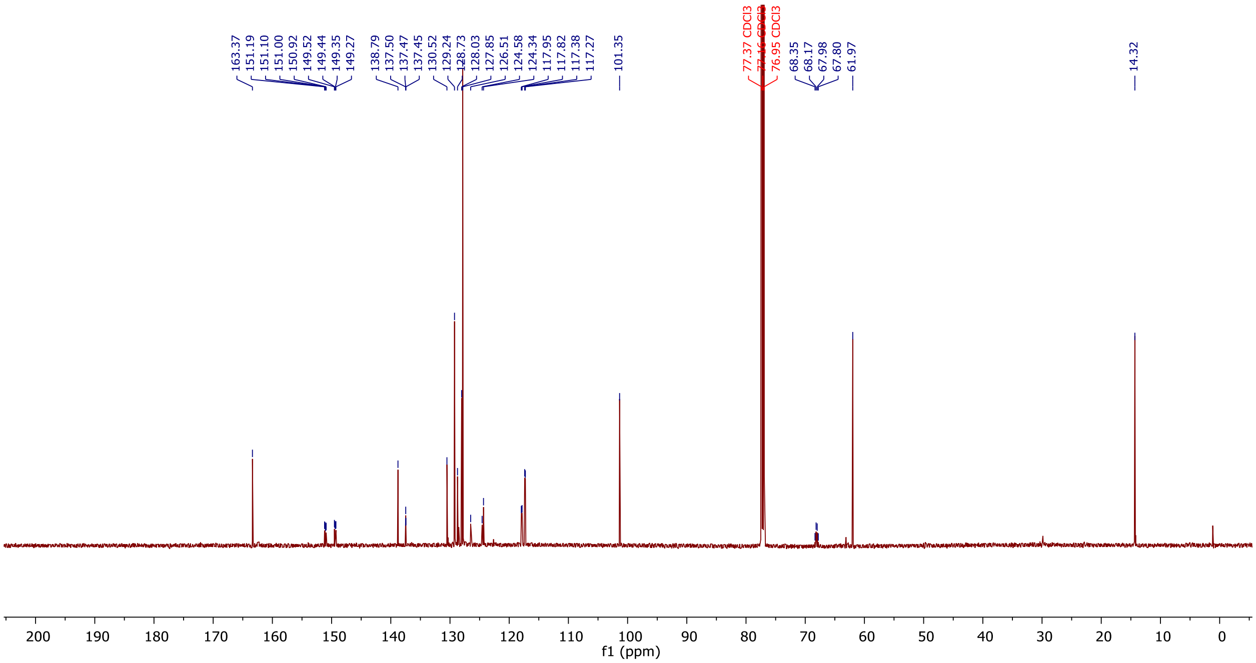

^{19}F NMR (470 MHz, $CDCl_3$)

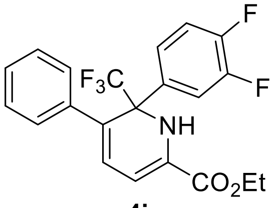

4j

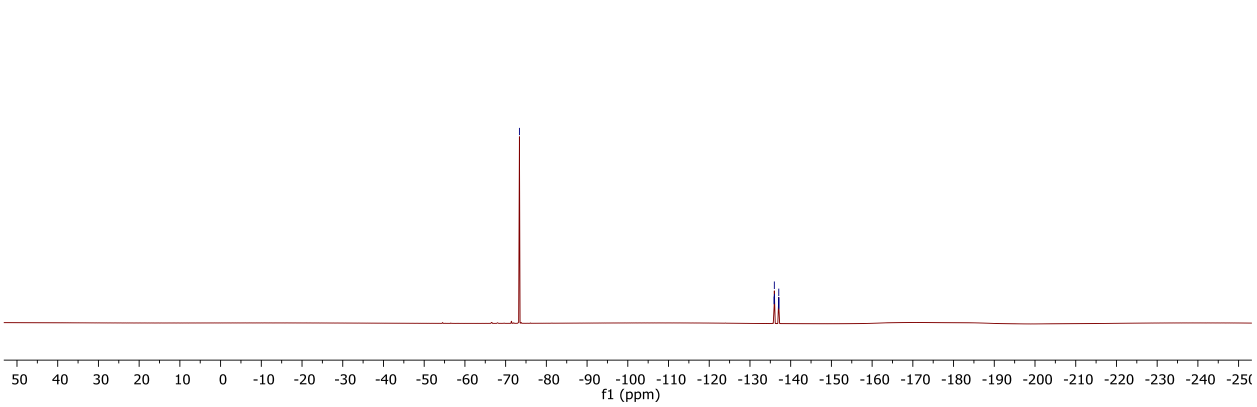

^{1}H NMR (500 MHz, $CDCl_3$)

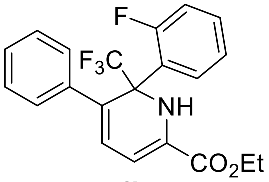

4k

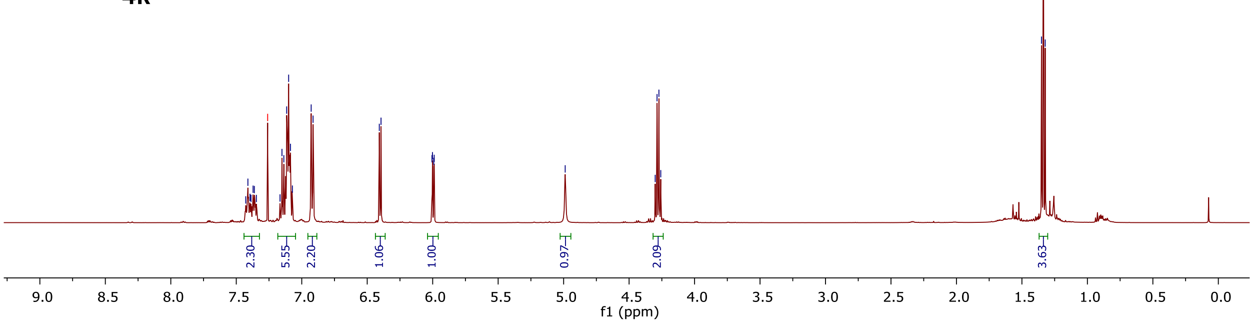

^{13}C NMR (126 MHz, $CDCl_3$)

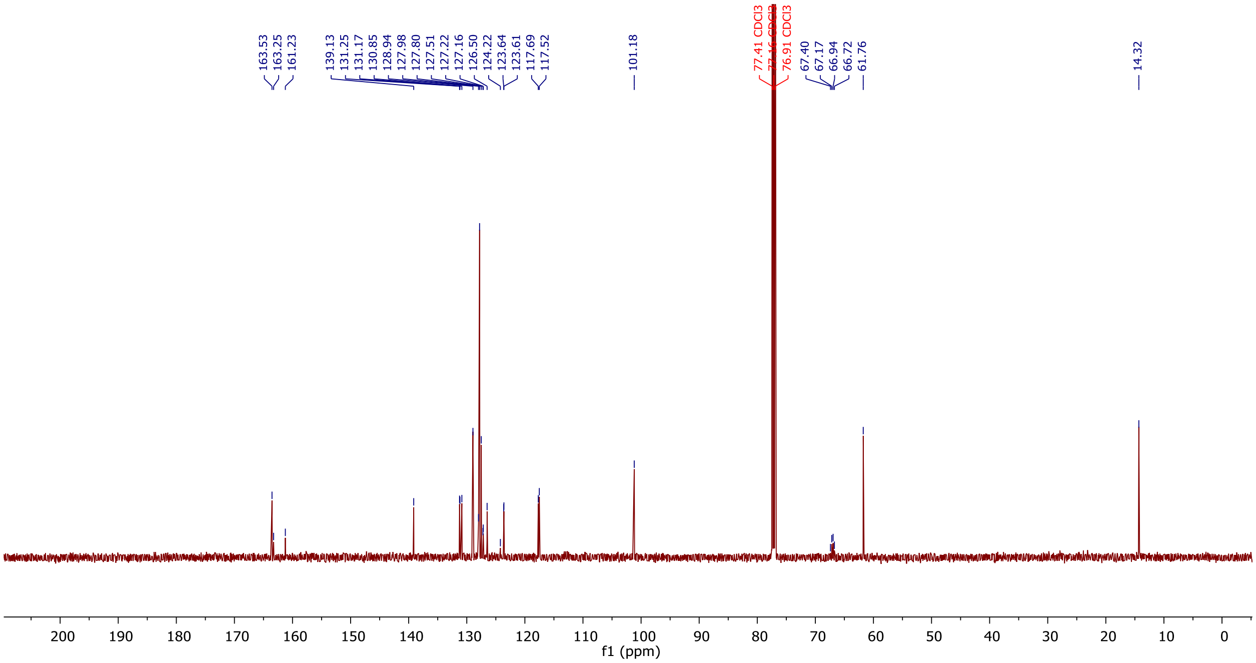

^{19}F NMR (470 MHz, $CDCl_3$)

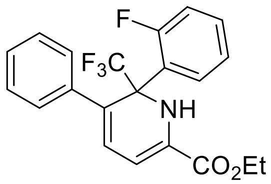
4k

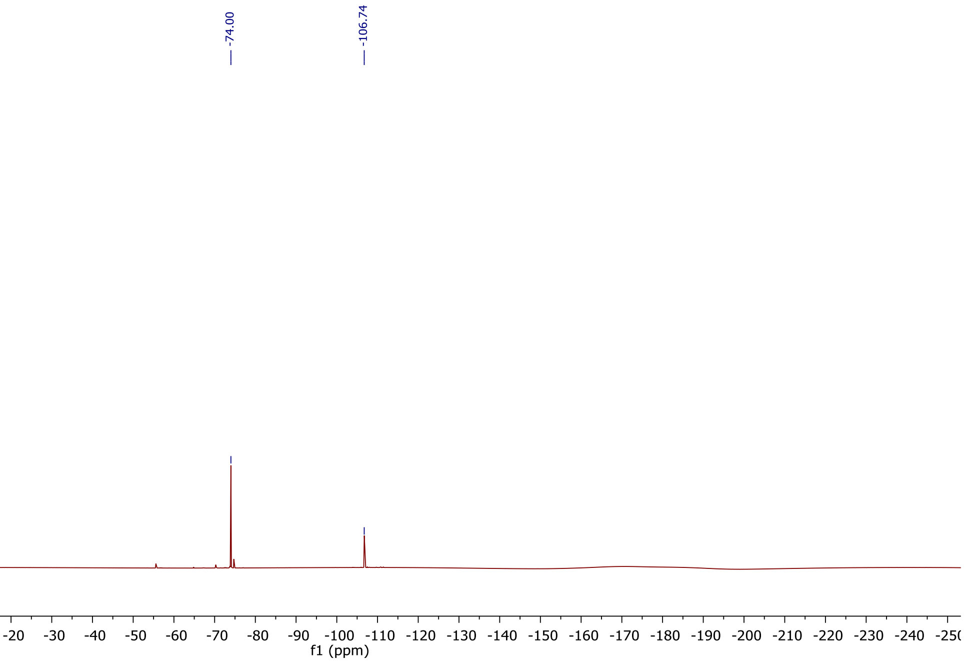

^{1}H NMR (500 MHz, $CDCl_3$)

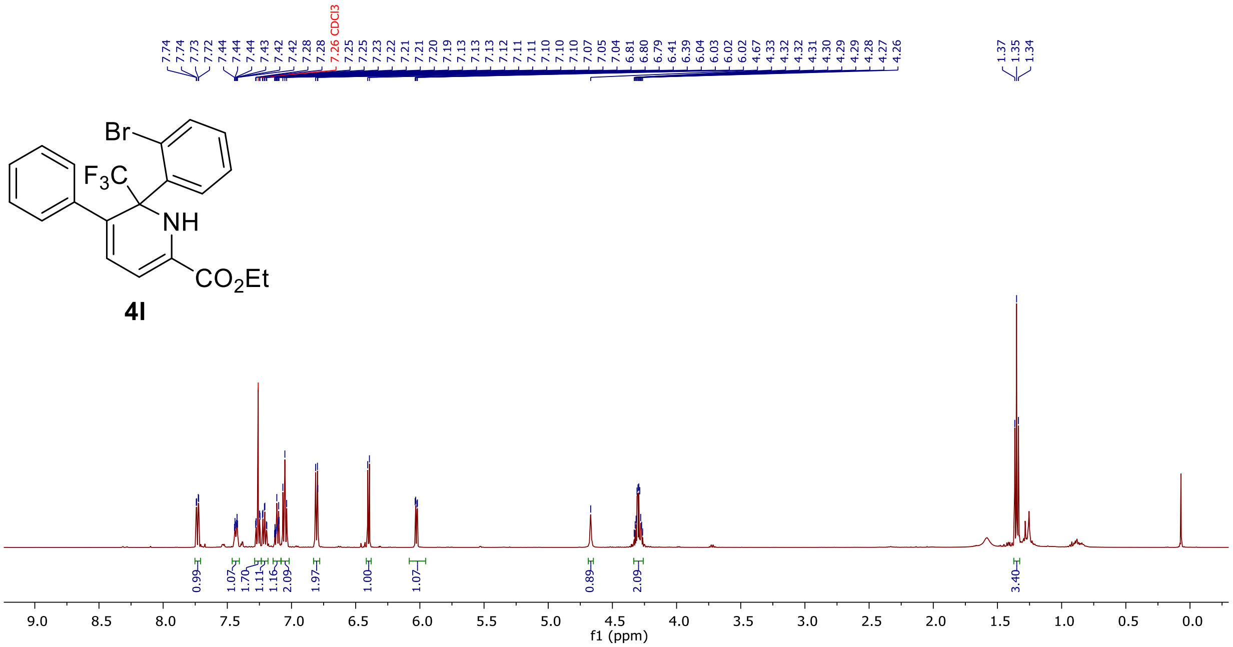

^{13}C NMR (126 MHz, $CDCl_3$)

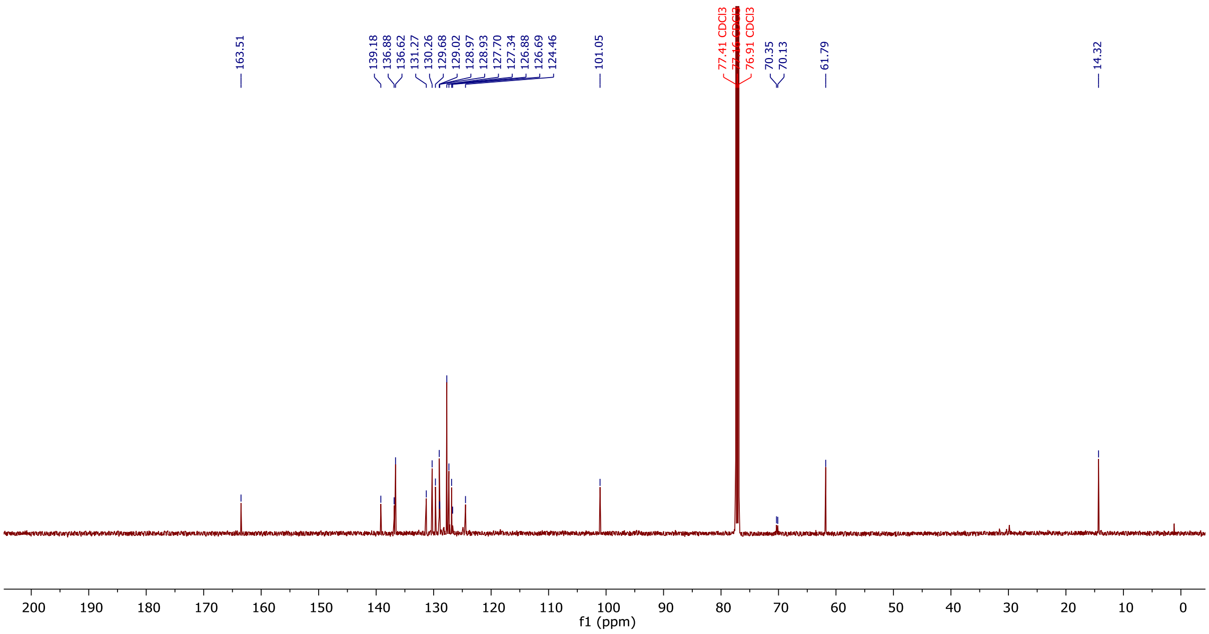

^{19}F NMR (470 MHz, $CDCl_3$)

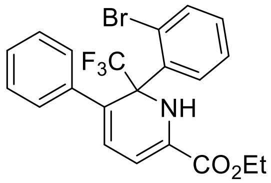

4l

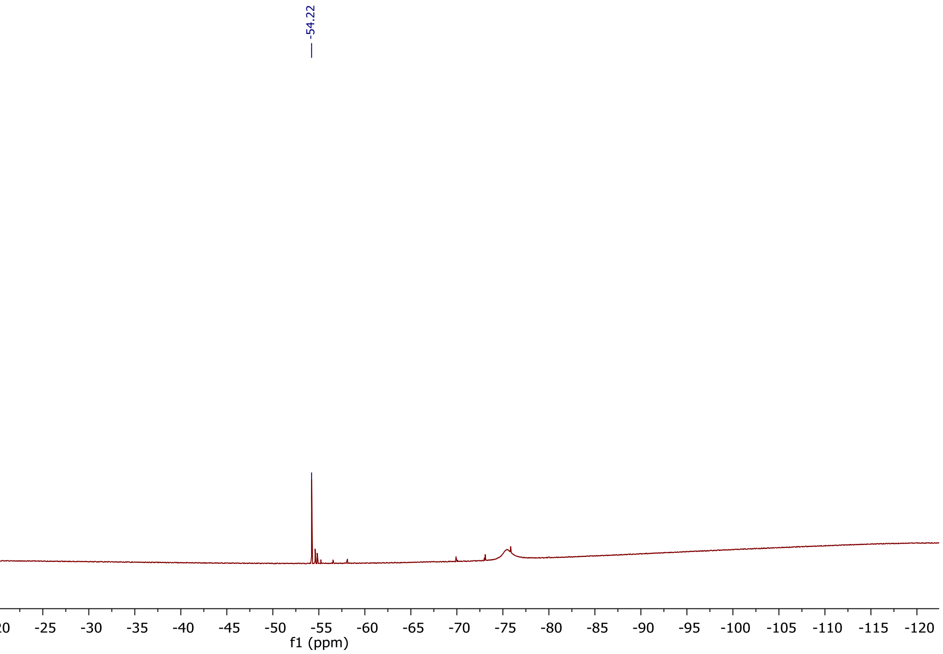

^{1}H NMR (500 MHz, CDCl$_3$)

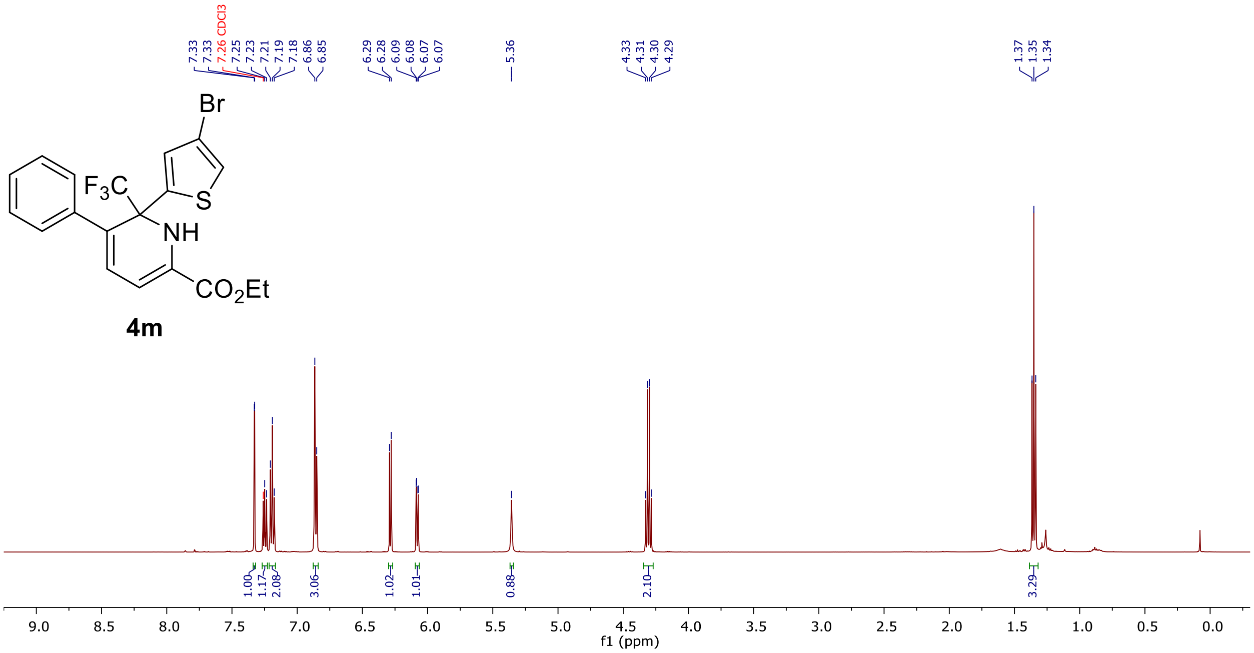

^{13}C NMR (126 MHz, CDCl$_3$)

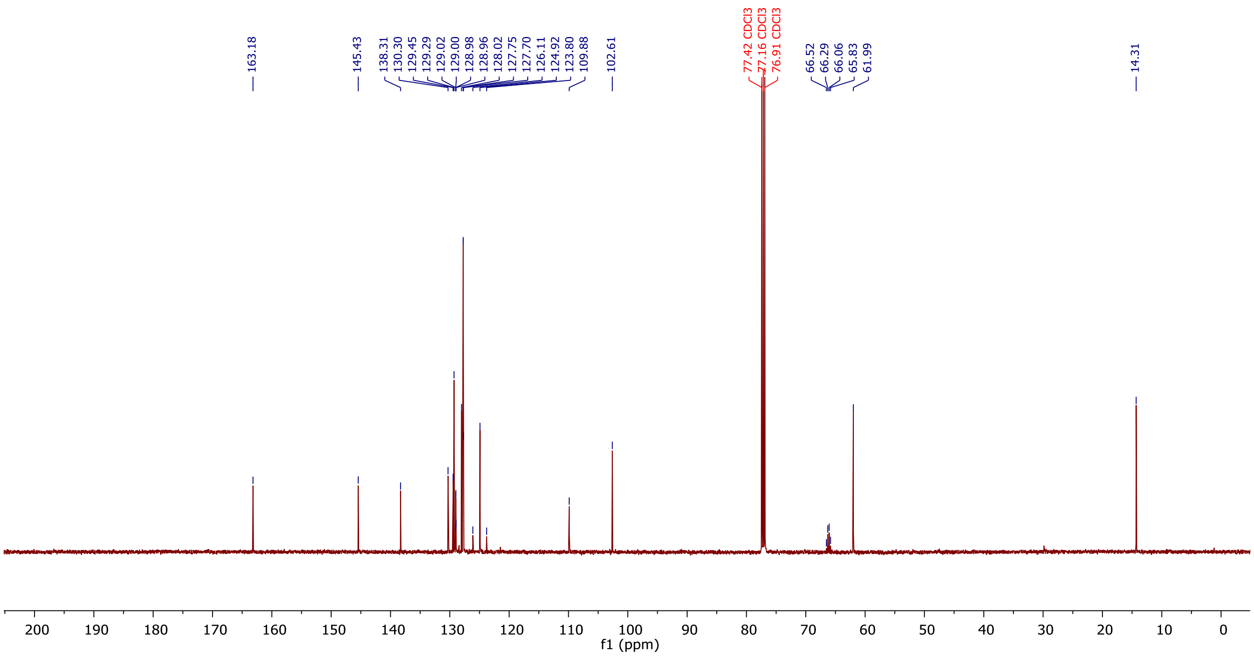

^{19}F NMR (470 MHz, $CDCl_3$)

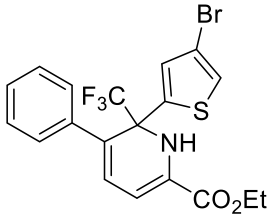

4m

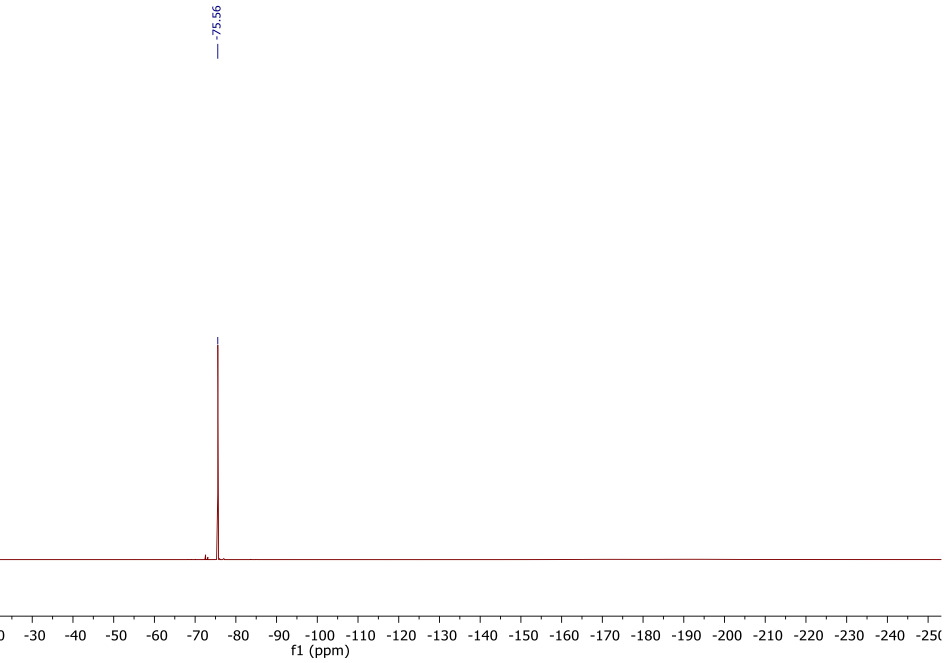

^{1}H NMR (500 MHz, $CDCl_3$)

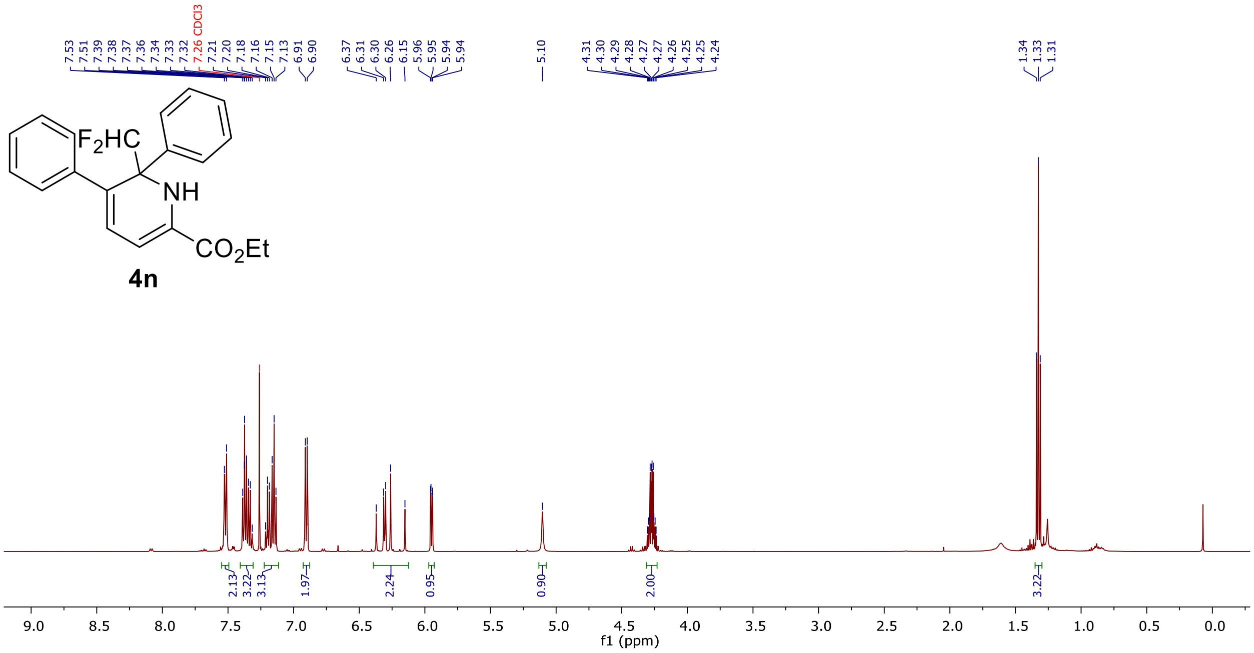

^{13}C NMR (126 MHz, $CDCl_3$)

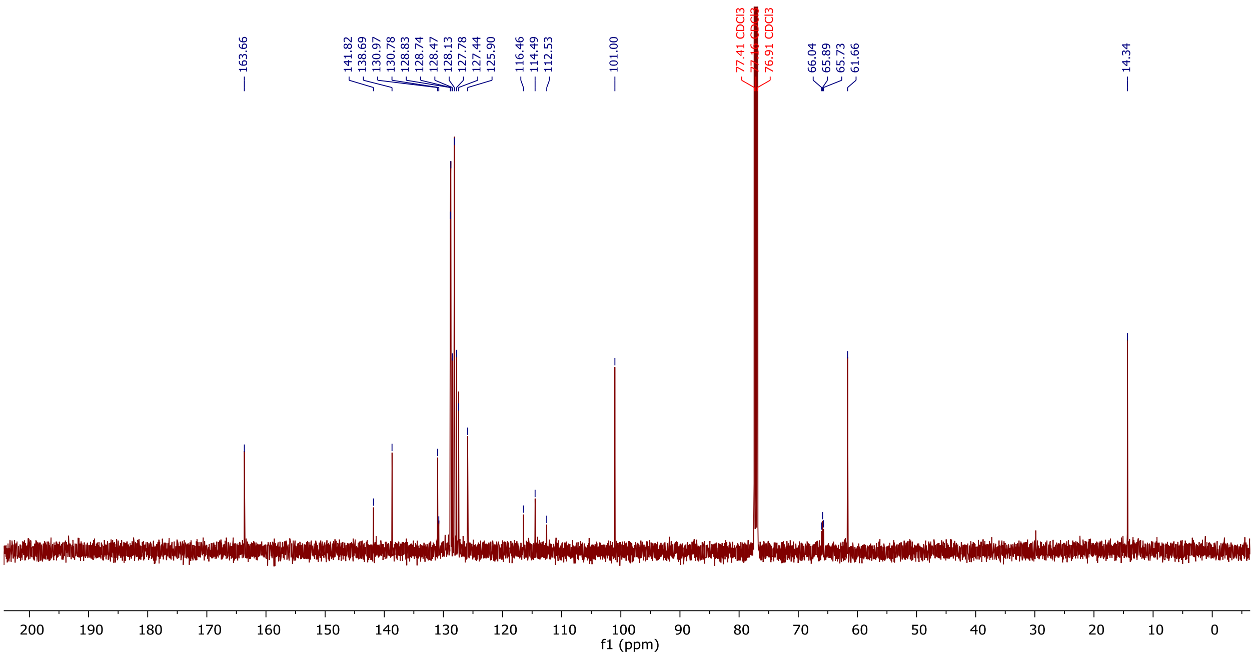

^{19}F NMR (470 MHz, CDCl$_3$)

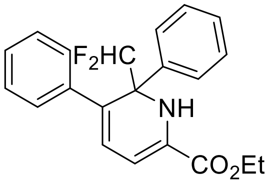

4n

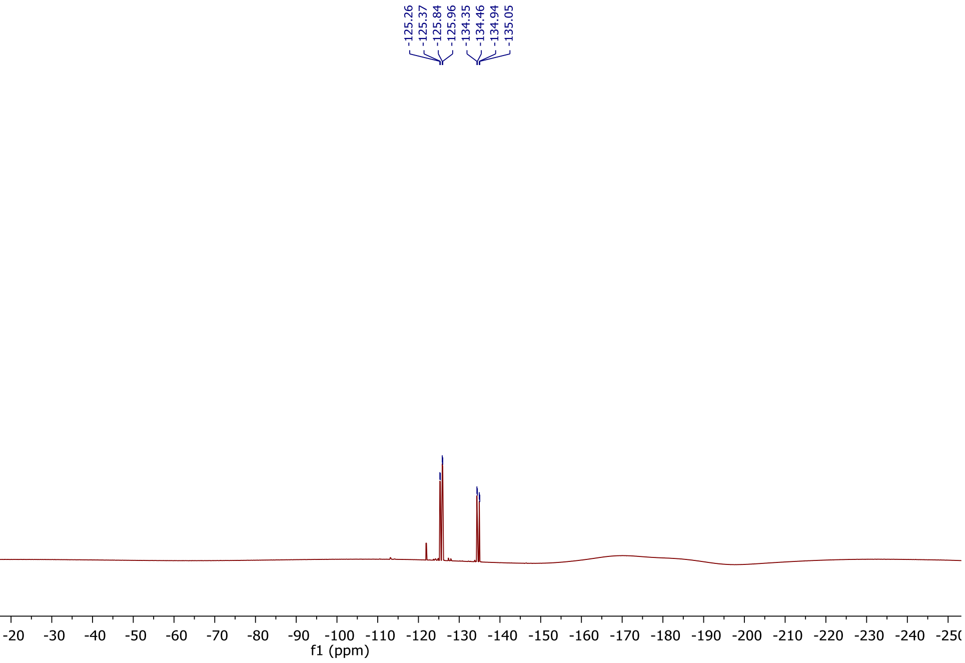

^{1}H NMR (500 MHz, $CDCl_3$)

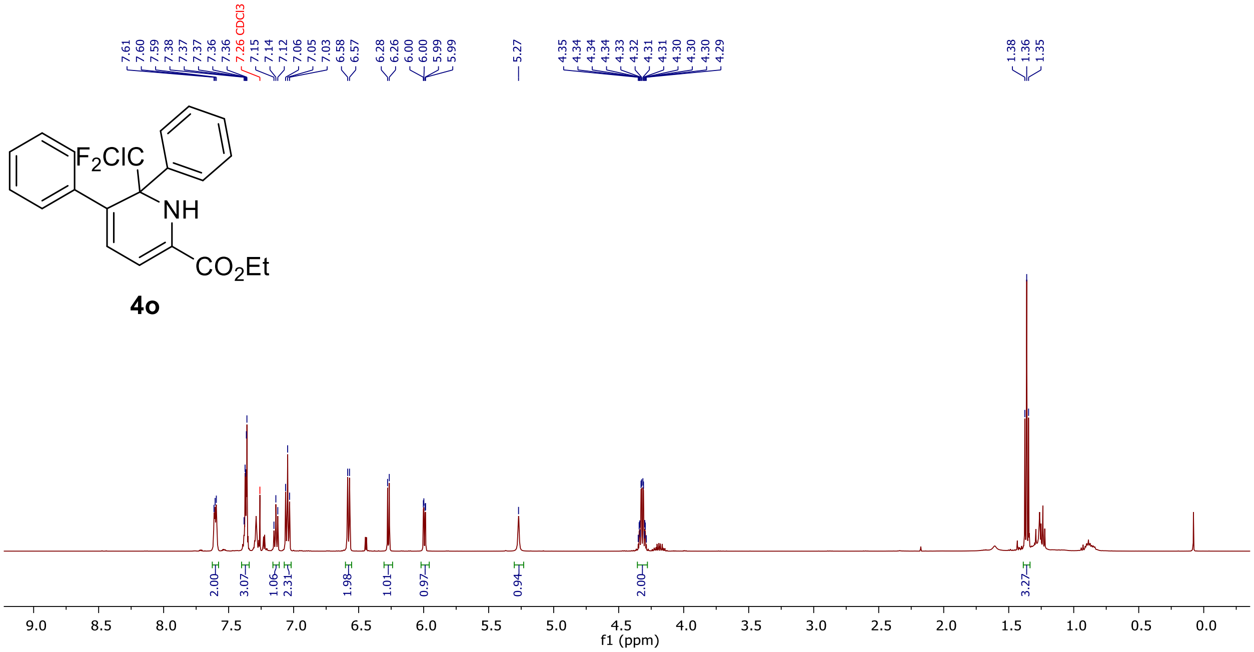

^{13}C NMR (126 MHz, $CDCl_3$)

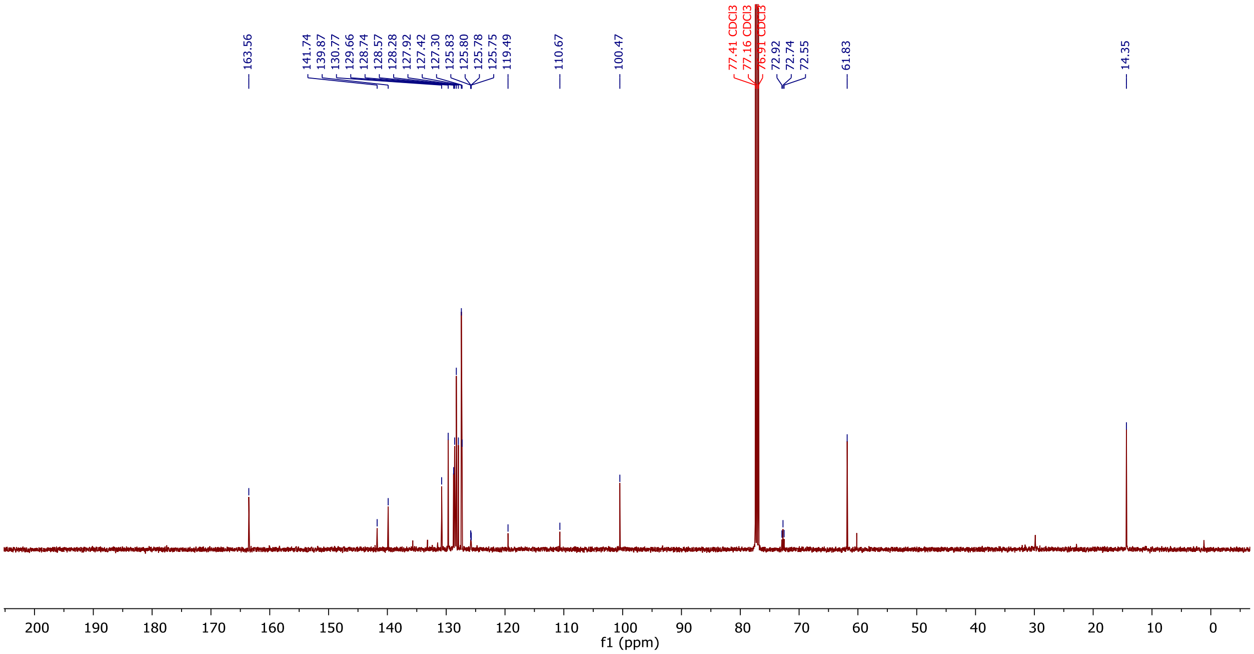

^{19}F NMR (470 MHz, $CDCl_3$)

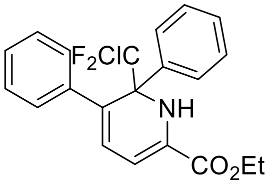

4o

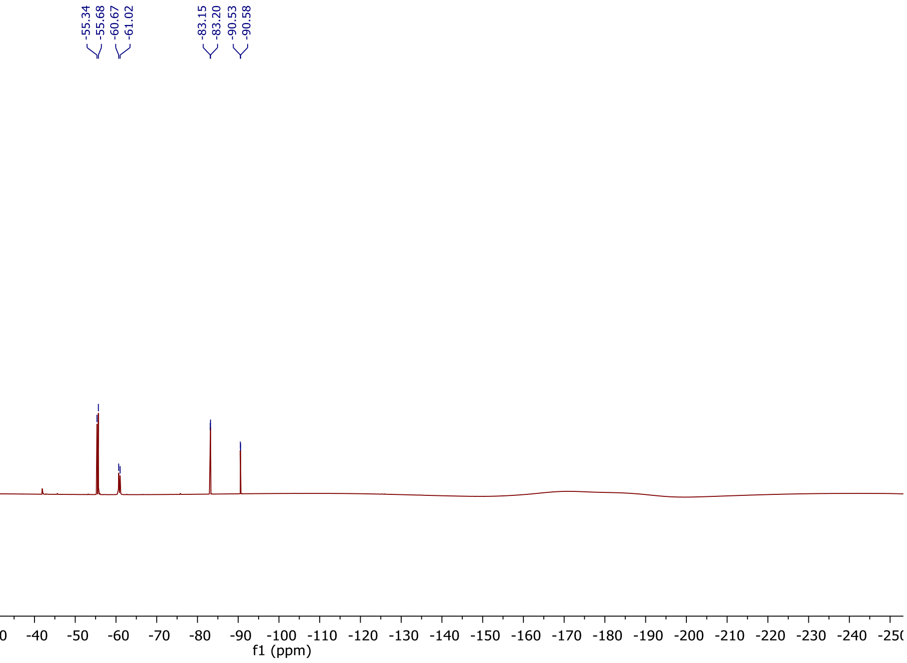

^{1}H NMR (500 MHz, $CDCl_3$)

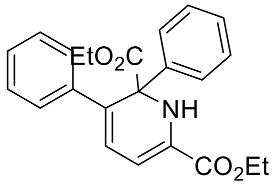

4p

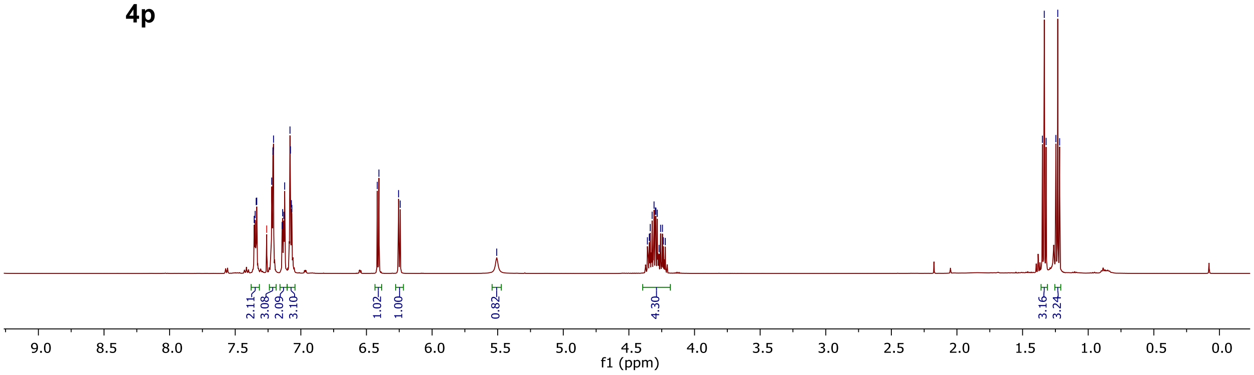

^{13}C NMR (126 MHz, $CDCl_3$)

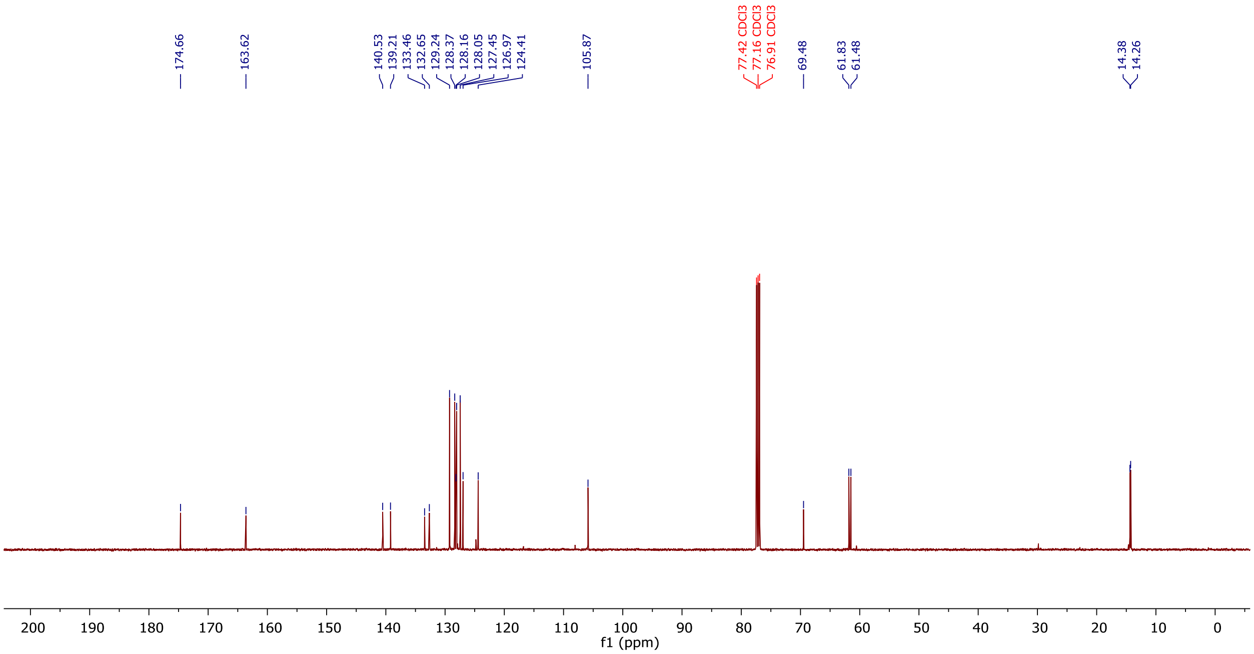

^{1}H NMR (500 MHz, $CDCl_3$)

inseparable mixture with the intermediate acyclic imine, 4:1 ratio of product **4q** vs. intermediate

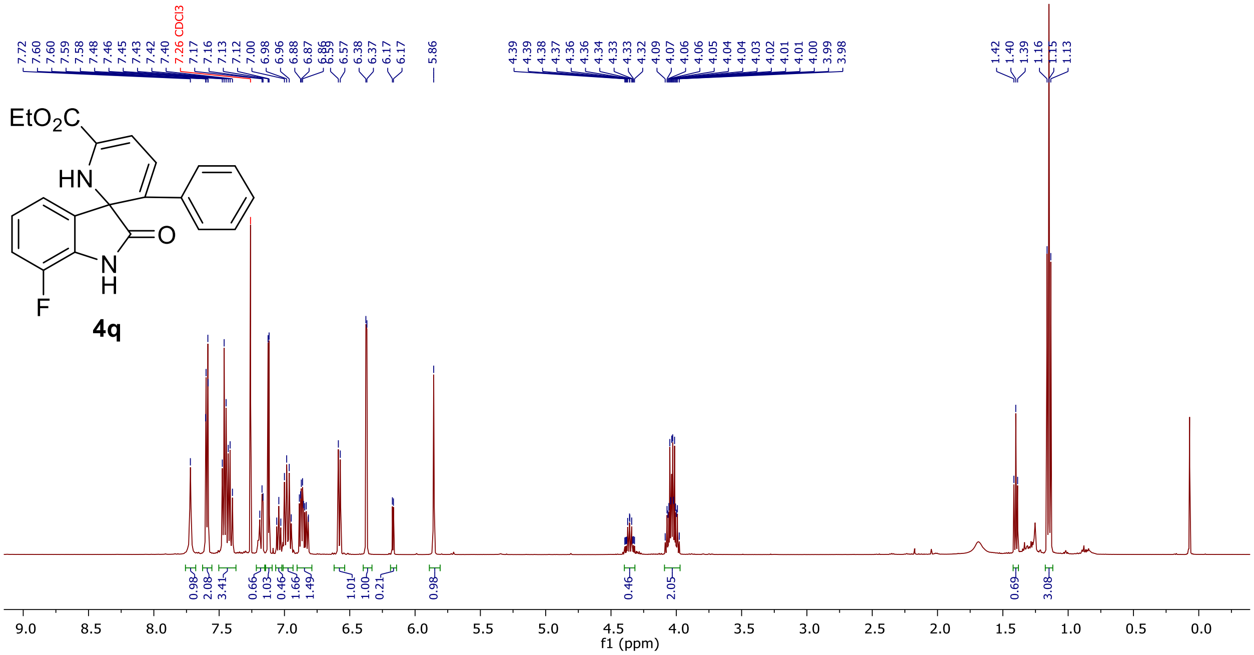

^{13}C NMR (126 MHz, $CDCl_3$)

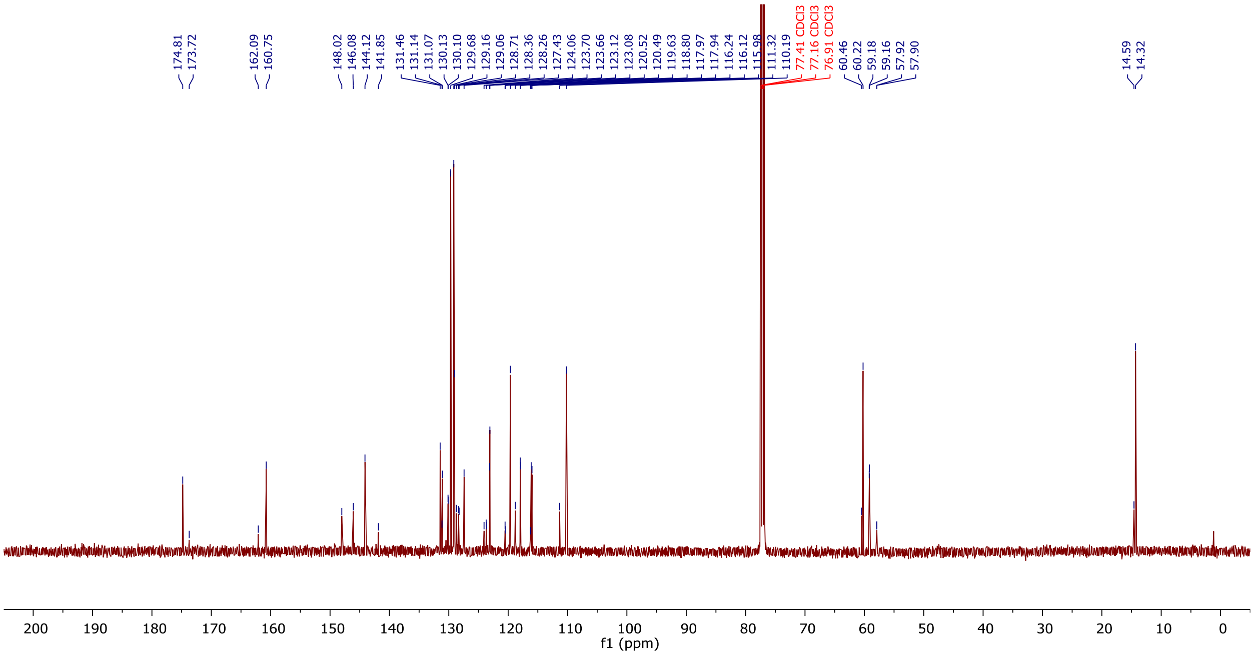

^{19}F NMR (470 MHz, $CDCl_3$)

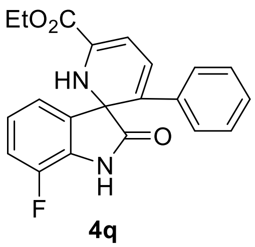

4q

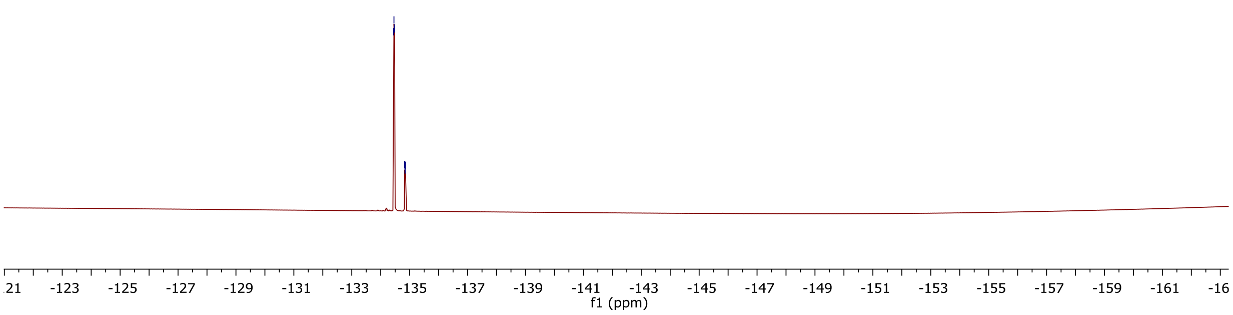

^{1}H NMR (500 MHz, $CDCl_3$)

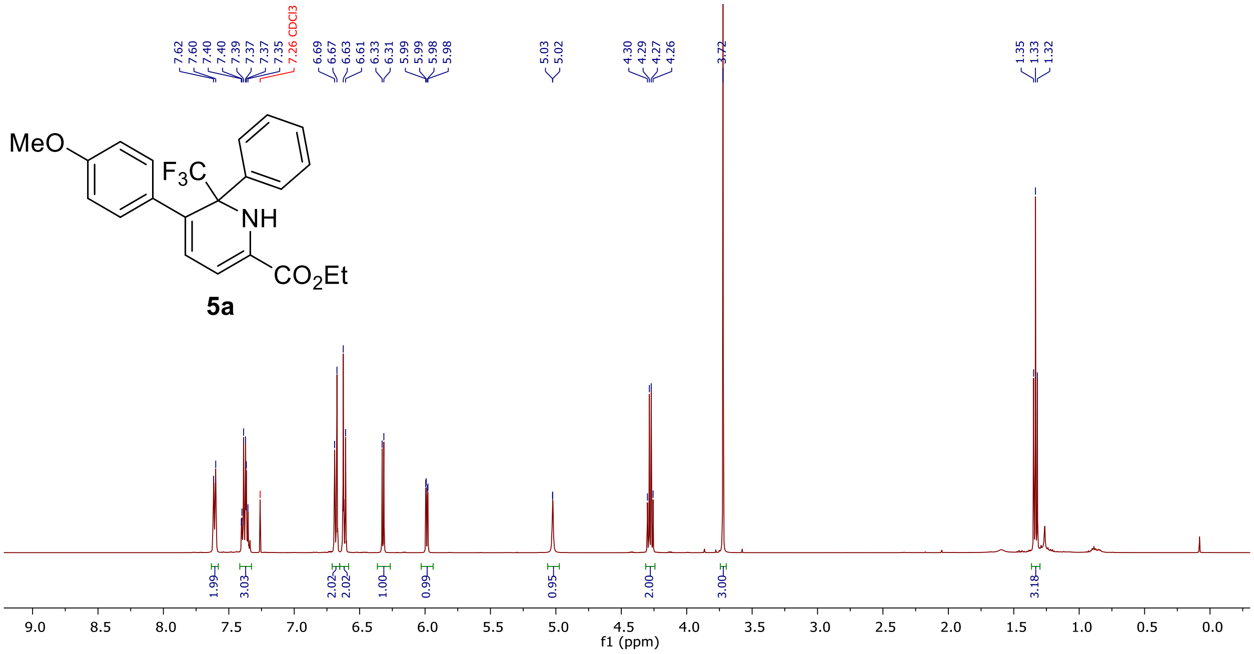

^{13}C NMR (126 MHz, $CDCl_3$)

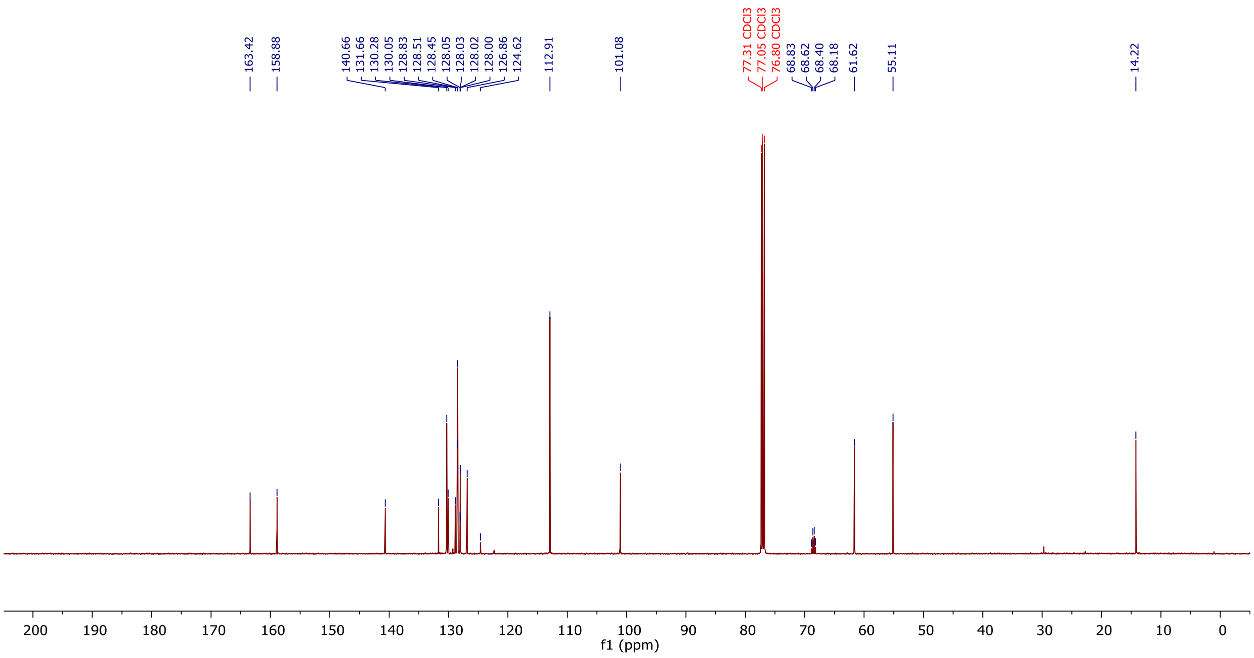

^{19}F NMR (470 MHz, $CDCl_3$)

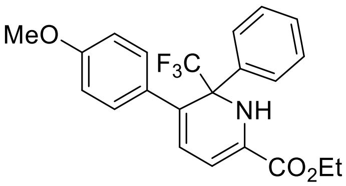

5a

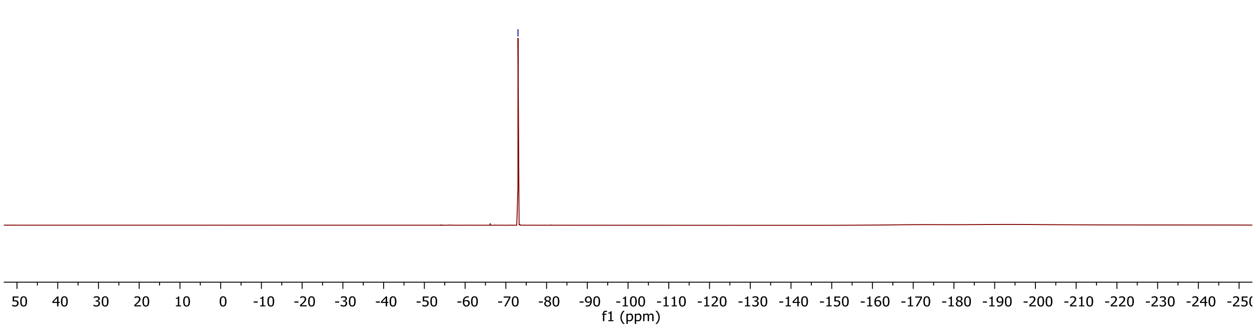

^{1}H NMR (500 MHz, $CDCl_3$)

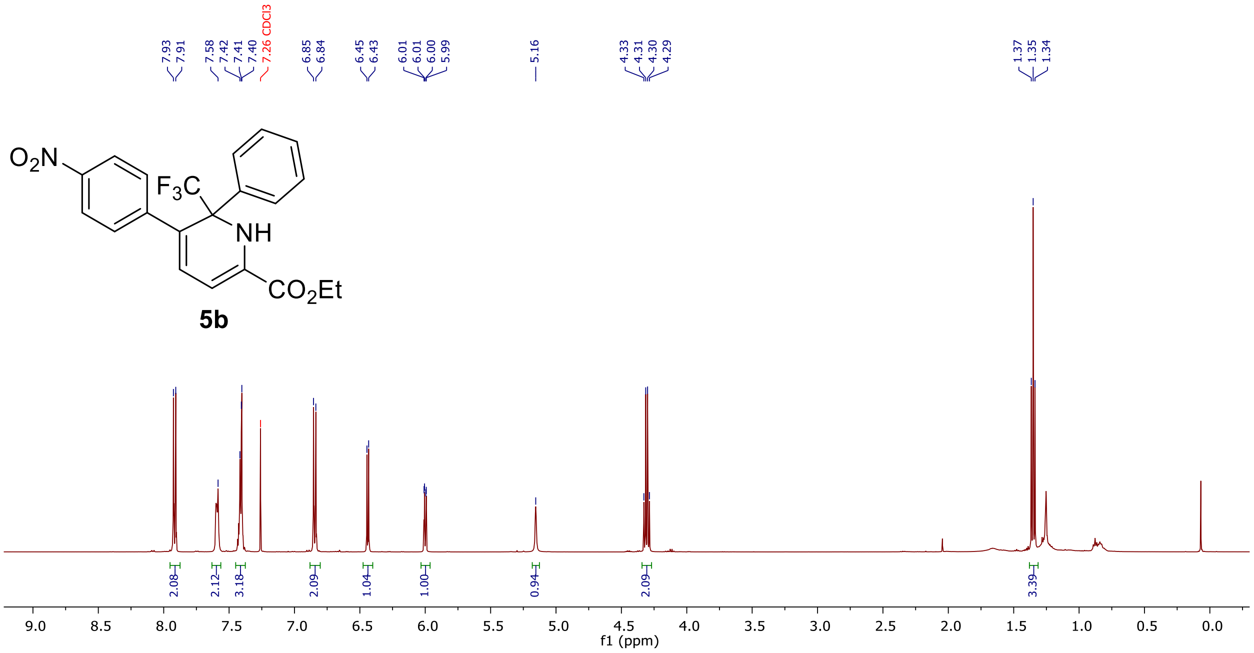

^{13}C NMR (126 MHz, $CDCl_3$)

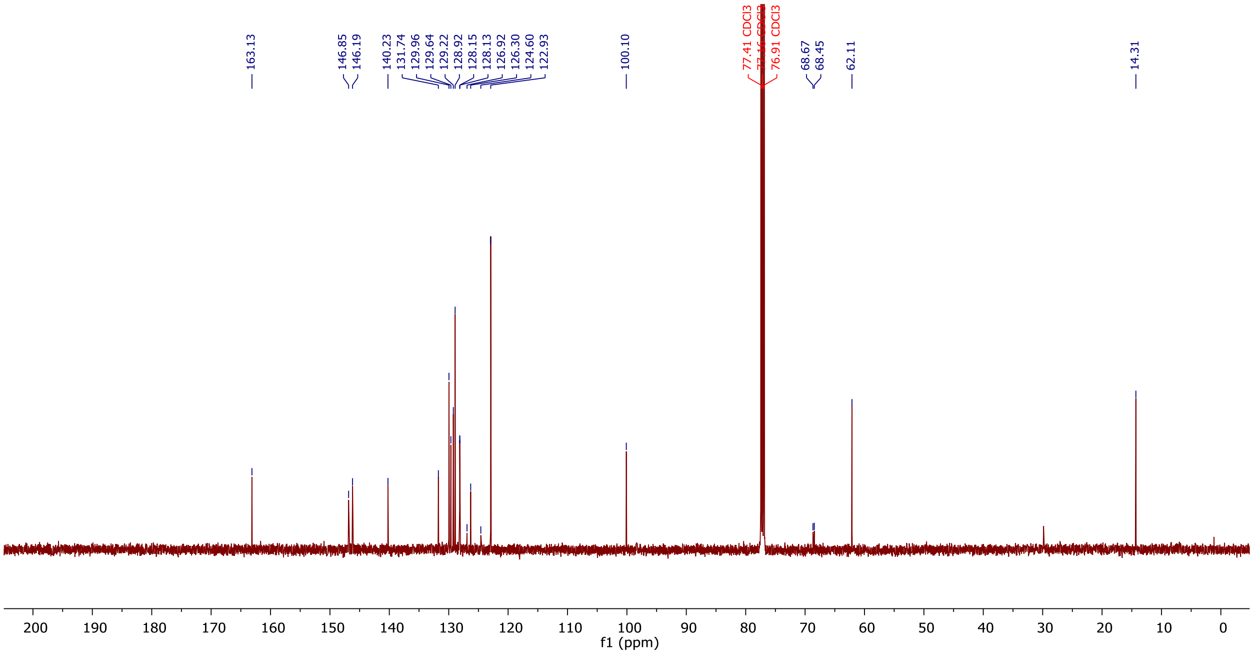

^{19}F NMR (470 MHz, $CDCl_3$)

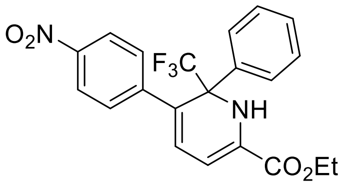

5b

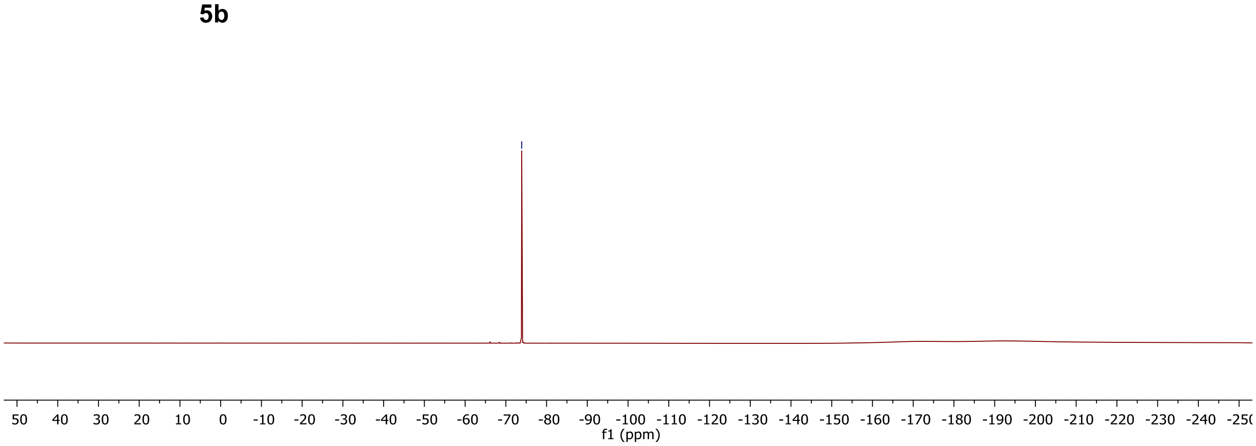

^{1}H NMR (500 MHz, $CDCl_3$)

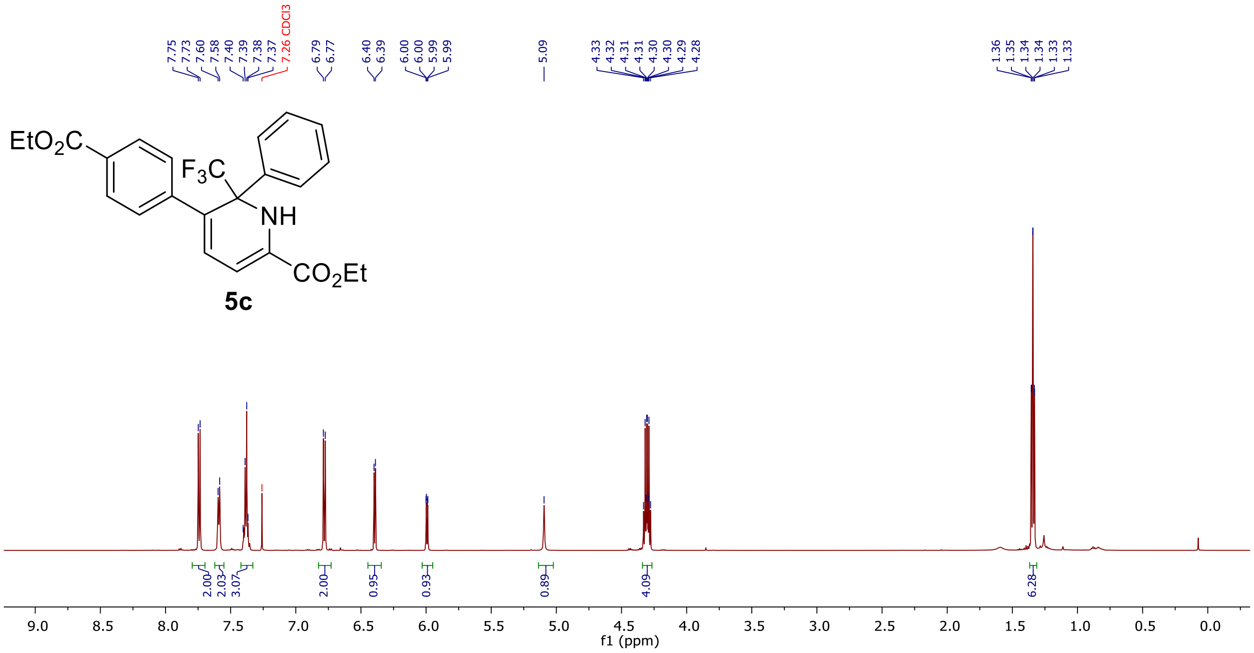

^{13}C NMR (126 MHz, $CDCl_3$)

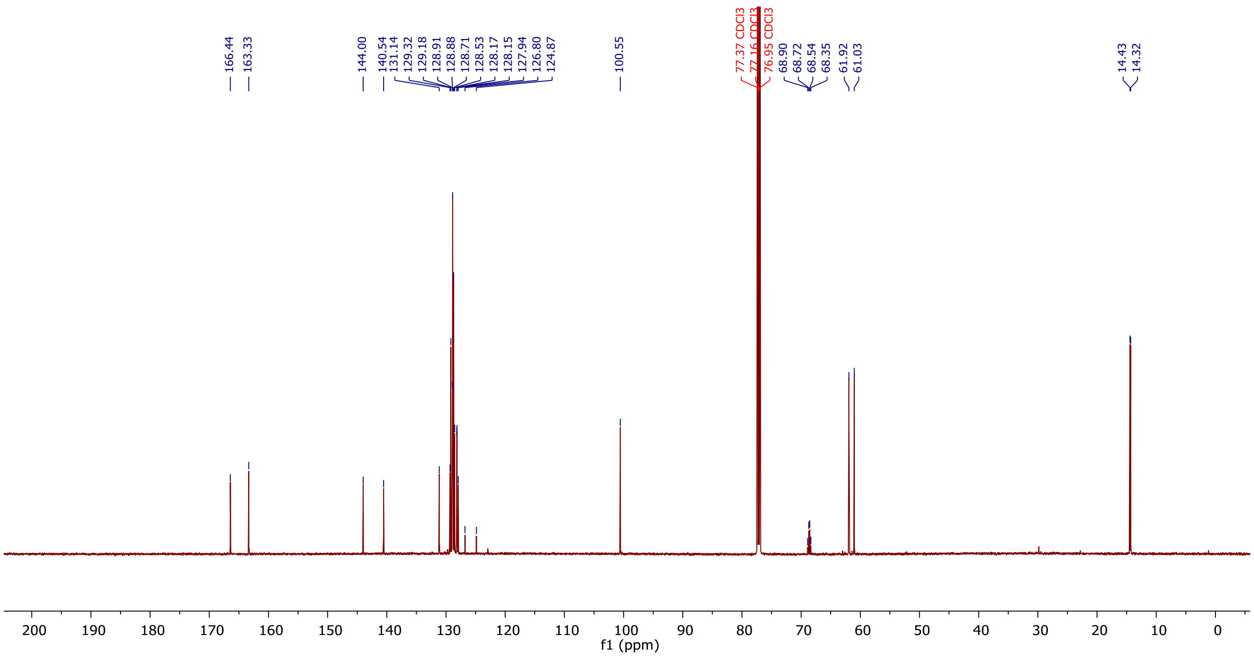

^{19}F NMR (470 MHz, $CDCl_3$)

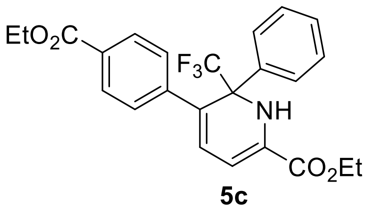

5c

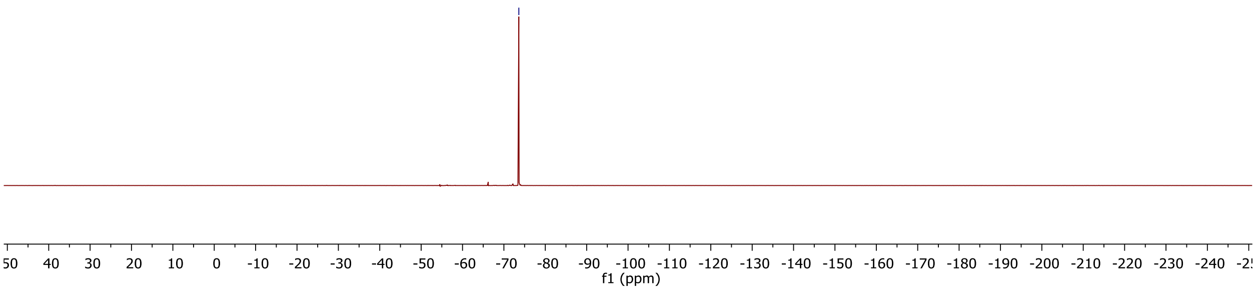

^{1}H NMR (500 MHz, $CDCl_3$)

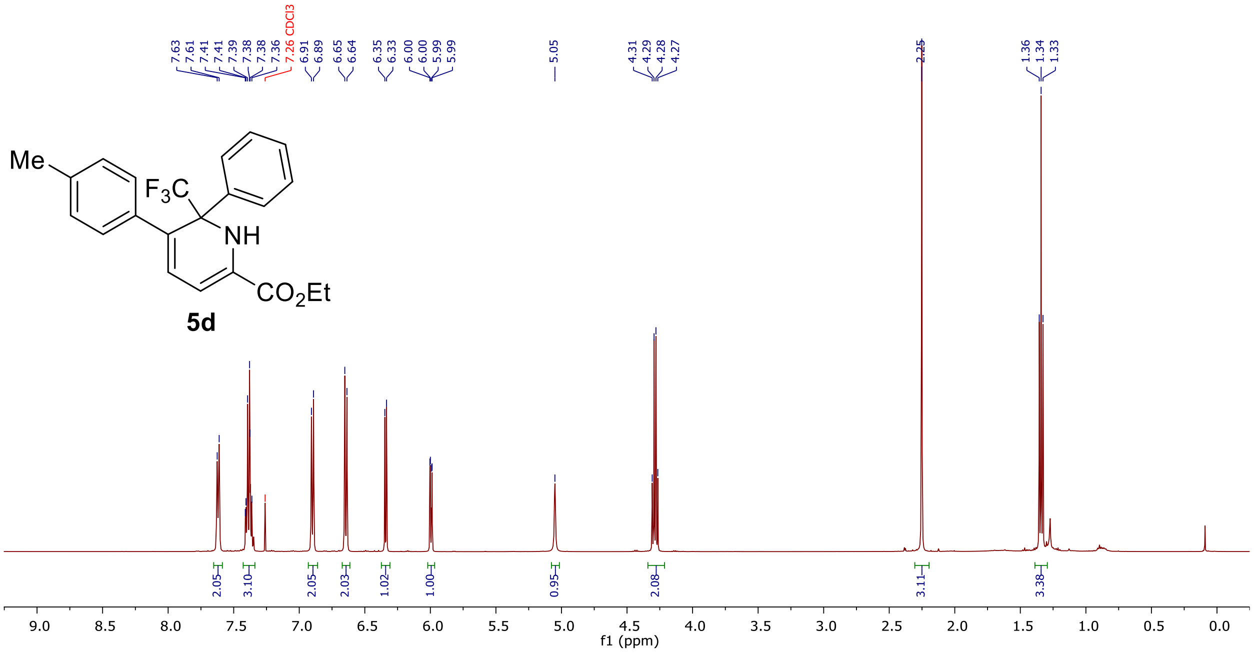

^{13}C NMR (126 MHz, $CDCl_3$)

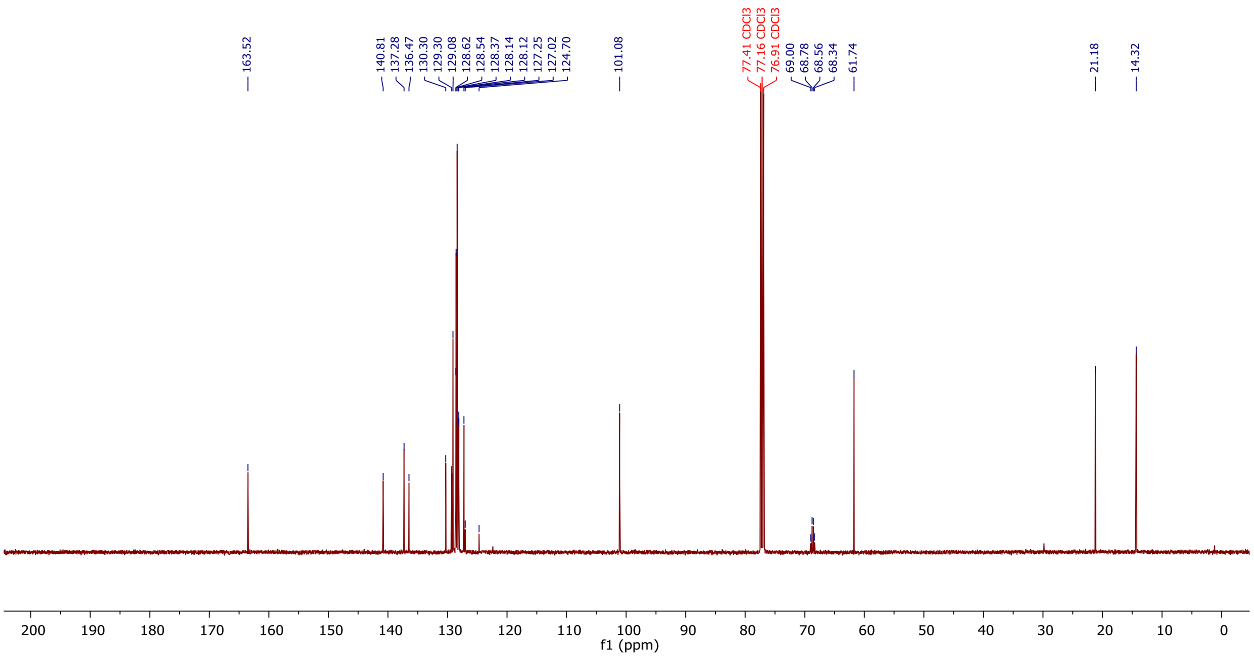

^{19}F NMR (470 MHz, $CDCl_3$)

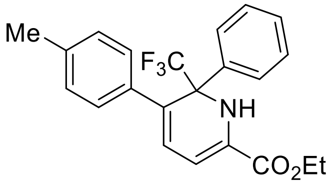

5d

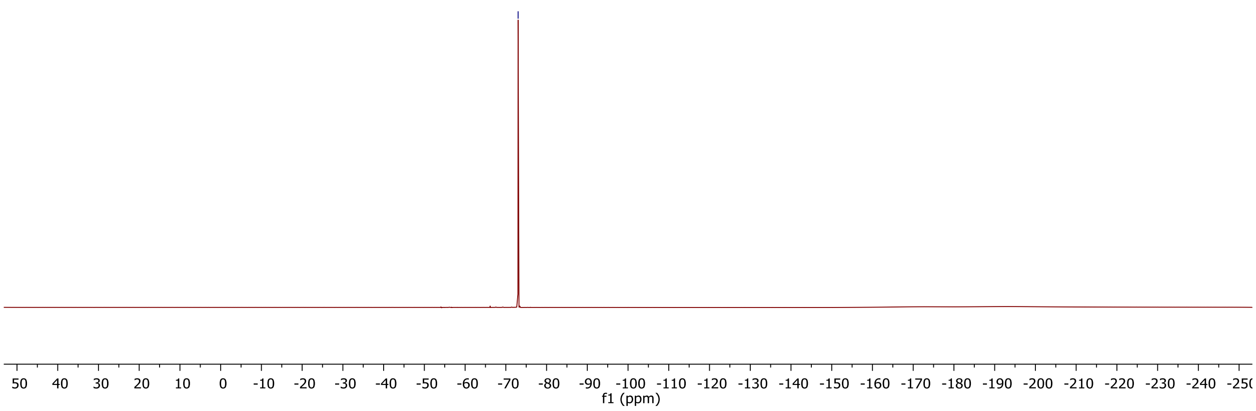

^{1}H NMR (500 MHz, $CDCl_3$)

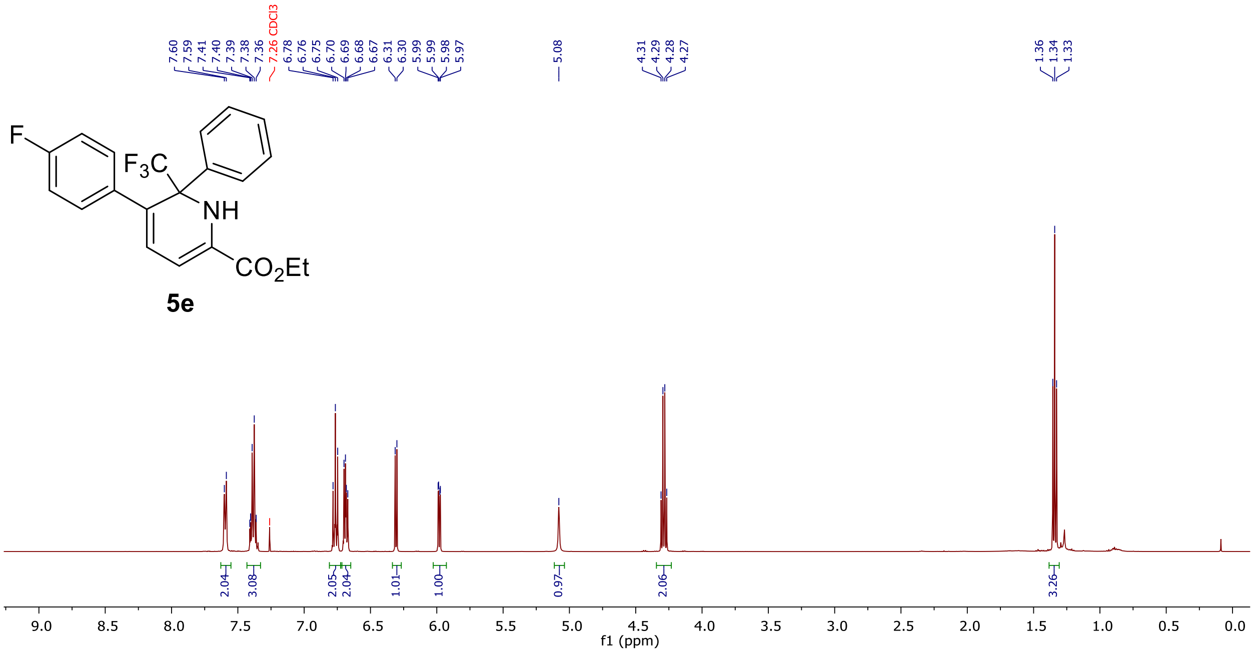

^{13}C NMR (126 MHz, $CDCl_3$)

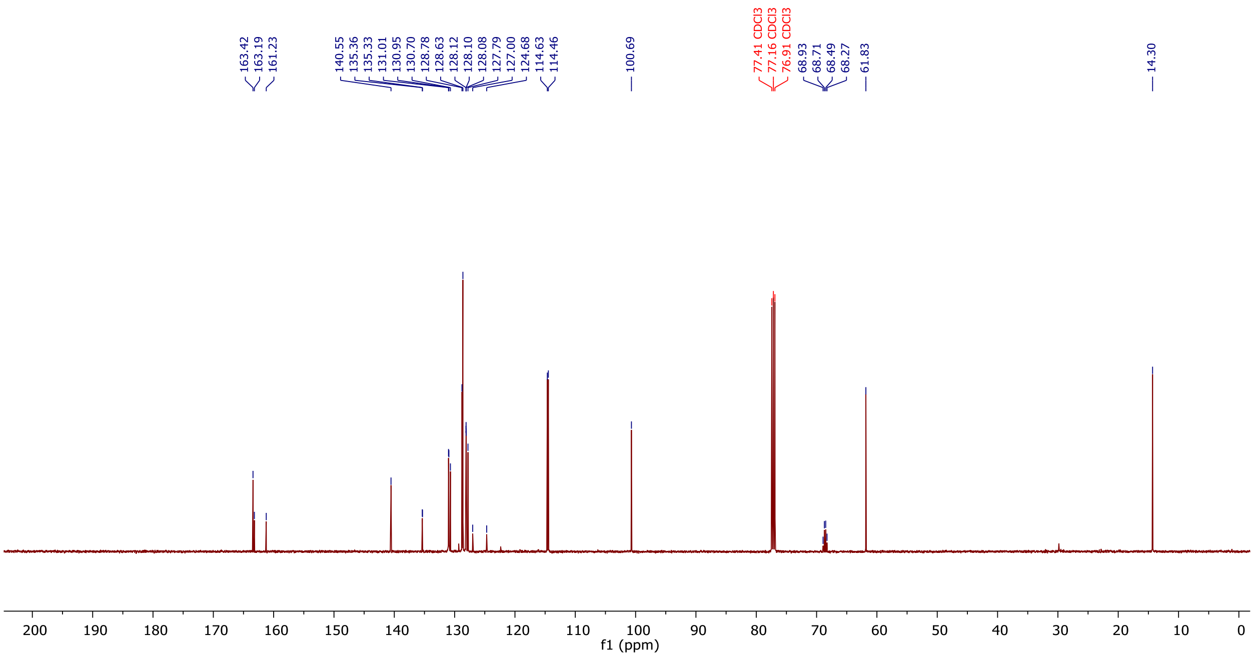

^{19}F NMR (470 MHz, $CDCl_3$)

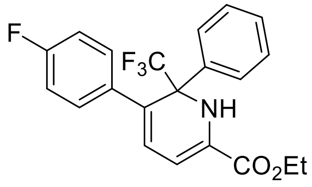

5e

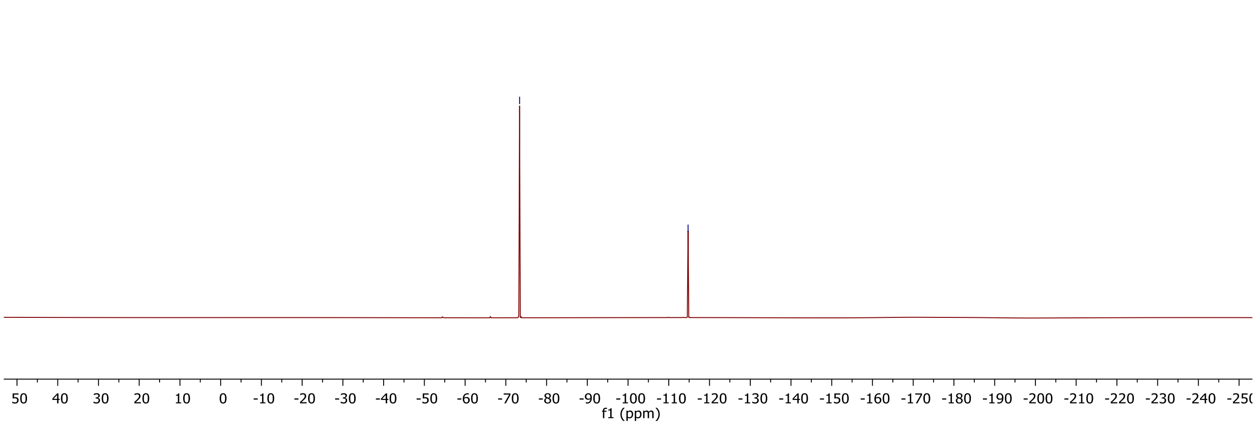

^{1}H NMR (500 MHz, $CDCl_3$)

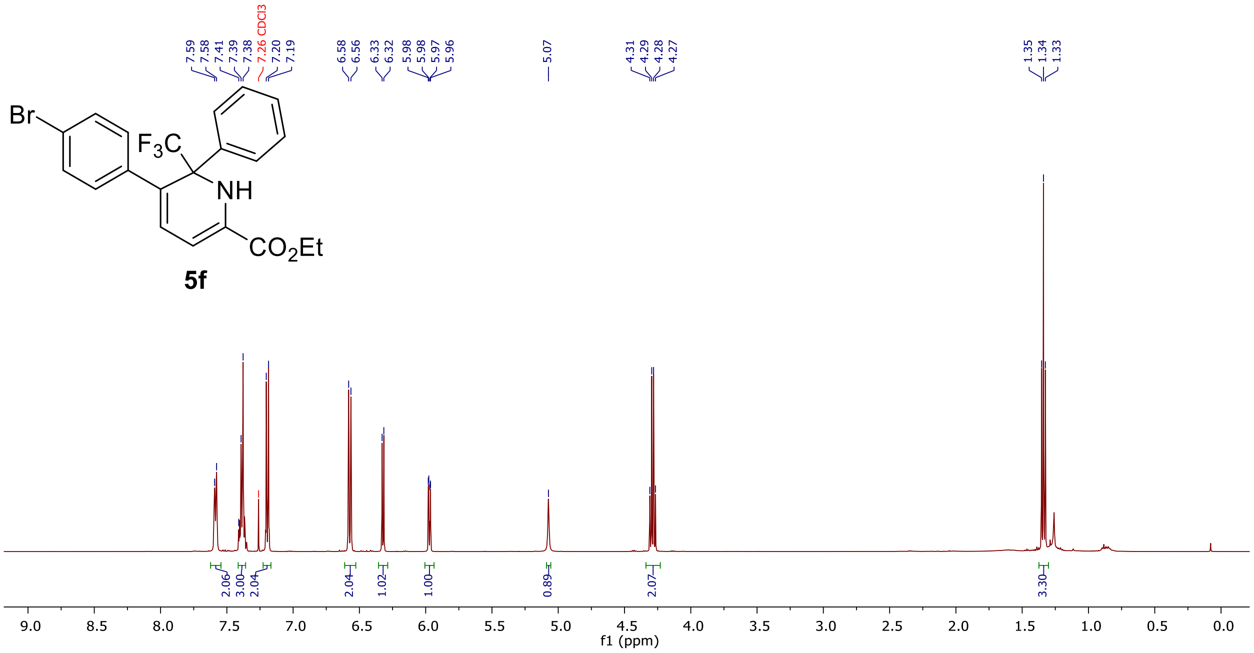

^{13}C NMR (126 MHz, $CDCl_3$)

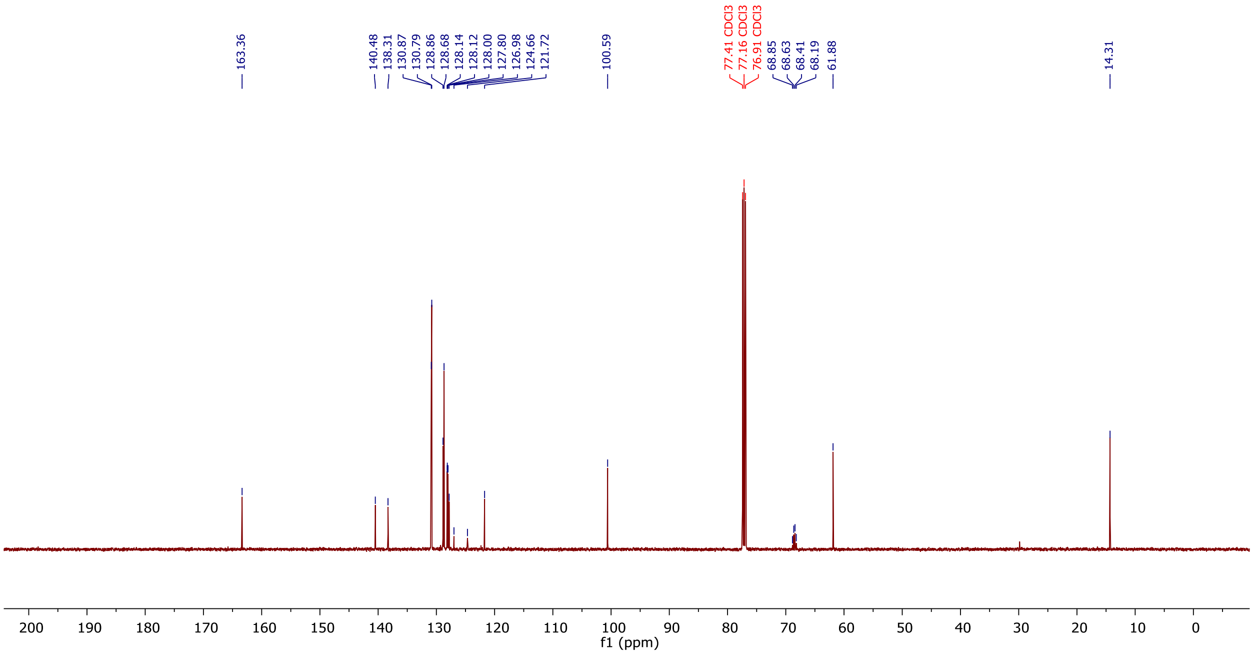

^{19}F NMR (470 MHz, $CDCl_3$)

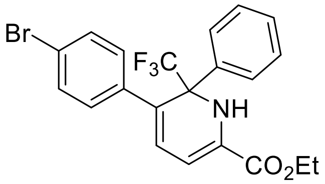

5f

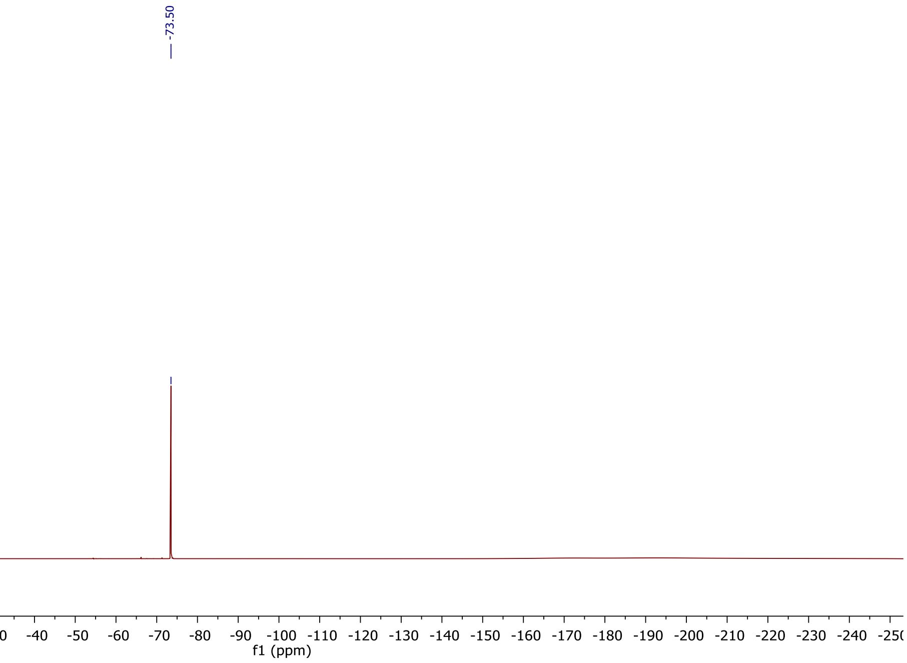

^1H NMR (500 MHz, $CDCl_3$)

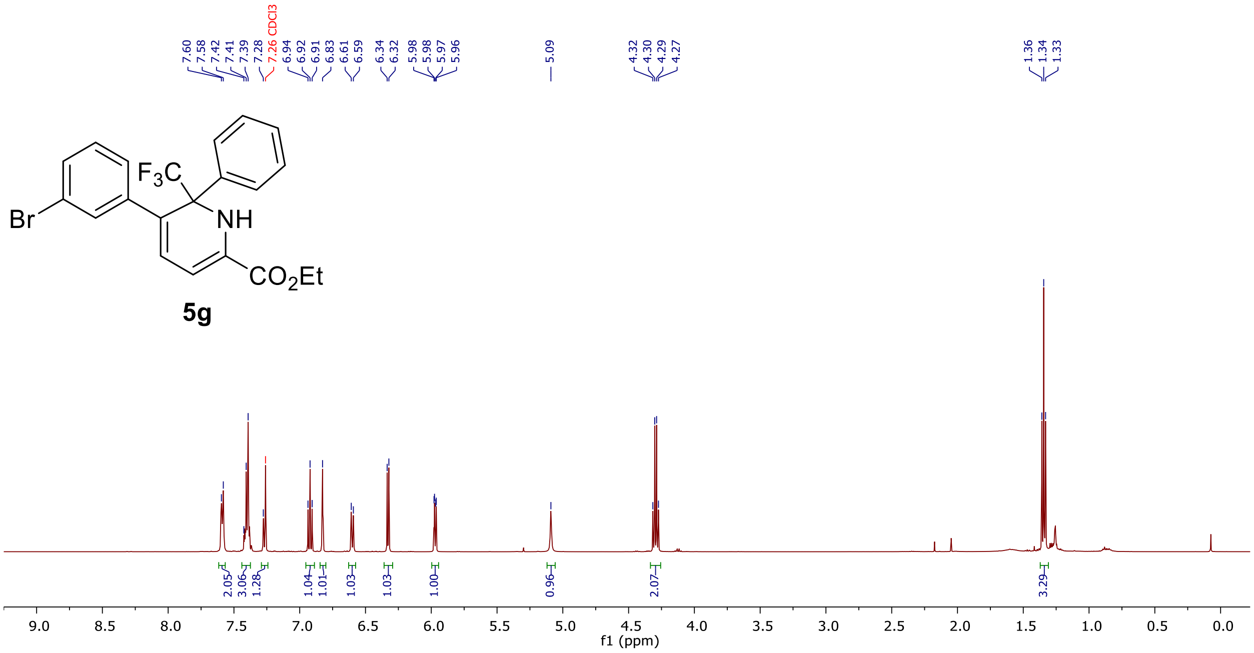

^{13}C NMR (126 MHz, $CDCl_3$)

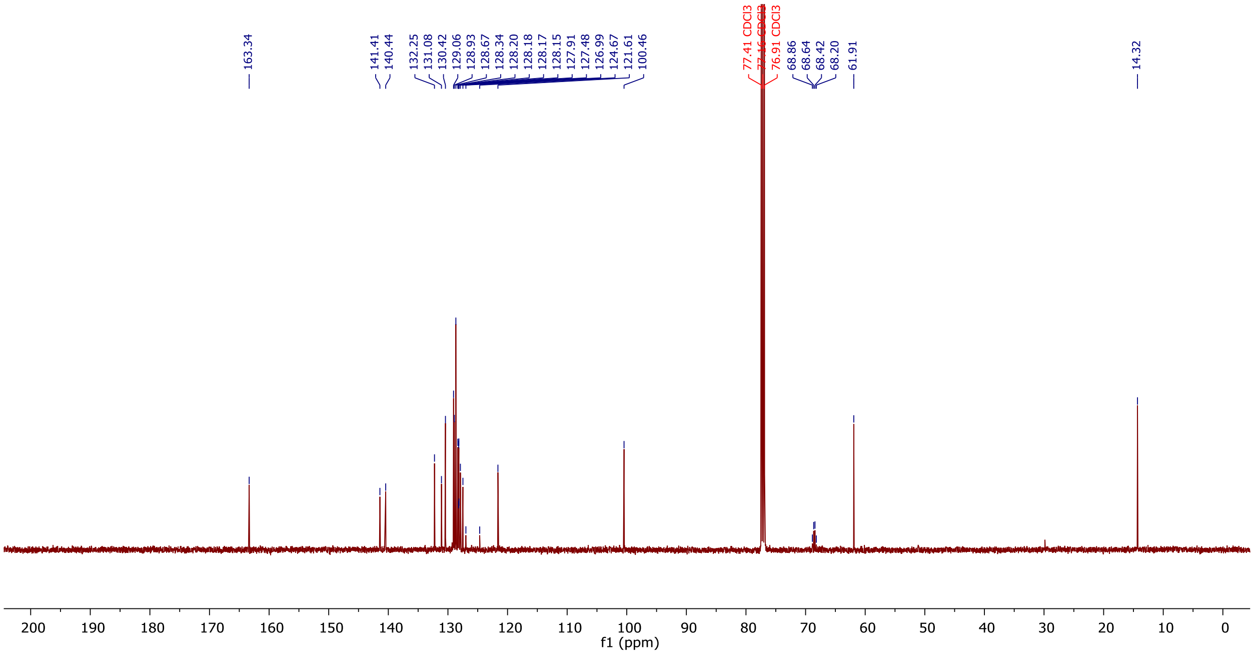

^{19}F NMR (470 MHz, $CDCl_3$)

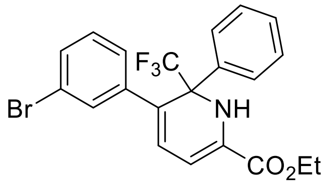

5g

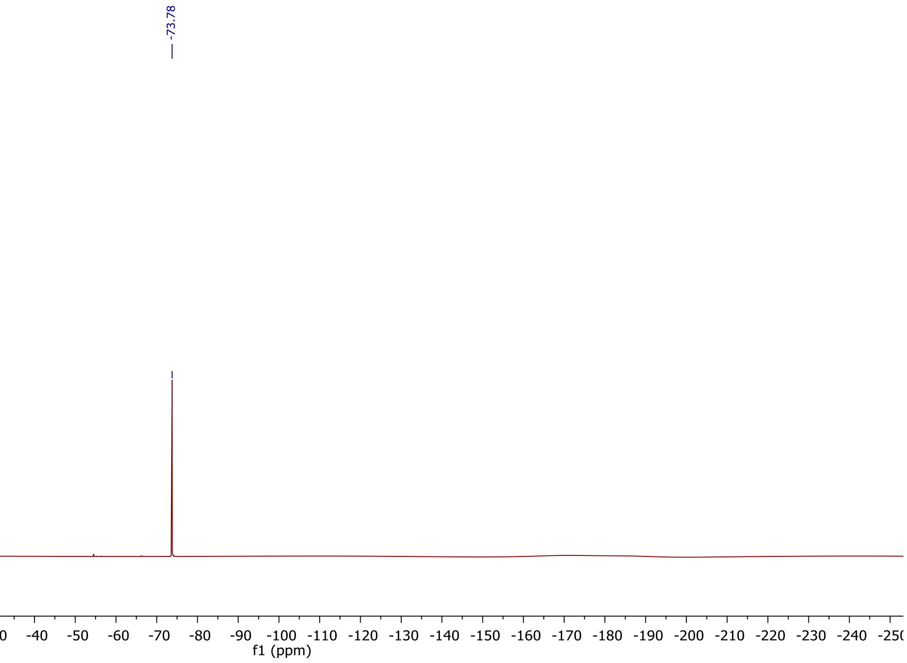

^{1}H NMR (500 MHz, $CDCl_3$)

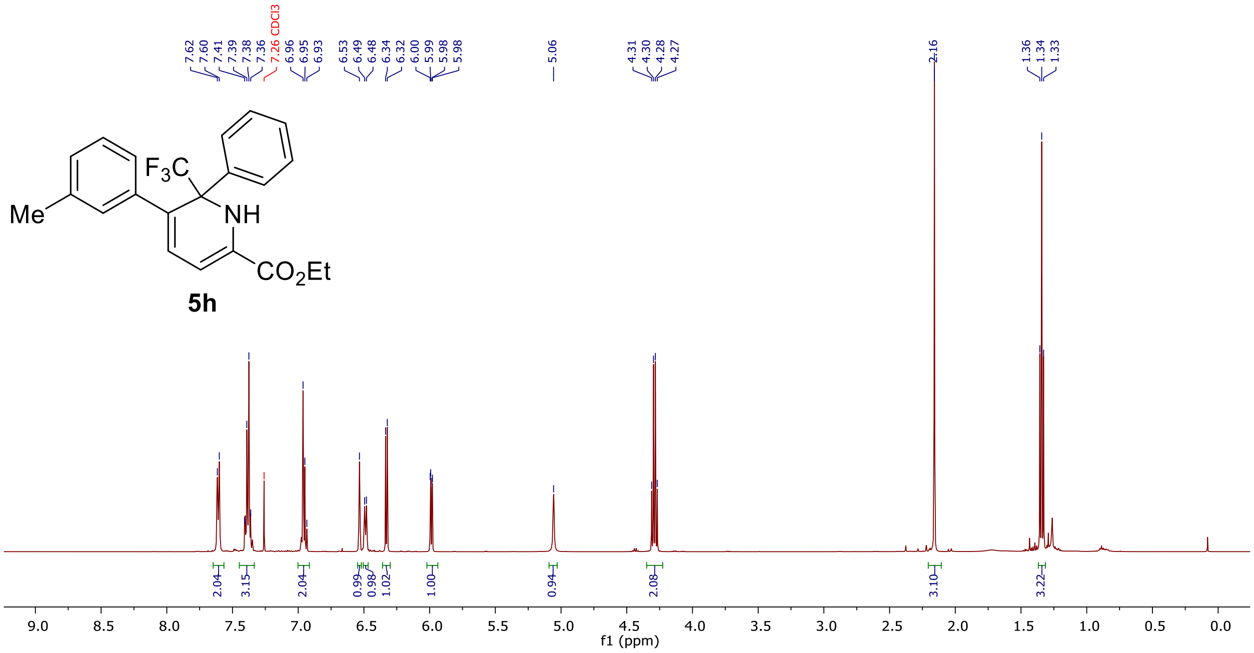

^{13}C NMR (126 MHz, $CDCl_3$)

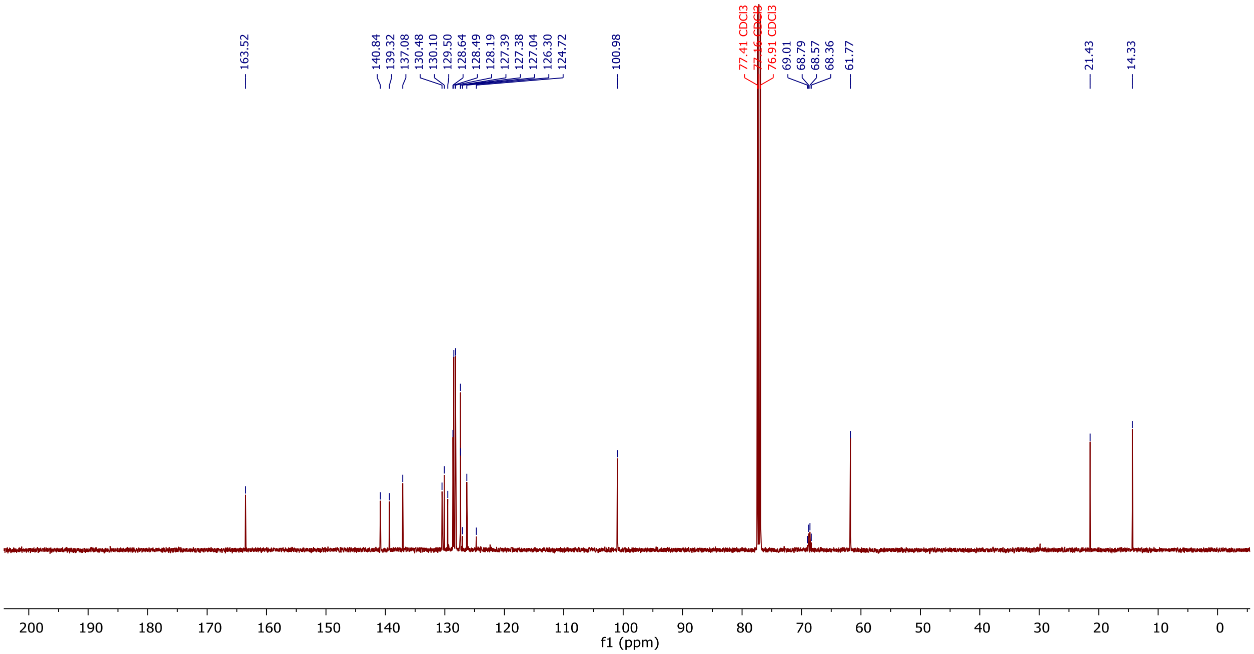

^{19}F NMR (470 MHz, $CDCl_3$)

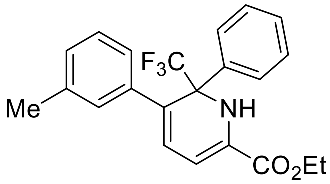

5h

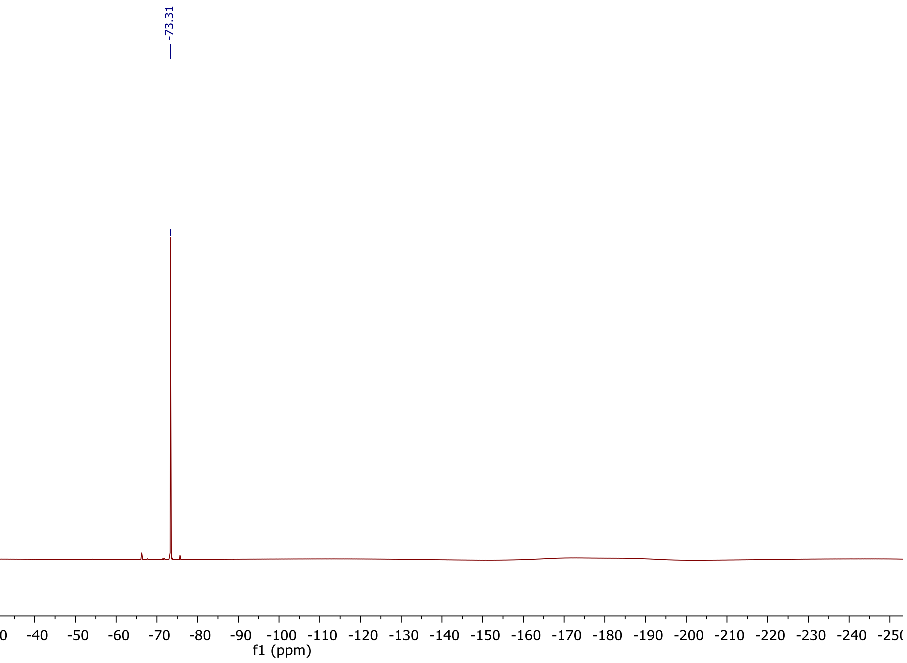

^{1}H NMR (500 MHz, $CDCl_3$)

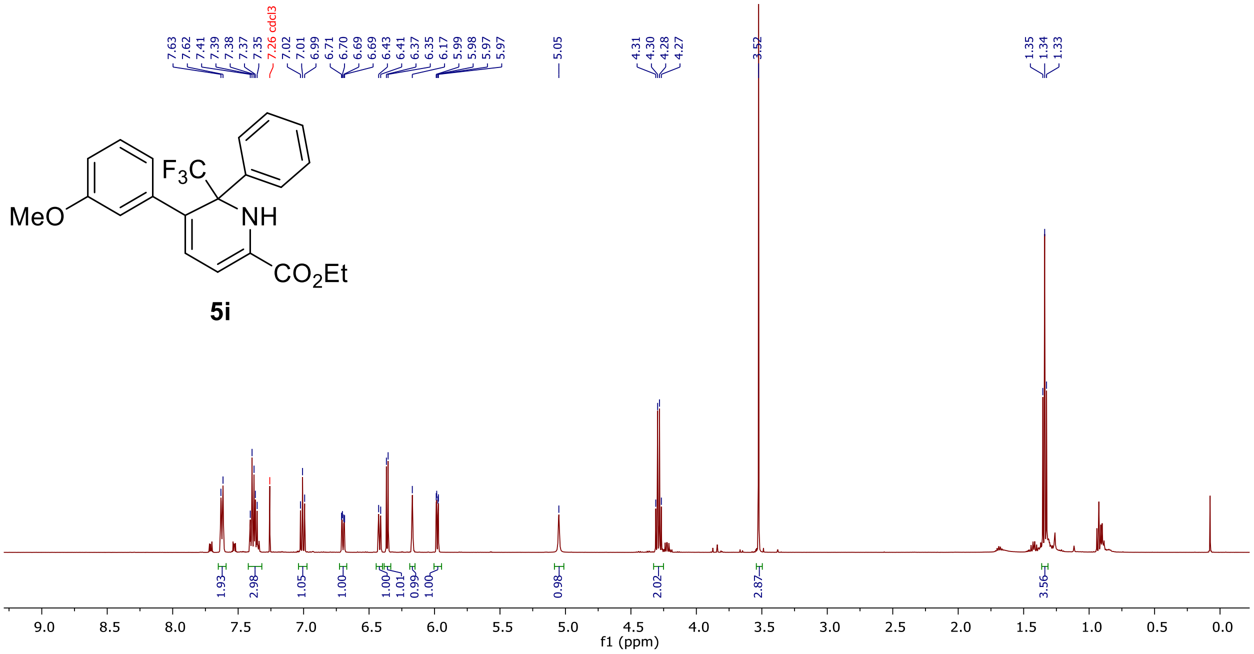

^{13}C NMR (126 MHz, $CDCl_3$)

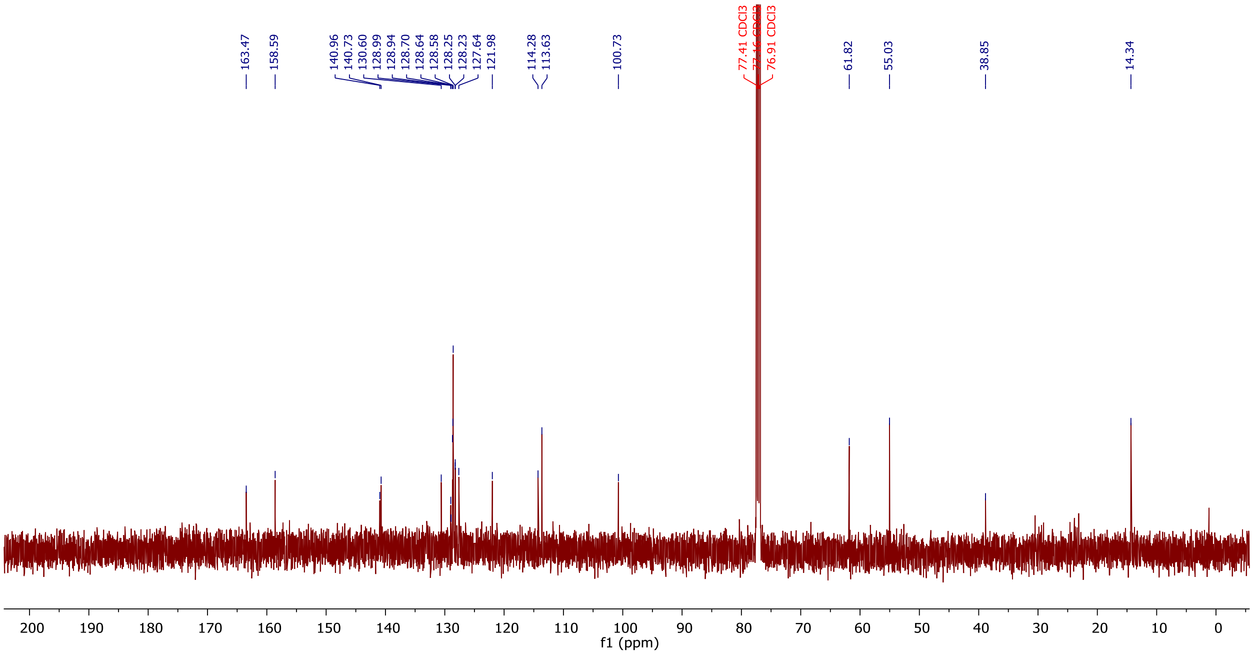

^{19}F NMR (470 MHz, $CDCl_3$)

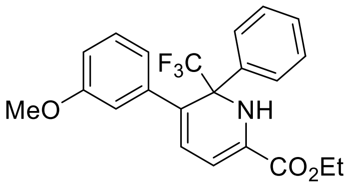

5i

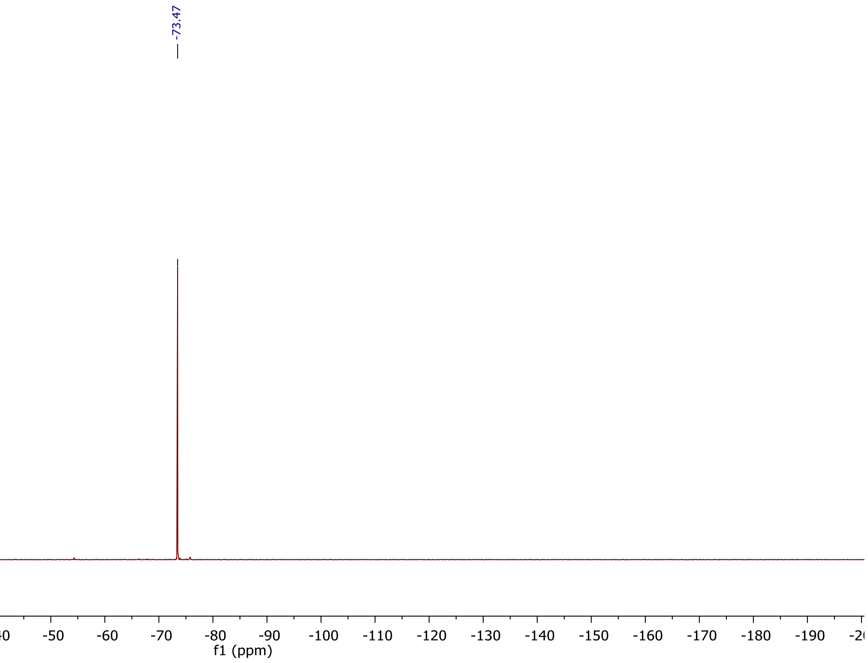

^{1}H NMR (500 MHz, $CDCl_3$)

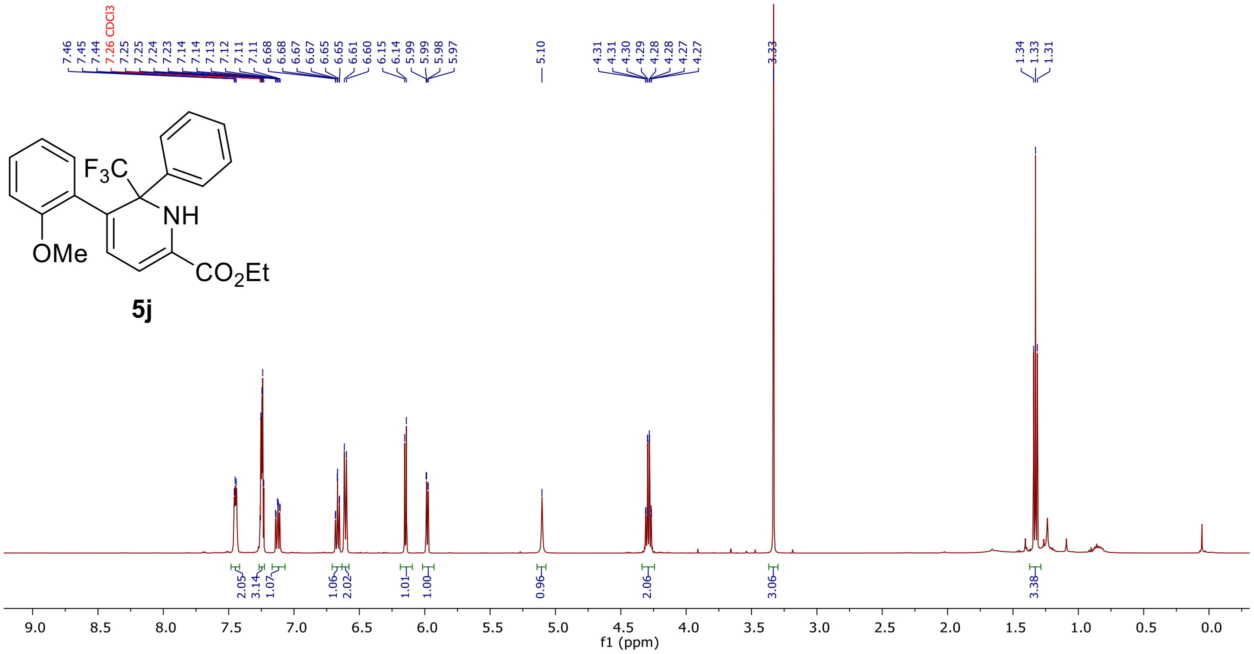

^{13}C NMR (126 MHz, $CDCl_3$)

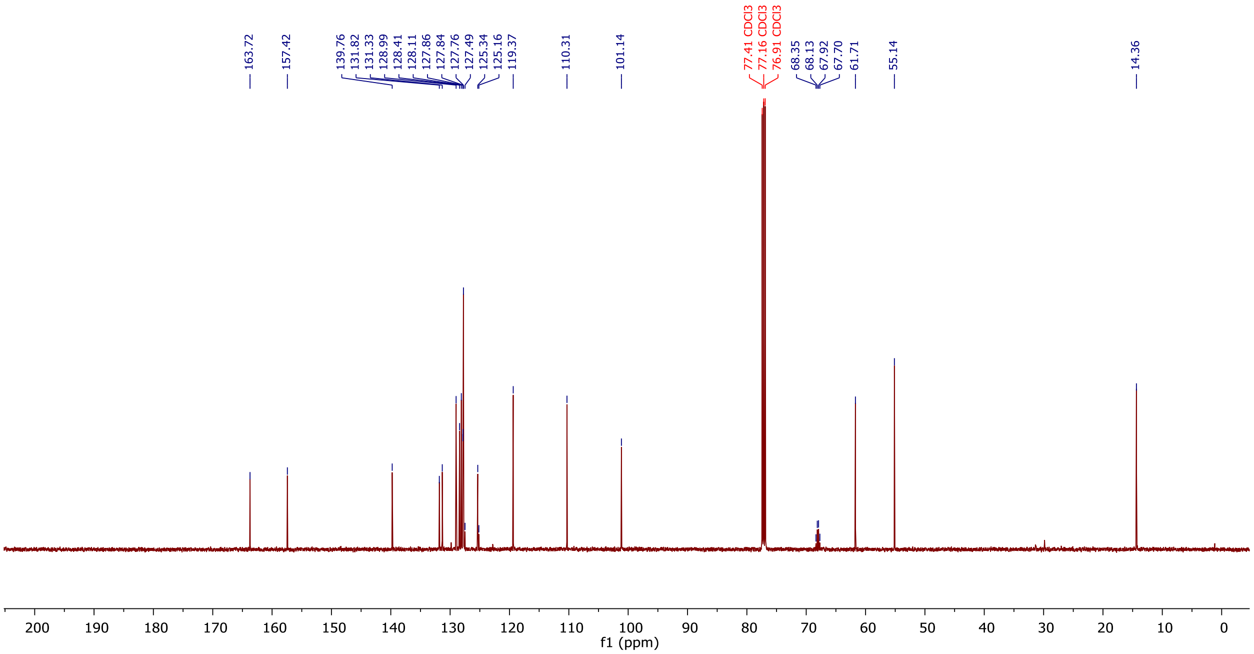

^{19}F NMR (470 MHz, $CDCl_3$)

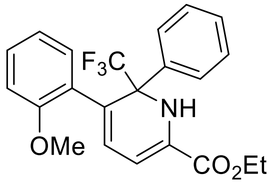

5j

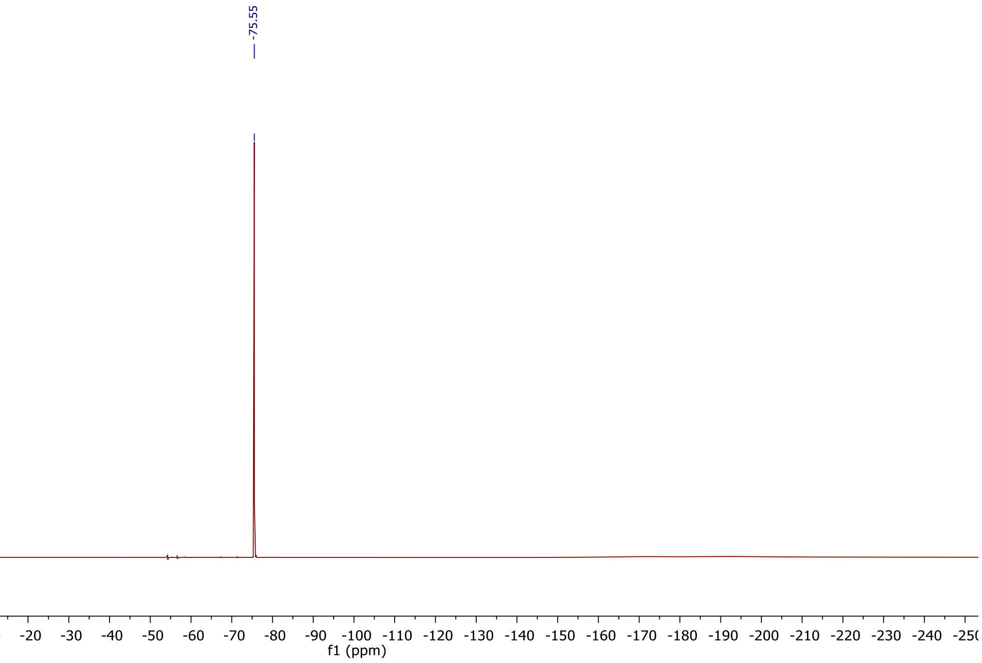

^{1}H NMR (500 MHz, $CDCl_3$)

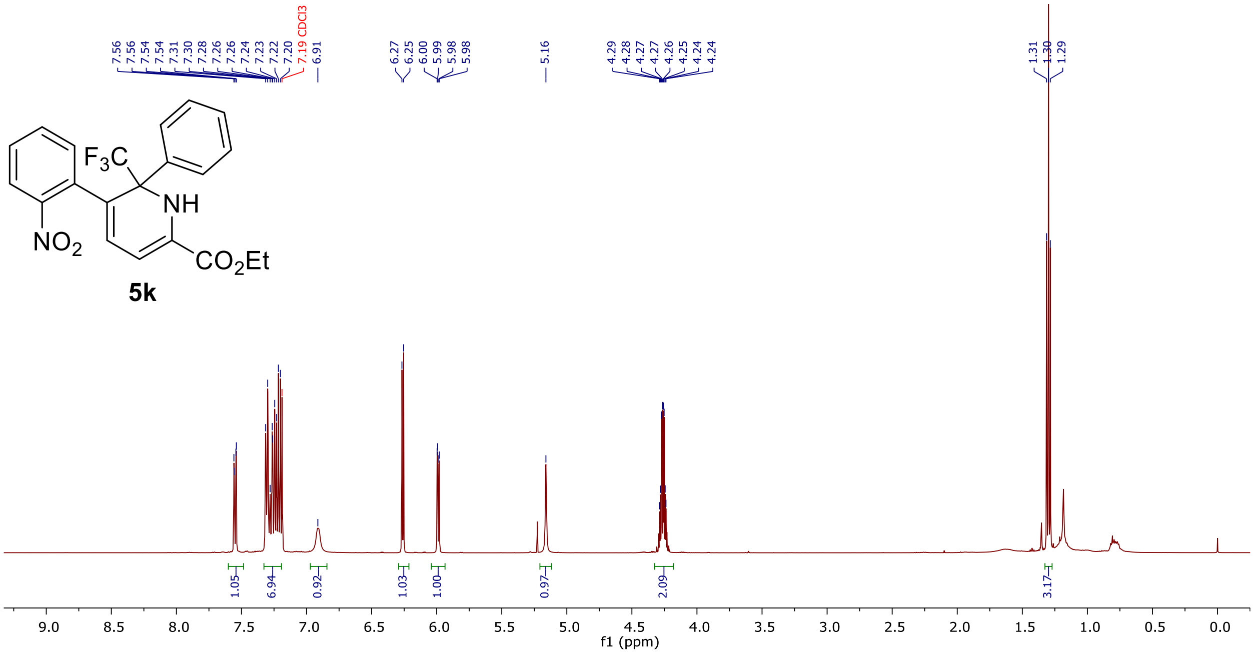

^{13}C NMR (126 MHz, $CDCl_3$)

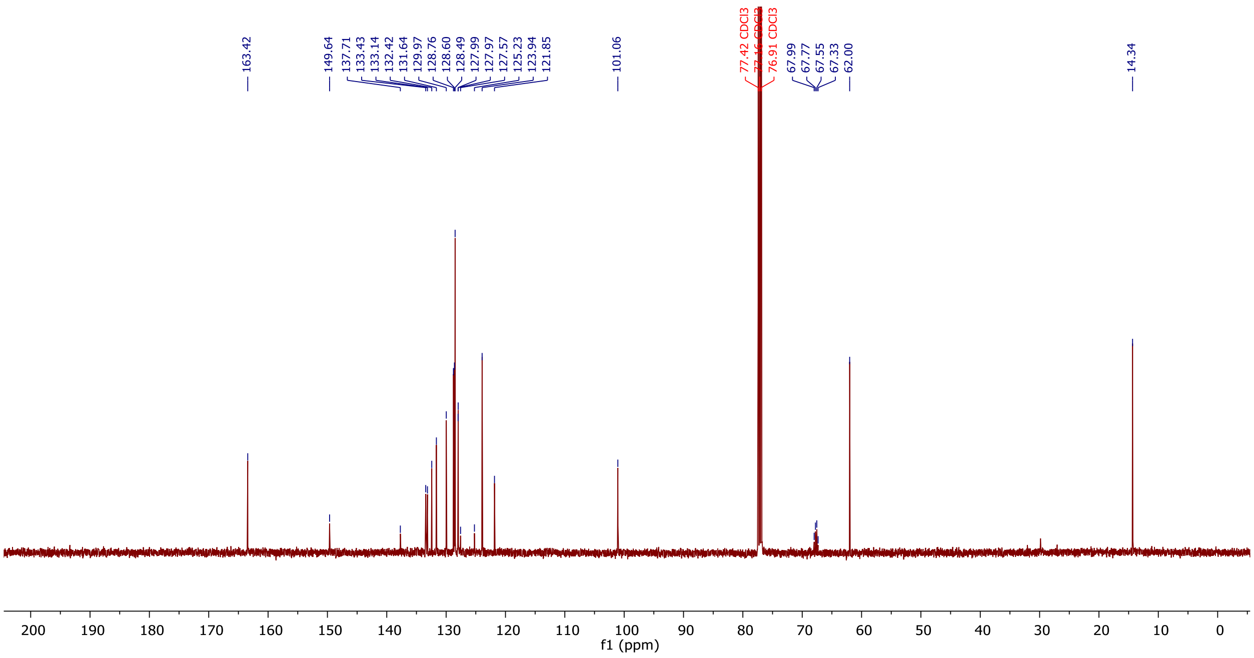

^{19}F NMR (470 MHz, $CDCl_3$)

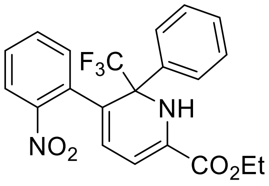

5k

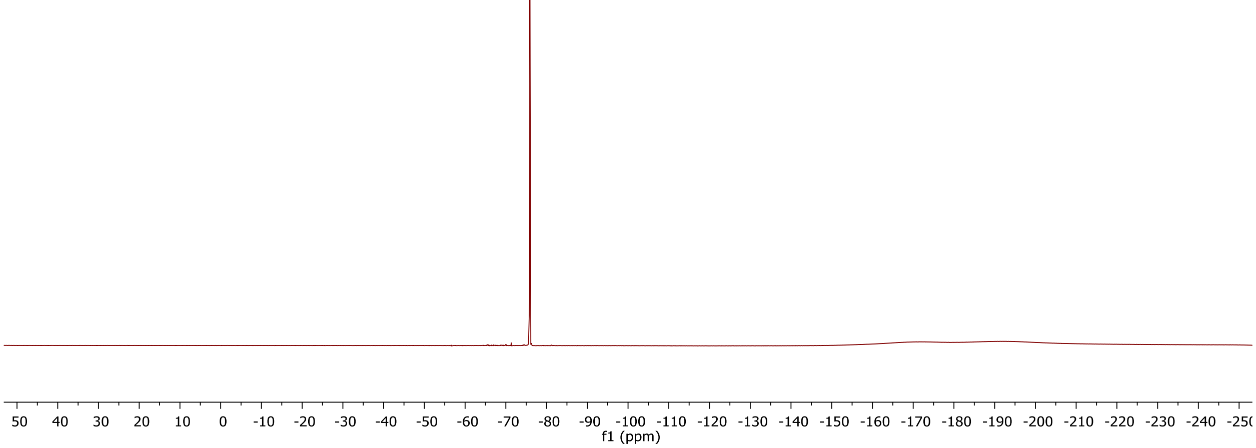

^{1}H NMR (500 MHz, $CDCl_3$)

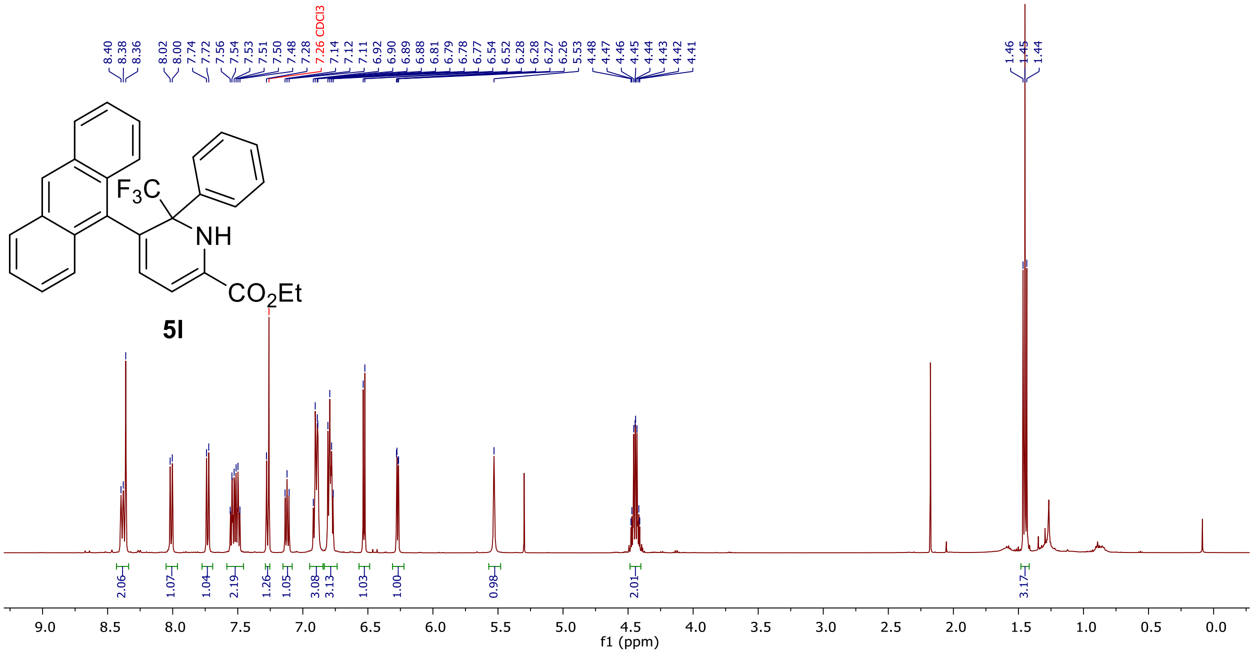

^{13}C NMR (126 MHz, $CDCl_3$)

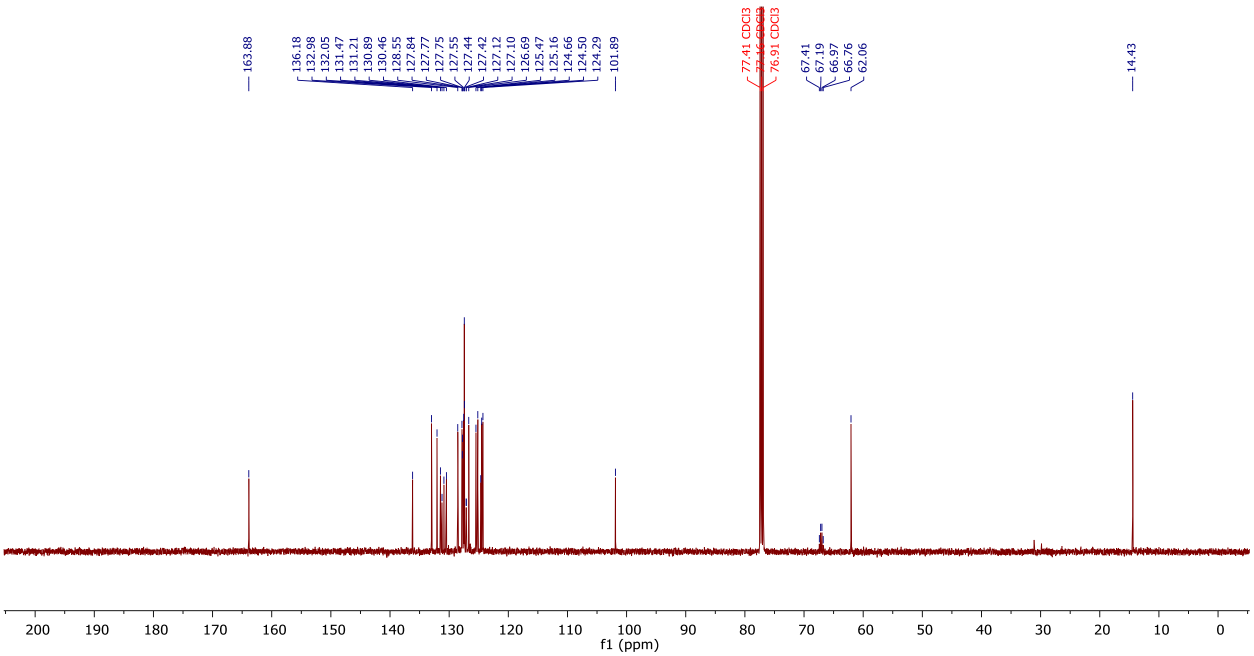

^{19}F NMR (470 MHz, $CDCl_3$)

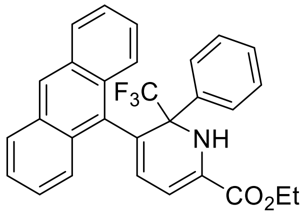

5l

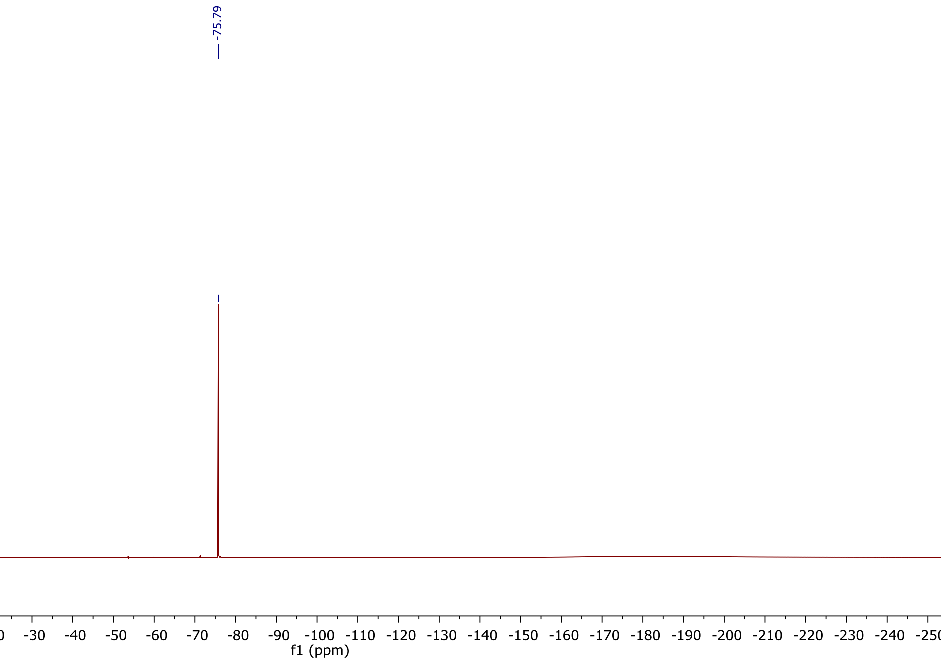

^{1}H NMR (500 MHz, $CDCl_3$)

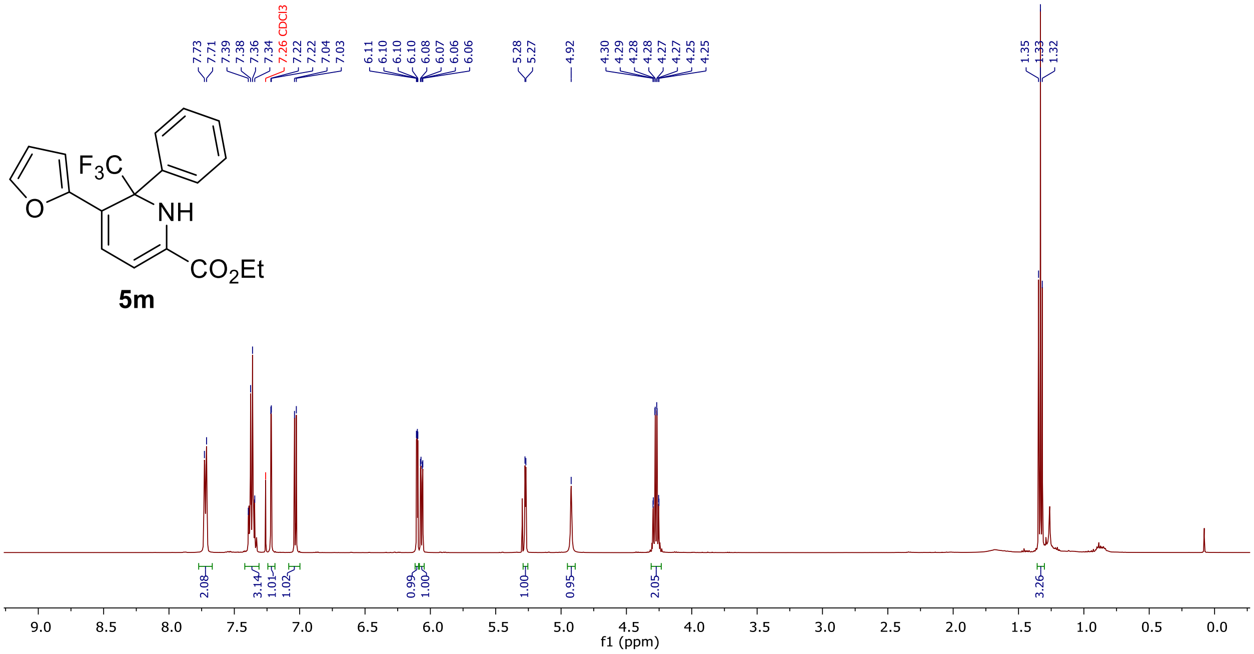

^{13}C NMR (126 MHz, $CDCl_3$)

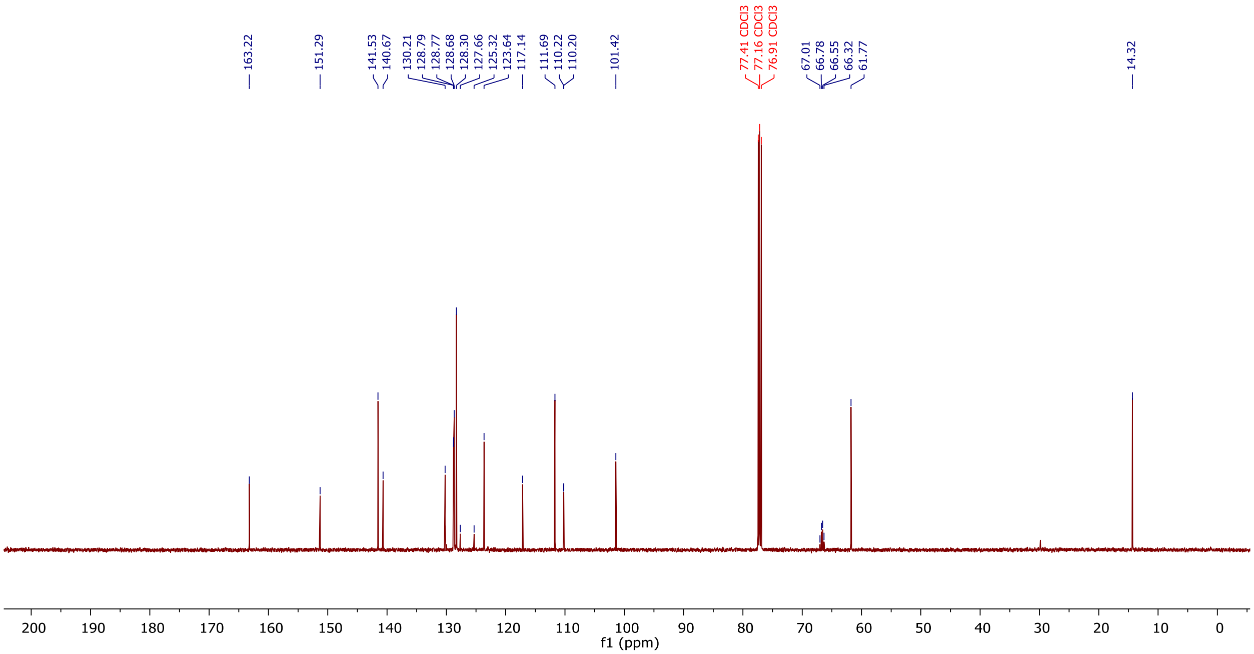

^{19}F NMR (470 MHz, $CDCl_3$)

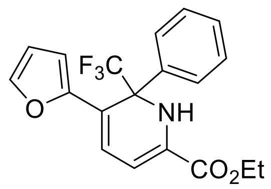

5m

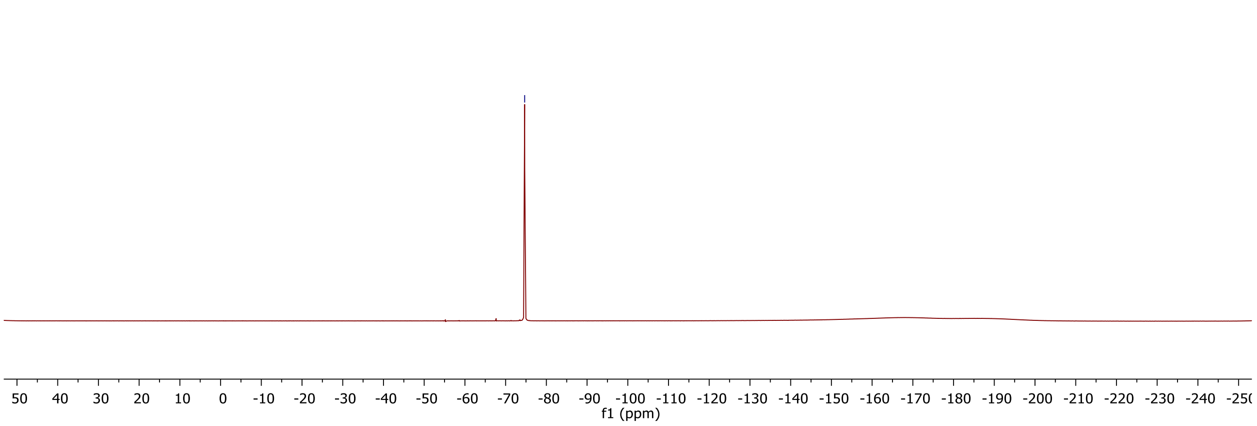

^{1}H NMR (500 MHz, $CDCl_3$)

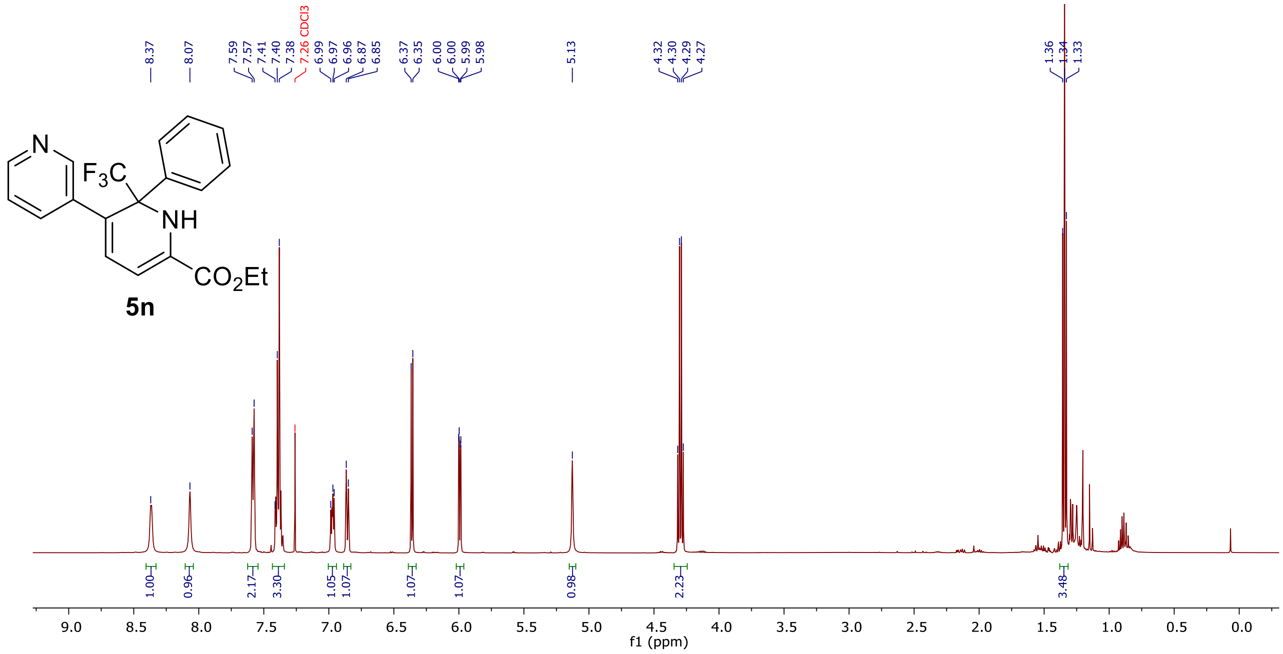

^{13}C NMR (126 MHz, $CDCl_3$)

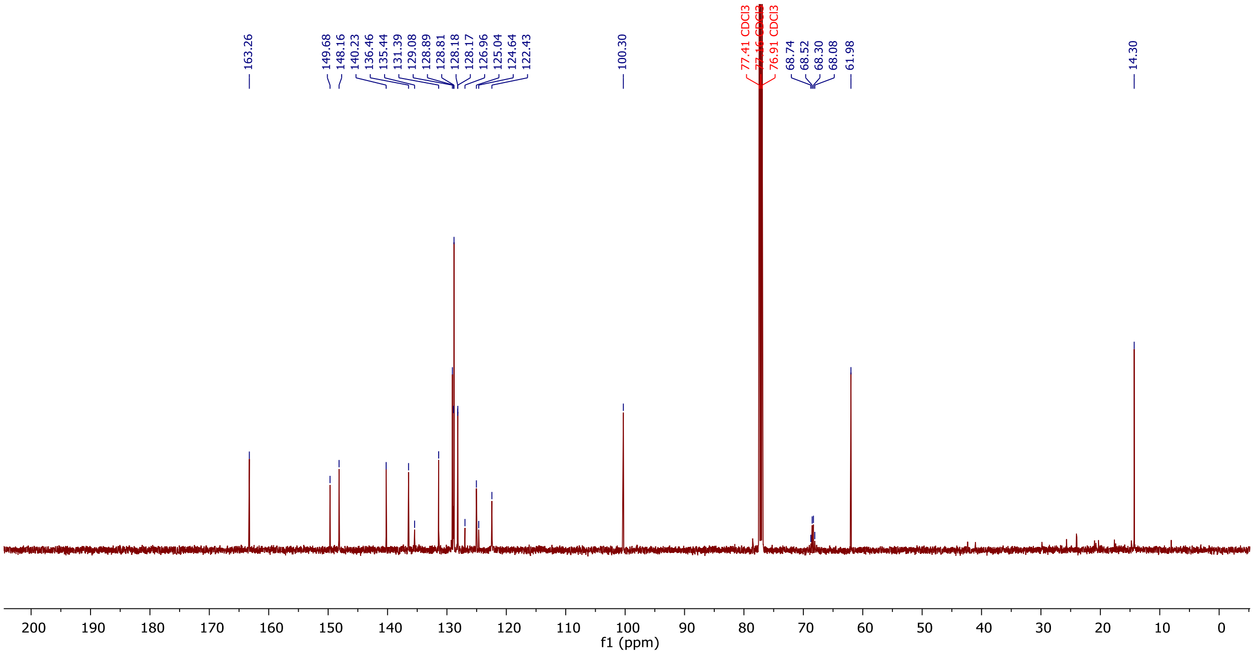

^{19}F NMR (470 MHz, $CDCl_3$)

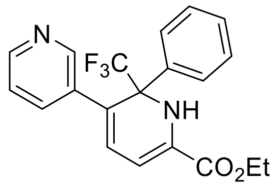

5n

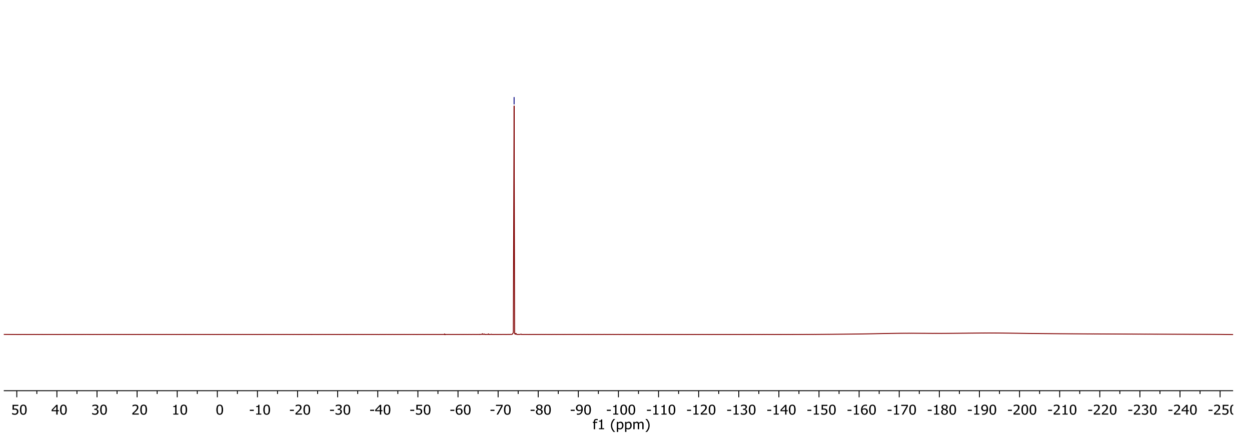

^{1}H NMR (500 MHz, $CDCl_3$)

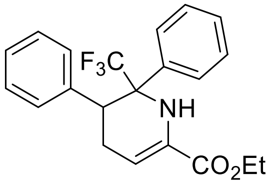

6a

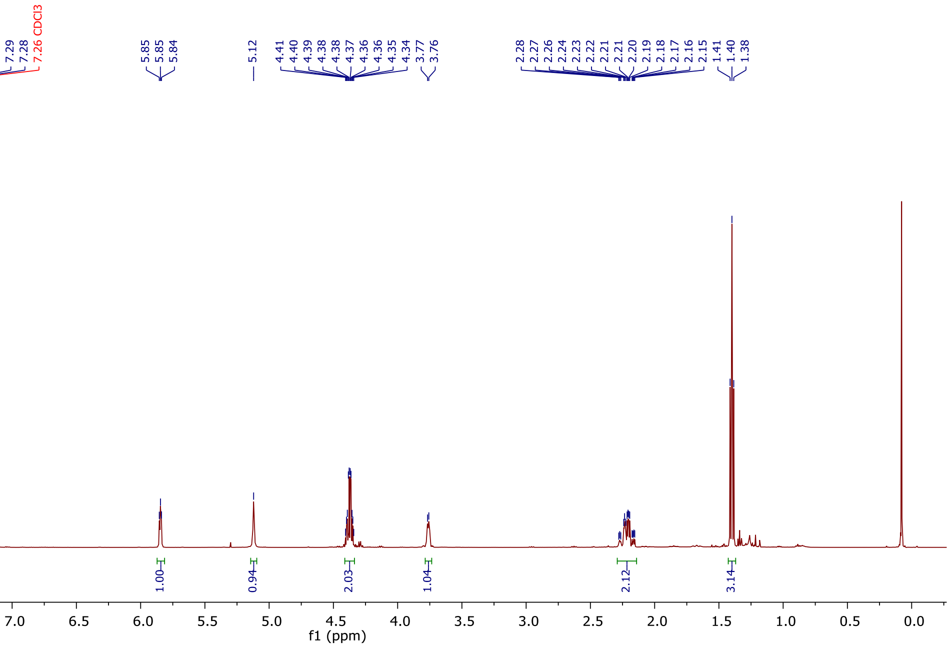

^{13}C NMR (126 MHz, $CDCl_3$)

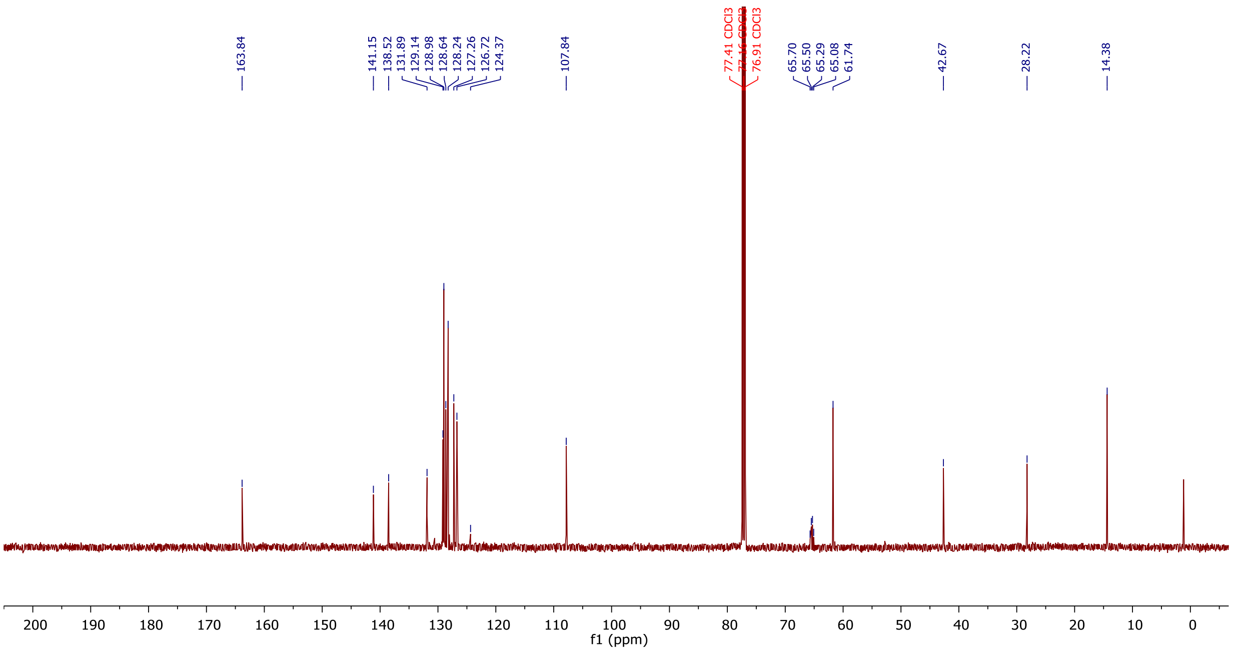

^{19}F NMR (470 MHz, $CDCl_3$)

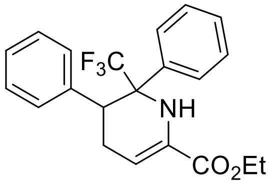

6a

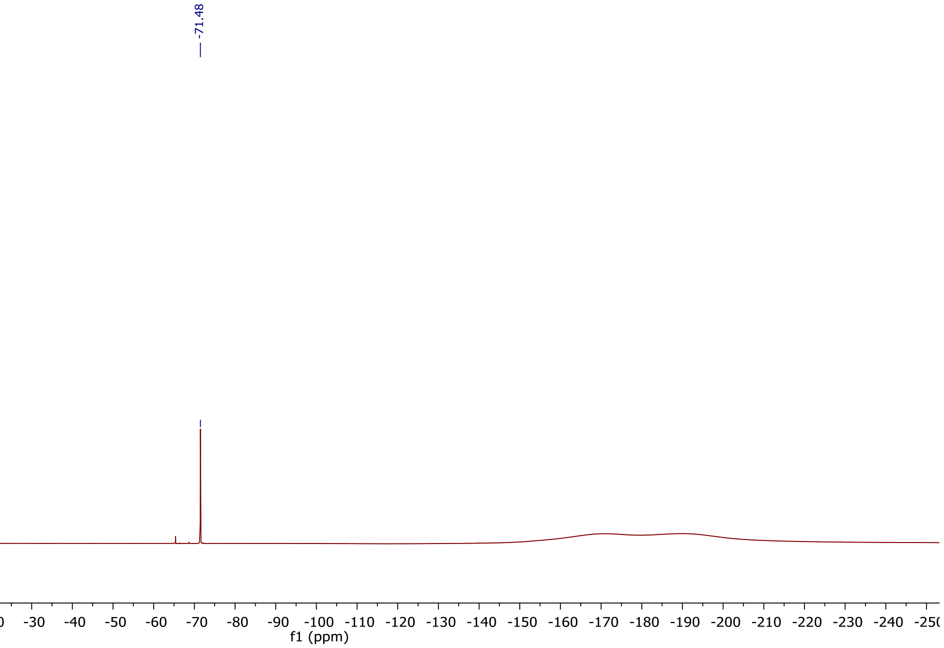

^{1}H NMR (500 MHz, $CDCl_3$)

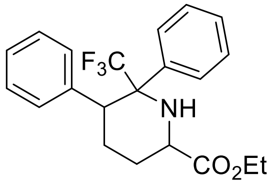

6b

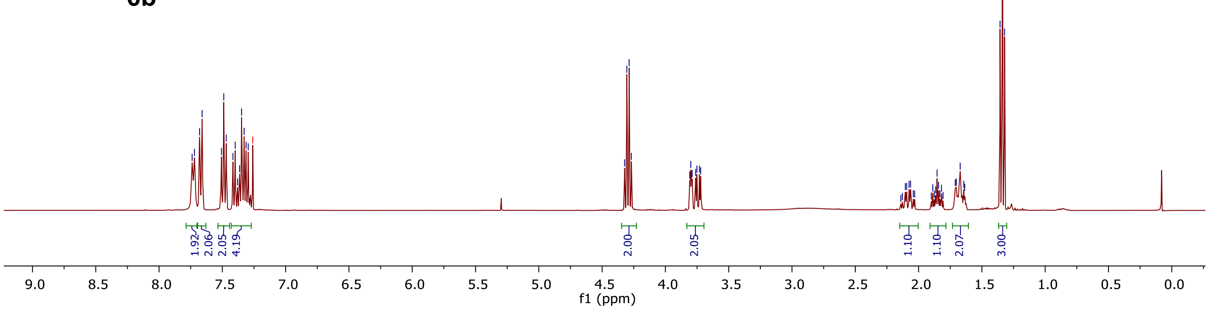

^{13}C NMR (126 MHz, $CDCl_3$)

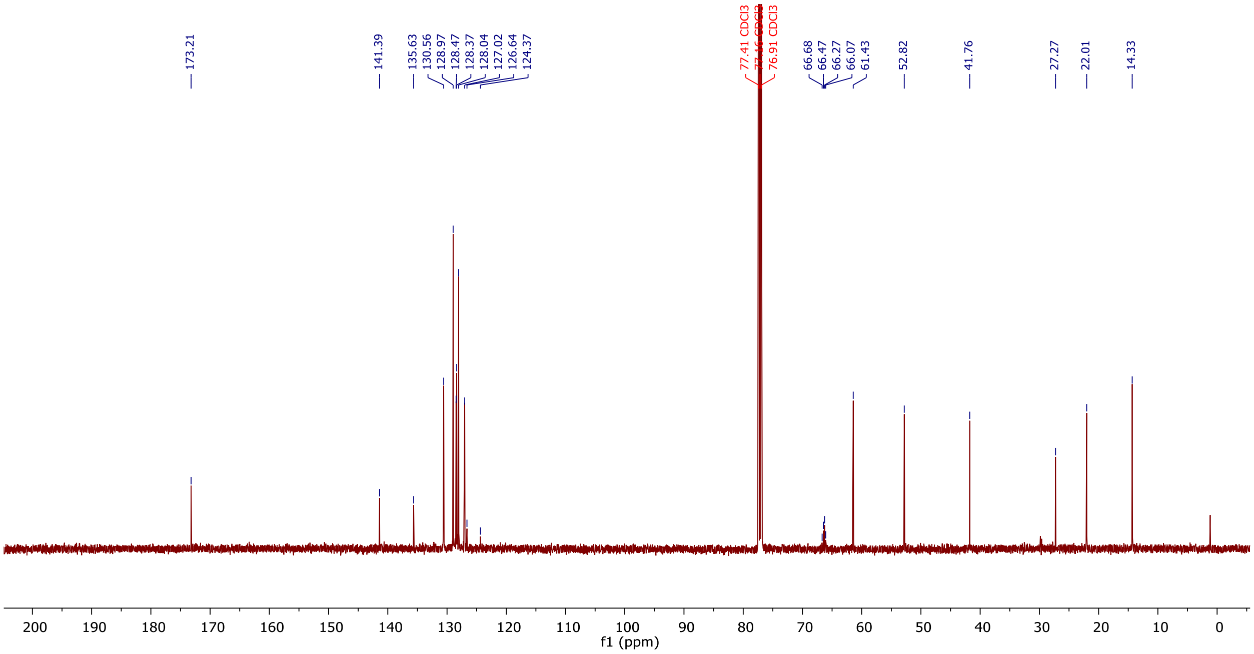

^{19}F NMR (470 MHz, $CDCl_3$)

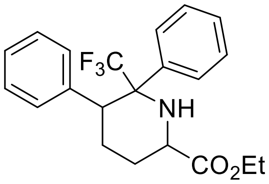

6b

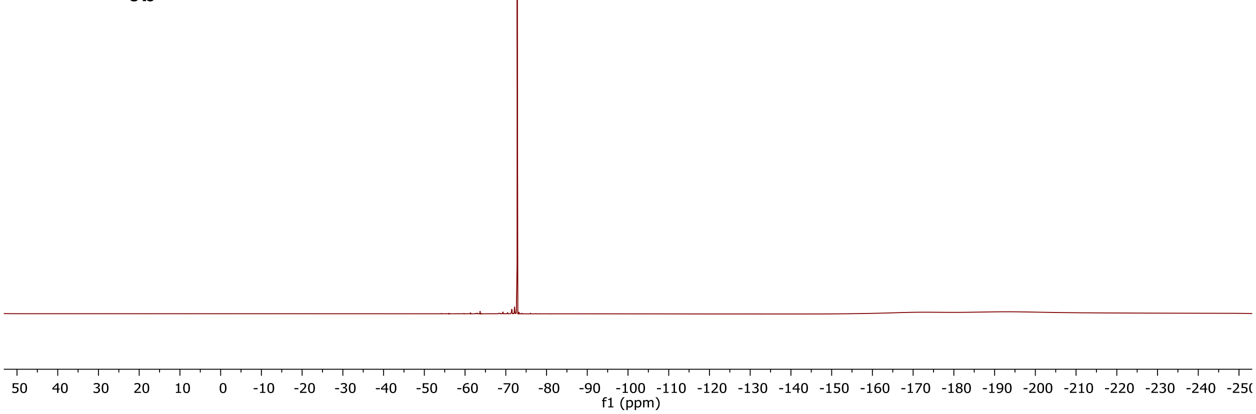

6. Copies of Selected Chiral HPLC Chromatograms

HPLC traces for **5b**: racemic top, enantioselective (catalyzed by (*R*)-**7c**)

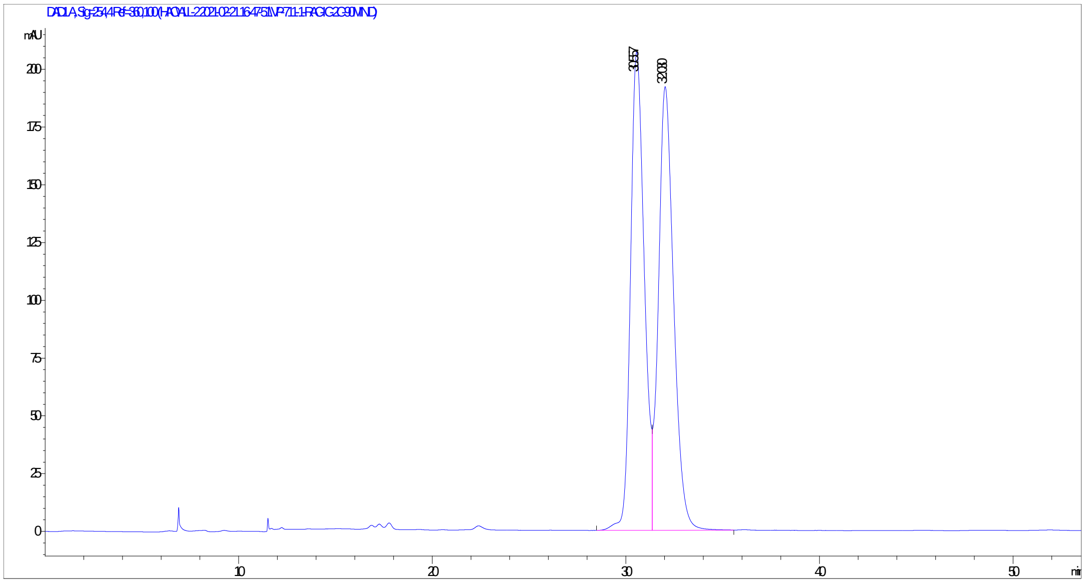

#	Time	Area	Height	Width	Area%	Symmetry
1	30.557	9803	203.6	0.8024	49.508	0.763
2	32.03	9998	188.9	0.8822	50.492	0.777

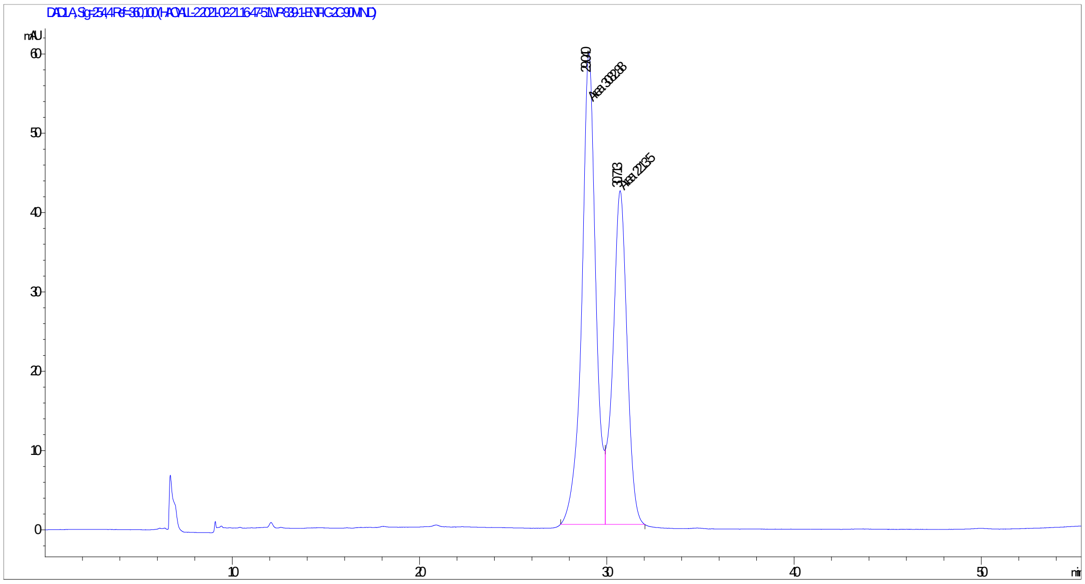

#	Time	Area	Height	Width	Area%	Symmetry
1	29.04	3082.9	59.5	0.8635	58.207	0
2	30.713	2213.5	42.1	0.8768	41.793	1.016

HPLC traces for **5b**: racemic top, enantioselective (catalyzed by (*R*)-**7d**)

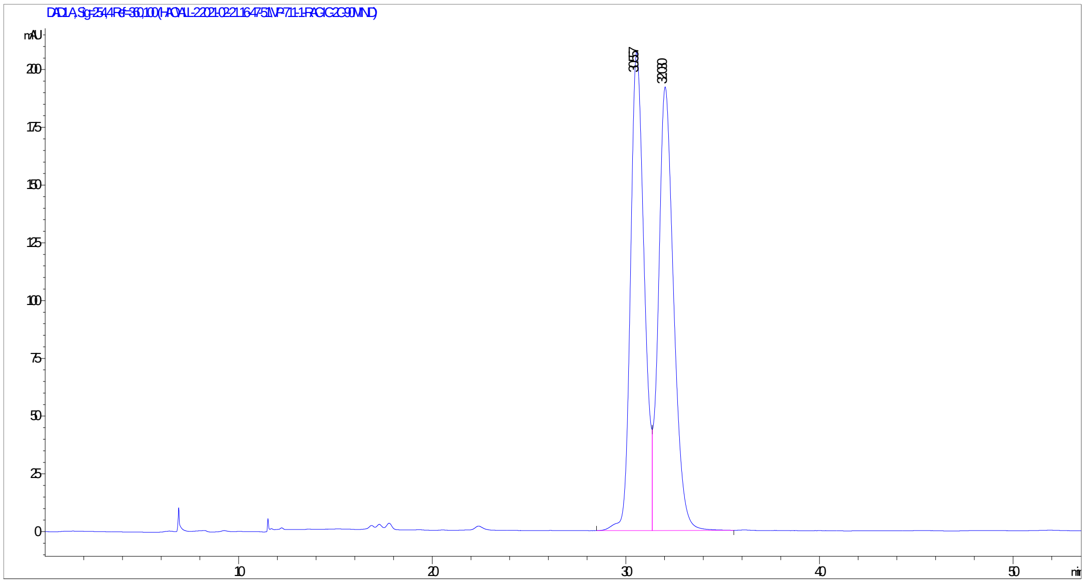

#	Time	Area	Height	Width	Area%	Symmetry
1	30.557	9803	203.6	0.8024	49.508	0.763
2	32.03	9998	188.9	0.8822	50.492	0.777

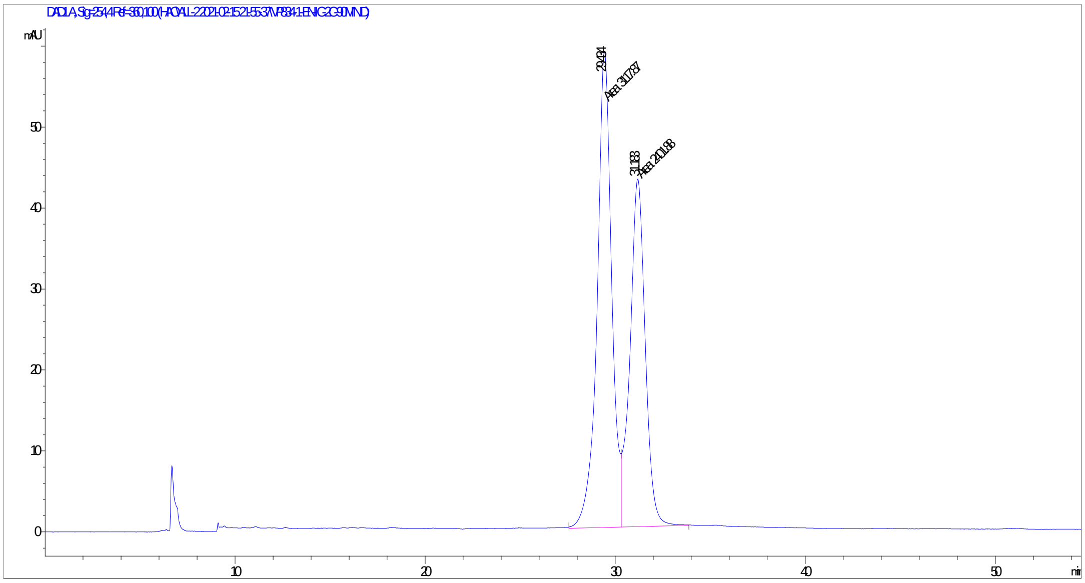

#	Time	Area	Height	Width	Area%	Symmetry
1	29.434	3117.9	58.7	0.8852	56.486	1.016
2	31.183	2401.9	42.9	0.9328	43.514	0.994

HPLC traces for **5b**: racemic top, enantioselective (catalyzed by (*R*)-**7e,** 60 °C)

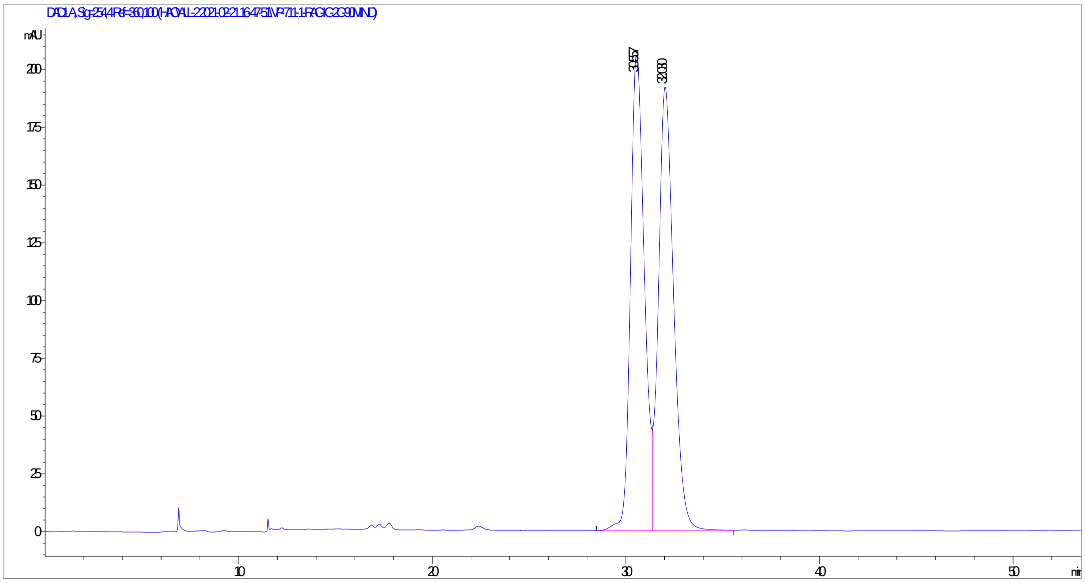

#	Time	Area	Height	Width	Area%	Symmetry
1	30.557	9803	203.6	0.8024	49.508	0.763
2	32.03	9998	188.9	0.8822	50.492	0.777

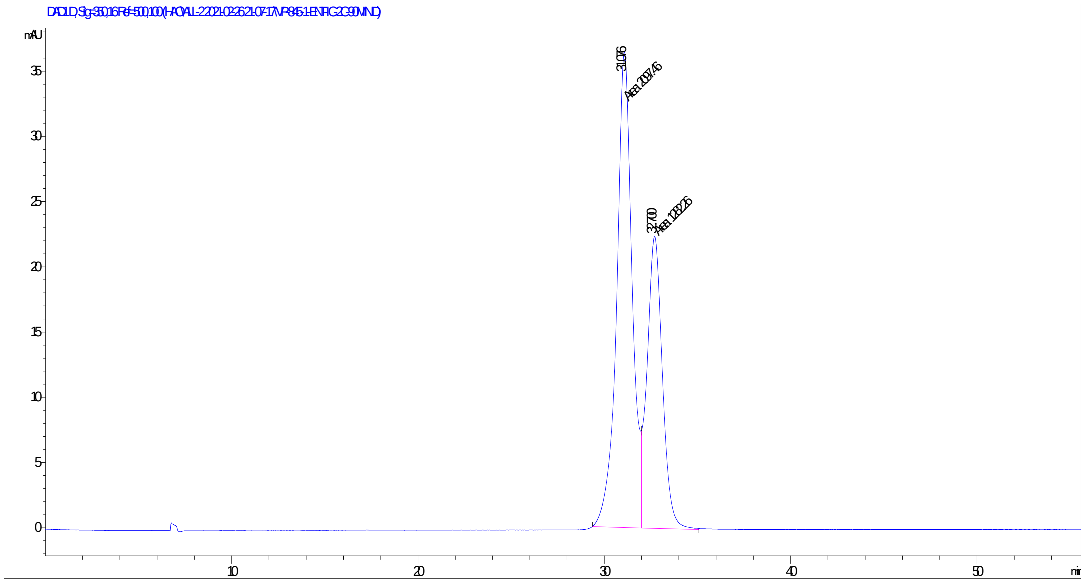

#	Time	Area	Height	Width	Area%	Symmetry
1	31.076	2097.5	36.5	0.9588	62.060	0
2	32.7	1282.3	22.4	0.9554	37.940	0.93

HPLC traces for **5b**: racemic top, enantioselective (catalyzed by (*R*)-**7e,** 50 °C)

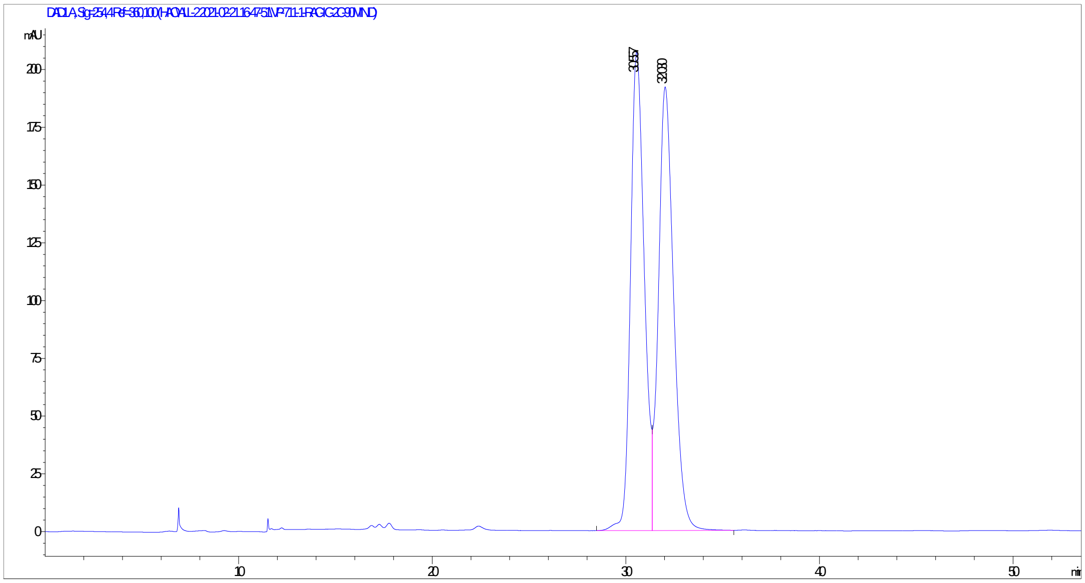

#	Time	Area	Height	Width	Area%	Symmetry
1	30.557	9803	203.6	0.8024	49.508	0.763
2	32.03	9998	188.9	0.8822	50.492	0.777

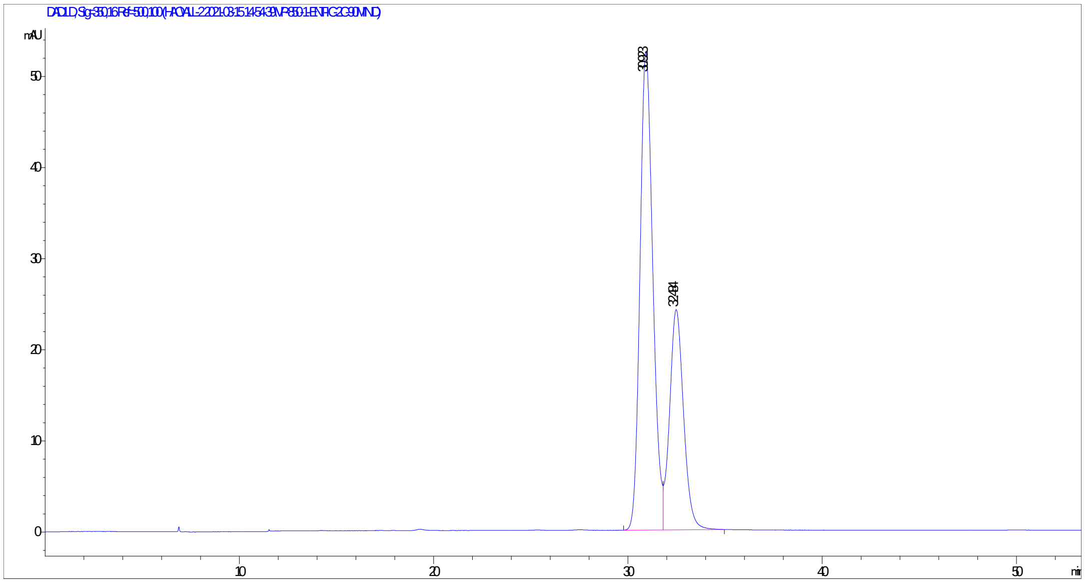

#	Time	Area	Height	Width	Area%	Symmetry
1	30.923	2411	52.5	0.7123	66.546	0.805
2	32.484	1212	24.2	0.763	33.454	0.845

7. References

(1) Henn, L.; Hickey, D. M. B.; Moody, C. J.; Rees, C. W. Formation of indoles, isoquinolines, and other fused pyridines from azidoacrylates. *J. Chem. Soc., Perkin Trans. 1* **1984**, 2189-2196.
(2) Conlon, I. L.; Drennen, B.; Lanning, M. E.; Hughes, S.; Rothhaas, R.; Wilder, P. T.; MacKerell Jr., A. D.; Fletcher, S. Rationally Designed Polypharmacology: α-Helix Mimetics as Dual Inhibitors of the Oncoproteins Mcl-1 and HDM2. *ChemMedChem* **2020,** *15*, 1691-1698.
(3) Chen, Z.-B.; Hong, D.; Wang, Y.-G. A Cascade Approach to Pyridines from 2-Azido-2,4-dienoates and α-Diazocarbonyl Compounds. *J. Org. Chem.* **2009,** *74*, 903-905.
(4) (a) Dong, H.; Shen, M.; Redford, J. E.; Stokes, B. J.; Pumphrey, A. L.; Driver, T. G. Transition Metal-Catalyzed Synthesis of Pyrroles from Dienyl Azides. *Org. Lett.* **2007,** *9*, 5191-5194; (b) Shou, W. G.; Li, J.; Guo, T.; Lin, Z.; Jia, G. Ruthenium-Catalyzed Intramolecular Amination Reactions of Aryl- and Vinylazides. *Organometallics* **2009,** *28*, 6847-6854.
(5) Pinna, G.; eacute; rard, A.; egrave; Loriga, G.; Murineddu, G.; Grella, G.; Mura, M.; Vargiu, L.; Murgioni, C.; La Colla, P. Synthesis and Anti-HIV-1 Activity of New Delavirdine Analogues Carrying Arylpyrrole Moieties. *Chem. Pharm. Bull.* **2001,** *49*, 1406-1411.
(6) Wang, L.; Hubert, J. A.; Lee, S. J.; Pan, J.; Qian, S.; Reitman, M. L.; Strack, A. M.; Weingarth, D. T.; MacNeil, D. J.; Weber, A. E.; Edmondson, S. D. Discovery of pyrimidine carboxamides as potent and selective CCK1 receptor agonists. *Bioorg. Med. Chem. Lett.* **2011,** *21*, 2911-2915.
(7) Farney, E. P.; Yoon, T. P. Visible-Light Sensitization of Vinyl Azides by Transition-Metal Photocatalysis. *Angew. Chem. Int. Ed.* **2014,** *53*, 793-797.
(8) Dolomanov, O. V.; Bourhis, L. J.; Gildea, R. J.; Howard, J. A. K.; Puschmann, H. OLEX2: a complete structure solution, refinement and analysis program. *J. Appl. Crystallogr.* **2009,** *42*, 339-341.
(9) Sheldrick, G. SHELXT - Integrated space-group and crystal-structure determination. *Acta Crystallographica Section A* **2015,** *71*, 3-8.
(10) Sheldrick, G. Crystal structure refinement with SHELXL. *Acta Crystallographica Section C* **2015,** *71*, 3-8.
(11) Farrugia, L. J. ORTEP-3 for Windows - a version of ORTEP-III with a Graphical User Interface (GUI). *J. Appl. Crystallogr.* **1997,** *30*, 565-565.
(12) Beard R.; Donello, J. E.; Yuan, H.; Liu, X. (13.03.2008): Heteroaromatic Compounds Having Sphingosine-1-Phosphate (Sip) Receptor Agonist And/Or Antagonist Biological Activity. WO 2008/030843, World Intellectual Property Organization
(13) Hemetsberger, H.; Spira, I.; Schoenfelder, W. Synthesis and Thermolysis of α-azido-α,γ-dienoic esters. *J. Chem. Res. Synop.* **1977**, *10*, 247.
